# Supplementary material for: The impact of the COVID-19 pandemic on the research activity and working experience of clinical academics, with a focus on gender and ethnicity: a qualitative study in the UK
Source: BMJ Open. 2022 Jun 8;12(6):e057655. doi: 10.1136/bmjopen-2021-057655 (PMC9184994; doi:10.1136/bmjopen-2021-057655)
Supplement: Supplementary data [file bmjopen-2021-057655supp001.pdf]

Appendix 1: Interview and example audio diary prompts for participants during COVID-19

Example interview stems for participants during COVID-19
Semi-structured approach thus will be guided by participants.<br>1. **Introductions**<br><br>2. **Please describe your role and career path to date -** *both clinical and academic*<br><br>3. **What are your experiences of clinical academia?**<br><br>4. **What factors impacted upon your decision to pursue (or not pursue) a career in clinical academia?**<br>• *What factors make clinical academia an attractive career?*<br><br>5. **What has your experience with awards/ funding been like? (if any)**<br>• *Application process? (facilitators/barriers)*<br>• *Support through process*<br>• *Why did/didn't continue? (e.g. after PhD why didn't do lectureship?)*<br>• *If not applied, why not?*<br>• *Did this experience influence your choice in career pathway?*<br><br>6. **What barriers and enablers have you faced within clinical academia?**<br>• *What made you continue/not continue?*<br>• What stops people returning to clinical academia?<br>• How do you negotiate the 2 career paths?<br>• What goes wrong in institutions that funders can help with?<br>• What worked well/ less well with other funders you have experienced?<br>• Impact of the COVID-19 pandemic<br><br>7. **What could be done to improve equal representation within clinical academia?**<br><br>8. **Describe any interventions to promote access to clinical academia or reduce discrimination that you have experienced.**<br>• *What worked?*<br>• *Given your context, what would you recommend?*<br><br>9. **What existing or new interventions could help to reduce attrition in clinical academic careers?**<br><br>10. **How can organisations support trainees and clinical academics in their career decisions and academic pathways?**
**Example audio diary prompts for participants during COVID-19**
Participants were able to reflect on all prompts any situation pertaining to Clinical Academia that they wished. Prompts were broad and participants were informed of the interest in protected characteristics.

How has COVID-19 impacted upon your work as a clinical academic?	What have you heard about funding (opportunities, threats, remit etc) post COVID-19?	Has COVID-19 had a detrimental impact on or changed your career plans?
How has the expectation of the impact of COVID-19 differed from the reality?	How have your protected characteristics impacted upon your experience of clinical academia during COVID-19?	BAME participants: Has the emerging data on the enhanced risk to the BAME workforce from COVID-19 had any impact on you?
Has COVID-19 had a detrimental impact on or changed your research?	What barriers and enablers have you faced during COVID-19?	Parents/ carers: How have your responsibilities impacted upon your ability to work?
