# Supplementary material for: The impact of the COVID-19 pandemic on the research activity and working experience of clinical academics, with a focus on gender and ethnicity: a qualitative study in the UK
Source: BMJ Open. 2022 Jun 8;12(6):e057655. doi: 10.1136/bmjopen-2021-057655 (PMC9184994; doi:10.1136/bmjopen-2021-057655)
Supplement: Author's manuscript [file bmjopen-2021-057655.draft_revisions.pdf]

BMJ Open is committed to open peer review. As part of this commitment we make the peer review history of every article we publish publicly available.

When an article is published we post the peer reviewers' comments and the authors' responses online. We also post the versions of the paper that were used during peer review. These are the versions that the peer review comments apply to.

The versions of the paper that follow are the versions that were submitted during the peer review process. They are not the versions of record or the final published versions. They should not be cited or distributed as the published version of this manuscript.

BMJ Open is an open access journal and the full, final, typeset and author-corrected version of record of the manuscript is available on our site with no access controls, subscription charges or pay-per-view fees (http://bmjopen.bmj.com).

If you have any questions on BMJ Open's open peer review process please email info.bmjopen@bmj.com

BMJ Open

The COVID-19 pandemic: A disaster for clinical academia. Where are all the women?

Journal:	*BMJ Open*
Manuscript ID	bmjopen-2021-057655
Article Type:	Original research
Date Submitted by the Author:	25-Sep-2021
Complete List of Authors:	Finn, Gabrielle; The University of Manchester, Division of Medical Education, School of Medical Sciences, Faculty of Biology, Medicine and Health<br>Crampton, Paul; Hull York Medical School, Health Professions Education Unit<br>Buchanan , John; Barts and The London School of Medicine and Dentistry, Centre for Education and Innovation<br>Balogun, Abisola Olatokunbo; Hull York Medical School, Health Professions Education Unit<br>Tiffin, Paul; University of York, Health Sciences<br>Morgan, Jessica; University of York, Centre for Reviews and Dissemination; Leeds Teaching Hospitals NHS Trust, Department of Paediatric Oncology<br>Taylor , Ellie; Hull York Medical School, Health Professions Education Unit<br>Kehoe, Amelia; Hull York Medical School, Health Professions Education Unit<br>Soto, Carmen; British Medical Association
Keywords:	COVID-19, QUALITATIVE RESEARCH, MEDICAL EDUCATION & TRAINING

SCHOLARONE™
Manuscripts

I, the Submitting Author has the right to grant and does grant on behalf of all authors of the Work (as defined in the below author licence), an exclusive licence and/or a non-exclusive licence for contributions from authors who are: i) UK Crown employees; ii) where BMJ has agreed a CC-BY licence shall apply, and/or iii) in accordance with the terms applicable for US Federal Government officers or employees acting as part of their official duties; on a worldwide, perpetual, irrevocable, royalty-free basis to BMJ Publishing Group Ltd ("BMJ") its licensees and where the relevant Journal is co-owned by BMJ to the co-owners of the Journal, to publish the Work in this journal and any other BMJ products and to exploit all rights, as set out in our licence.

The Submitting Author accepts and understands that any supply made under these terms is made by BMJ to the Submitting Author unless you are acting as an employee on behalf of your employer or a postgraduate student of an affiliated institution which is paying any applicable article publishing charge ("APC") for Open Access articles. Where the Submitting Author wishes to make the Work available on an Open Access basis (and intends to pay the relevant APC), the terms of reuse of such Open Access shall be governed by a Creative Commons licence – details of these licences and which Creative Commons licence will apply to this Work are set out in our licence referred to above.

Other than as permitted in any relevant BMJ Author's Self Archiving Policies, I confirm this Work has not been accepted for publication elsewhere, is not being considered for publication elsewhere and does not duplicate material already published. I confirm all authors consent to publication of this Work and authorise the granting of this licence.

For peer review only

The COVID-19 pandemic: A disaster for clinical academia. Where are all the women?

Gabrielle M Finn[1], Paul E S Crampton[2], John Buchanan[3], Abisola Balogun-Katung[2], Paul A. Tiffin[4], Jessica E Morgan[5,6], Ellie Taylor[2], Amelia Kehoe[2] and Carmen Soto[7]

Affiliations

1. Division of Medical Education, School of Medical Sciences, Faculty of Biology, Medicine and Health, The University of Manchester, M13 9NT
2. Health Professions Education Unit, Hull York Medical School, University of York, York, UK, YO10 5DD
3. Queen Mary University London, Barts Health NHS Trust
4. Mental Health and Addiction Research Group, Department of Health Sciences, University of York, York, UK, YO10 5DD
5. Centre for Reviews and Dissemination, University of York, York, UK, YO10 5DD
6. Department of Paediatric Oncology, Leeds Children's Hospital, Leeds, UK, LS1 3EX
7. British Medical Association, London, WC1H 9JP

Word count: 5582

For peer review only

The COVID-19 pandemic: A disaster for clinical academia. Where are all the women?

Abstract

Objective: To investigate the impact of the COVID-19 pandemic on the research activity and working experience of clinical academics, with a focus on gender and ethnicity.

Design: Semi-structured interviews and cross-sectional qualitative audio and written diary study.

Setting: A national UK study within clinical academia.

Participants: 82 clinical academics working in medicine and dentistry across all career stages ranging from academic clinical fellows, and doctoral candidates to professors.

Methods: Qualitative semi-structured interviews and longitudinal audio and written diary data were collected over an eight-month period between January-September 2020 (inclusive). Data were thematically analysed.

Results: Four major themes were identified in relation to the initial impact of COVID-19 on CAs; (1) opportunities, (2) barriers, (3) personal characteristics and social identity and (4) fears and uncertainty. COVID-19 presented opportunities for new avenues of research. Barriers included access to resources to conduct research and the increasing teaching demands. One of the most prominent sub-themes within 'personal characteristics' was that of the perceived negative impact of the pandemic on the work of female CAs. This was attributed to inequalities experienced in relation to childcare provision and research capacity. Participants described differential experiences based upon their gender and ethnicity, noting intersectional identities.

Conclusions: The disruption brought by COVID-19 has had a substantial impact on CAs in regard to their workload, future career intentions, and mental health. Our study elucidates the direct and systemic discrimination that creates barriers to women's career trajectories in clinical academia. A flexible, strategic response that supports CAs in resuming their training and research is required. Interventions are needed to mitigate the potential lasting impact on CA capacity from the pandemic, and the potential for the loss of women from this valuable workforce.

Article Summary

- Our findings suggest that the ongoing COVID-19 pandemic is leading to a continued decline in the progression of female clinical academics due to the reinforcement of gender stereotypes, particularly within the home.
- Support from institutions and funders in items of flexible working, valued teaching portfolios, protected research time and extensions could help mitigate the impact of COVID-19 on clinical academics who are uniquely positioned to tackle key research problems during the pandemic.

For peer review only

Strengths and Limitations

- This study used both audio and written diary entries as well as in-depth interviews, which provided a more comprehensive picture while capturing the complexity of the experiences of clinical academics.
- The use of video conferencing and telephone interviews provided flexibility which allowed for more data to be collected, particularly at the start of the pandemic when face-to-face data collection methods would not have been feasible.
- Our findings are based on data from a UK sample, which may be perceived as limited. We balanced this out by having a large sample size.
- The research team consisted of clinical academics from various backgrounds at various stages of their careers who were able to fact check data collected.
- The research explored sensitive issues around experiences of clinical academics. The use of audio and written diary entries and in-depth interviews require self-reporting and therefore may have biased the findings.

Introduction

The workforce presence of clinical academics is vital to healthcare to ensure that scientific knowledge underpins advances in medical education, translational research and patient outcomes. Their low number and lack of senior pipeline progression is alarming, particularly so for under-represented groups including women and ethnic minorities.[1] The COVID-19 pandemic has further exacerbated the situation through rapid redeployment, burnout, reinforcement of gender and racial stereotypes, and financial inequity.[2]

Over 1,500 academic trainees in England were estimated to be redeployed to clinical work during the COVID-19 pandemic[3]. This equates to more than 90% of those on the Integrated Academic Training (IAT) pathway.[3] Following the closure of high-risk aerosol-generating dental services, some junior hospital dentists and dental clinical academics were also redeployed to provide support care for hospital services.[4] The potential impact on trainees' clinical experience has been recognised.[5] In terms of formally monitoring and permitting advancement in postgraduate training, 'no fault' Annual Review of Competence Progression (ARCP) outcomes (10.1 and 10.2) have been introduced where acquisition of competencies has been delayed owing to COVID-19[6]. Meanwhile, there has been a research paradox whereby some research institutions have closed, while others have adapted and embraced COVID specific research [7]. Further issues highlight the consumption of academic resources that may prove ineffective or wasteful owing to haste, rapid redesign of online teaching materials, and inadequate infrastructure and design.[8] With the current prioritization of COVID-19 studies, new clinical trials have been paused [9] with detrimental impacts on clinical academics working on non-COVID research.

Prior to the pandemic, it was recognised that females in medicine tended to take on more 'service work' and had less protected time for research than their male counterparts.[4] In addition to low representation of minority groups, the lack of role models provides a further barrier. [5] Collectively, gender and racial stereotypes,

For peer review only

childcare and home-working all put further pressure on an already stretched workforce, with women now publishing less.[6, 7] Losing further women and minority groups from this fragile workforce could run the risk of long-term reduced diversity in medical education, less female-relevant clinical research and ultimately have a negative impact on patient outcomes, in addition to having less women visible in senior positions [8, 9].

We sought to explore discrimination based upon protected characteristics in the clinical academic workforce, at various stages of the pipeline during the pandemic. We aimed to understand the barriers, enablers and interventions to facilitate clinical academic careers, thereby to develop key recommendations to support and reduce attrition of these exceptionally talented individuals.

Methods

Ethics

Ethical approval for this study was obtained from the XXX Medical School Ethics Committee (ref: 19 32).

Context

Prior to the COVID-19 pandemic, the authors were engaged in a multi-funder research project exploring barriers and facilitators to a career in UK clinical academia[10, 11]. As data were iteratively analysed, the impact of COVID-19 on clinical academia progressively shaped the experiences of the participants and relevant themes emerged. In order to assess the impact of COVID-19, the data collected after the first confirmed case within the UK (29th January 2020) until the end of September 2020 were isolated for analysis. This period included the first period of significant national restrictions ("lockdown") from 26th March to a phased reopening in June.

This study was conducted within a social constructivism orientation, additionally informed by the multiple principles of feminist theory. Feminist theory holds a constructivist ontology.

Patient and Public Involvement

The study had a steering group which included Patient and Public representation, provided by Health Watch

Study design and recruitment

Qualitative interviews and longitudinal qualitative audio and written diary entries were utilised. Inclusion criteria specified participants were a doctor or dentist, based within the UK and working as or towards becoming a clinical academic. For the purposes of the study, a 'clinical academic' was defined by individuals themselves and how they perceived their roles. Recruitment was through various avenues; a website, emails sent on behalf of the research team by stakeholder organisations; social media recruitment; and 'snowballing'.

Data collection

For peer review only

Data were collected using (1) audio and written diary entries, and (2) semi-structured interviews. Diaries enabled participants to discuss their views on a wide range of factors related to clinical academic careers, and latterly their experiences of working during COVID-19. The diary method enabled researchers to collect 'novel' and timely data during the transition to lockdown phase, a critical moment in healthcare. Many diaries were recorded immediately after events, such as discriminatory remarks being made in a workplace. Participants were invited to submit audio diary entries (MP3 files) via email or attached to an encrypted message. Submissions were sent to a professional account linked to the project following best practice.[12] In order to promote engagement, participants were also permitted to write their diary entry.[12] Participants were able to submit diaries as frequently as they wished, each acknowledged by the researchers to maintain rapport and engagement. Participants were issued with a guide to follow which was informed by a concurrent systematic review.[9] The guide suggested frequencies of entries, instructions for submission and indicative content (Appendix 1). Reminder emails were sent monthly. Interviews were conducted via video conferencing or telephone with the use of a topic guide. Audio diary files and interviews were recorded and transcribed verbatim. All participants were given an information sheet and consent form prior to the recording.

Data analysis

Data were thematically analysed [13, 14]. Thematic analysis was chosen because it is an appropriate method for seeking to understand experiences, thoughts, or behaviours across a dataset [15]. The authors read the data in its entirety and developed a codebook as a tool to assist in the analysis of the large dataset [16]. The six-step process of thematic analysis was followed: (1) data familiarisation, (2) generating initial codes, (3) searching for themes, (4) reviewing themes, (5) defining and naming themes, and (6) producing the report.[14, 17] Both inductive and deductive approaches were taken, with deductive analysis based on existing theory including maternal wall bias, feminist theory and intersectionality.[18-20] Authors engaged in a process of negotiation to refine codes and themes, before utilising member checking with a subset of participants. Authors were reflexive, recording reflexive journals and acknowledging their biases and presuppositions. The research team consisted of clinicians and non-clinicians, clinical academics at varying stages, expert qualitative researchers to novices, females and males, and a mix of ethnicities. Since the study data were confined to a limited collection period during the pandemic, it was felt that this would not be of sufficient duration for meaningful temporal analysis. Instead researchers interpreted the data as being snapshots and in the moment reflections relating to the impact of the pandemic. In order to bring the experiences over time, cross-sectional themes together and demonstrate intersectionality case studies from two participants are provided (Box 1).

Results

Demographics

Eighty-two individuals participated, 30 provided audio diary data, 68 were interviewed, while 16 provided both. Table 1 provides an overview of the descriptive statistics of the participant sample and the data collected. The research team received 134 diary entries, with a mean of 5 entries per participant.

TABLE 1: Demographic breakdown of participants

	Audio Diaries		Interviews	
**Profession**	**Total (n=30)**	**%**	**Total (n=68)**	**%**
Medicine	24	80.0	57	83.8
Dentistry	6	20.0	11	16.2
**Mean Age**	39		40	
**Age Range**	27 - 74		28 - 51	
**Gender**				
Male	10	33.3	28	41.2
Female	20	66.6	40	58.8
**Predominant Clinical Work Area**				
Primary	7	23.3	19	27.9
Secondary	11	36.7	30	44.1
Tertiary	12	40.0	17	25.0
Did not disclose	0	0	1	1.5
**Employment Status (overall)**				
Full Time	24	80.0	65	95.6
<Full Time	6	20.0	3	4.4
Did not disclose	0	0	0	0.0
**% of hours spent on academic work**				
100%	5	16.7	8	11.8
50%	19	63.3	3	4.4
<50%	6	20.0	57	83.8
Did not disclose	0	0	0	0.0
**Out of programme for research**				
No	19	63.3	55	80.9
Yes	5	16.7	3	4.4
Not applicable	6	20.0	10	14.7
**Ethnicity**				
Asian	2	6.7	11	16.2
Indian	2	6.7	3	4.4
Middle Eastern	1	3.3	2	2.9
Black	0	0	3	4.4
White Caucasian	24	80.0	47	69.1
Did not disclose	1	3.3	2	2.9
**Marital Status**				
Divorced	2	7.0	0	0.0

For peer review only

Long-term relationship (not married)	2	7.0	7	10.3
Married	23	77.0	56	82.4
Single	3	10.0	4	5.9
Did not disclose	0	0	1	1.5
**Sexuality**				
LGBTQIA+	1	3.3	4	5.9
Heterosexual	25	83.3	62	91.2
Did not disclose	4	13.3	2	2.9
**Disability**				
No	28	93.3	67	98.5
Yes	2	6.7	1	1.5
**Number of Children/Dependents**				
0	7	23.3	27	39.7
1	6	20.0	11	16.2
2	11	36.7	25	36.8
3	4	13.3	2	2.9
4	2	6.7	1	1.5
Did not disclose	0	0	2	2.9
**Pregnant**				
Yes	0	0	1	1.5
Did not disclose	1	3.3	7	10.3
No	29	96.7	60	88.2
**Current Clinical Academic Career Level**				
Doctoral Fellow/ PhD student	10	33.3	13	19.1
Academic Clinical Fellow	6	20.0	14	20.6
Academic Clinical Lecturer	6	20.0	14	20.6
Senior Clinical Lecturer and above (including Deans and Programme Directors)	6	20.0	27	39.7
Did not disclose	2	6.7	0	0.0
**Current grade within Clinical Role**				

Clinical Fellow	3	10.0	1	1.5
Registrar (Medical / Dental)	16	53.3	22	32.4
General Practitioner (Medical / Dental)	4	13.3	6	8.8
Medical / Dental consultant	6	20.0	30	44.1
Medical researcher	1	3.3	0	0.0
Did not disclose	0	0	1	1.5
**Location**				
East of England	1	3.3	2	2.9
Midlands	4	13.3	11	16.2
North East England & Yorkshire	11	36.7	26	38.2
North West of England	2	6.7	4	5.9
South East of England	6	20	16	23.5
South England	4	13.3	2	2.9
Wales	2	6.7	1	1.5
Scotland	0	0	5	7.4
Did not disclose	0	0	1	1.5
**Place primary health qualification awarded**				
UK	30	100	64	94.1
IMG	0	0	2	2.9
EEU	0	0	1	1.5
Did not disclose	0	0	1	1.5
**Total Number of Diary Entries**	134		N/A	
**Number of Written Entries**	26		N/A	

For peer review only

Themes

The analysis revealed four major themes, each with subthemes (Table 2), relating to the initial impact of COVID-19 on clinical academic careers; (1) opportunities, (2) barriers, (3) personal characteristics and social identity and (4) fears and uncertainty. Intersectionality and the differential impact of ethnicity and gender on the experiences were noted across all themes.

Due to the extensive dataset, only selected themes are presented in continuous prose; these have been chosen due to their pertinence to the research.

The analysis found the COVID-19 experiences of medical and dental clinical academics relatively similar, differences were noted when protected characteristics such as gender and ethnicity were considered.

Table 2: Themes and subthemes from audio diary entries

For peer review only

<table>
<tr><th>Opportunities</th><th>Barriers</th><th>Personal characteristics and social identity</th><th>Fears and Uncertainty</th></tr>
<tr><td colspan="4">⇐⇐⇐ Intersectionality ⇒⇒⇒
The impact of gender, ethnicity and associated intersectional identities were pertinent across all themes</td></tr>
<tr><td>New emerging opportunities
• New research avenues
• New collaborations
• COVID offers a new lens through which to think about existing research
• Increased capacity & opportunity to write</td><td rowspan="2">Lockdown
• Working at home with children
• Work life balance
• Parenting responsibilities
• Positive discrimination
• Gender roles
• Impact on physical & mental health from working at home
• Difficulty maintaining momentum
• Decline in academic outputs
• Isolation
• International work difficult
• Physical barriers impacting on work (e.g PPE, remote consultations)
• Demands from teaching responsibilities
• Loss of resource</td><td>Being a clinical academic
• Increased perceived importance of clinical academics
• Sitting in neither camp</td><td>Returning to the frontline
• Responding to the call to arms
• Contracting COVID-19
• BAME ethnicities increased susceptibility
• Anxiety
• Appropriateness of skillset for return to clinical duty
• Inability in short-term to conduct research
• Impact on long-term research career</td></tr>
<tr><td>Support
• Funder support (extensions & network)
• Supportive networks (local, national, international)
• Provision of childcare for keyworkers
• Camaraderie</td><td>Protected characteristics
• BAME
• Maternal status
• Gender stereotypes
• Intersectional identities</td><td>Misconceptions and absence information
• Funding
• Extensions
• Lockdown restrictions being lifted
• Fiscal impact and predicted recession
• Portfolio development due to loss of research capacity
• Future impact of COVID
• Uncertain job market</td></tr>
</table>

For peer review only

**Maintenance of normality**<br>• Job security<br>• Maintenance of structure<br>• Workload maintained	**Demand from teaching responsibilities**<br>• Switch to online teaching<br>• Lack of support for teaching activity<br>• Teaching is devalued	
**Technological advances supporting remote working**<br>• Maintenance of networks<br>• Inclusive approach to working	**Loss of resource**<br>• Loss of outputs<br>• Fiscal barriers<br>• Loss of research team & associated HR issues<br>• Decrease in academic capacity due to clinical workload<br>• Lack of research supervision<br>• Practical barriers (e.g. data storage)<br>• Absence of pastoral support	

For peer review only

<u>Theme 1: Opportunities and Enablers</u>

The pandemic presented many opportunities and enablers. These spanned academic and clinical work as well as participants' personal lives. Subthemes broadly fell into three categories; 1) new opportunities for research, 2) support, 3) maintenance of normality and 4) technological advances supporting remote working.

New Emerging Opportunities

Participants recognised the immediate opportunity to explore new avenues of research related to the pandemic.

> "*So I've been asked to, and volunteered to, be part of the Research Ethics Committee Coronavirus Response which means that every week now I've had twenty-four hour turnaround for a coronavirus study of some sort or another … I have been involved with developing two national and one international coronavirus studies over this time…an enormously exciting but terrifying roll out of research…*" [Male Medic - diary]

> "*…there might be a positive if you were, you know, an Oxford University clinician looking at vaccine so you'd obviously you get a lot of funding and, you know, good opportunity to, to research Covid based things… I suppose if you've never done a clinical trial before and you were a clinical academic, getting involved in some of the trials that they're doing at the moment, like the Recovery Trial, that could be a positive for you if you're in that particular situation…" [*Female Medic – interview*]*

Associated with this was the need for rapid research dissemination, providing clinical academics with a chance to develop their portfolio. This resulted in the forging of new research collaborations supported by the quick turnaround of ethical approval in order to roll out impactful research. More senior clinical academics reported opportunities for career development such as being invited to serve on Research Ethics Committees. In addition to the new research territory, COVID-19 presented a new lens through which participants were able to think about their existing research.

For some, salient amongst the opportunities was the free time which was created during the pandemic, particularly due to the absence of social commitments and commuting. Some clinical academics found this spare time to be an opportunity to focus on academic research activities and the production of impactful research. Others felt this was as an opportunity to focus on clinical work, as well as family and household commitments.

> **"***I guess it will give me a chance to focus on, you know, having a bit more time at home with family and, and just doing clinical work, which I think, you know, is what I wanted to focus on for the moment.*" [Female Medic – Audio Diary]

Support

Participants presented examples of the positive impact of support networks locally, nationally and internationally. The abrupt emergence of COVID and subsequent lockdown meant that many clinical academics halted their academic work and returned to clinical practice. This created an element of uncertainty, and in some cases anxiety about completing their academic work. However, a widely circulated statement

For peer review only

from the UK's main funding bodies reassuring clinical academics that they would support the extension of research post-COVID was well received.

> "*The [funder] have said that they will support extensions if they're needed so I do have that to kind of fall back on.*" [Female Medic – Audio Diary]

An immense amount of support was provided by academic organizations at the beginning of the pandemic. Support from supervisors, departments and funding bodies was identified as enabling and empowering for participants. For example, the provision of childcare nurseries, enabling clinical academics to return to full-time clinical practice when required.

Maintenance of Normality

'Maintaining the status quo' during the pandemic was an enabler for many. From the beginning of the pandemic many professions have seen large proportions of redundancy. However, this has not been the case for many of the clinical academics that participated in this study. Clinical academics who have had the flexibility of undertaking either clinical or academic work during the pandemic, were grateful and felt generally lucky to have a job during COVID and one they could go back to post-COVID. Opportunities that strengthened the resolve of participants included the option to flex hours between roles. Specifically, it was also noted that going back to clinical practice full-time was perceived as advantageous due to more structure compared to academic work. Additionally, the retention of social interaction, structure and workload was seen as an opportunity.

> "*In these times of COVID-19 some people have kind of lost their structure and workload, so I feel grateful that hasn't happened to me.*" [Male Medic – Audio Diary]

Technological advances supporting remote working

Participants advocated the use of video-conferencing platforms, citing them as enablers to maintain networks and collaborations during the pandemic as well as the inclusivity afforded by digital spaces.

> *"…my colleagues have become much more able to work online using Microsoft Teams and there have been more meetings recorded and more webinars which meant that as a part-time person I've been able to attend some of the things that previously I used to enquire and they used to say that this is not recorded and it is not being broadcast, so that has been excellent." [Female Medic – Interview]*

Theme 2: Barriers

There were multifaceted barriers related to the direct consequences of the immediate impact, reactive and mid-term barriers, and longer-term implications. Three subthemes were identified; (1) lockdown, (2) demand of teaching responsibilities and, (3) loss of resource.

Lockdown

For peer review only

Most of the barriers described by participants related to the negative consequences of lockdown. A barrier evidenced by particularly emotive data was that of working at home. This entailed the need to balance the multiple commitments related to childcare, housekeeping, and productivity. There were far reaching consequences of the ways in which working from home impacted on participants' roles. Childcare was not seen as compatible with a clinical academic role. Both clinical (e.g. telephone clinics and clinical administration) and academic work was often taking place at home, leading to feelings of isolation.

> "*I've spent a lot of time doing very intensive blocks of clinical work and very intensive blocks of research, research policy into practice work…I realised it's a Thursday today, I realised I had worked for three weeks solid and I'd not had a full day off in that time and most of those days were ten or twelve hour days…and I have now worked a further eleven days, with the last four days being fourteen hour days, without a break*". [Male Medic- Interview]

Mental and physical health outcomes were described as a consequence of COVID, particularly due to the increased workload.

> "*Generally, the COVID experience has been exhausting on many levels; emotionally from the worry about the number of people who will die, worry about being redeployed, worry for my children's wellbeing.*" [Male Medic-Interview]

> *"My anxiety is elevated due to being Asian, will I get sick and lose time for research?"* [Female Medic-Interview]

Concerns arose over how to effectively manage competing time demands. Some also spoke about a loss of 'downtime' and feeling 'stuck in limbo'. The shift in work-time meant that participants had less time for recreational activities which impacted on wellbeing.

Both males and females were impacted by the move to home working and balancing childcare with full-time work. The time pressures were apparent in many situations where participants struggled to navigate competing demands. There were also concerns over positive discrimination in how gender roles were recognised, with a particular focus on senior positions.

> "*… the Pro Vice Chancellor had sent an email saying how exciting it was that there's a female President at the Medical School, a female Dean, a female Pro Vice Chancellor of Medicine and a female Vice Chancellor at University, and that made me reflect what would happen if I sent a similar email delighting the fact that there's males in all of those roles. I imagine there'd be some disdain… they're powerful and take revenge…I don't think that's appropriate.*" [Male Medic – Audio Diary]

Demands from teaching responsibilities

The analysis identified the negative impact of having to rapidly produce teaching and assessment materials for online delivery, frequently coupled with a lack of support

and appreciation, as well as the time consumed. In many cases, this was in addition to clinical responsibilities.

> "*As clinical academics we are scavenging time in between the clinical parts to crack on and do our academic work*." [Male Medic – Audio Diary]

There was worry over a future lack of recognition within portfolios for teaching activities undertaken, and a frustration over negotiating the various new technologies required to deliver teaching.

Loss of resource

The impact of COVID has been significant for participants and extends from fiscal and time to human resource issues. There were concerns over the loss of time in relation to academic outputs and not fulfilling previously arranged activities. A lack of overall supervision was noted, including much needed pastoral support.

> "*There is an absence of pastoral support. Supervision is different, it's about skills and development not always about just how you feel. Sometimes you need a shoulder to cry on*". [Female Medic – Audio Diary]

Given the measures in the academic field where outputs are often used as a productivity marker, the participants were unsure how they could compensate loss of time and the ways in which their activities could be demonstrated and valued.

Tangible barriers were highlighted in the monetary implications for how loss of time could be compensated. Participants made calls for funders to extend time and funding for projects that otherwise may have been completed on time.

> "*It's bloody terrifying when it comes to applying for new stuff though because the deadlines haven't really moved much and there's no head space and there's no time to get the stuff really done properly and that's really scary but we've got to get the clinical work done first. It's the only way*." [Male Medic – Audio Diary]

Many universities initiated a recruitment freeze which meant in some cases that methodological expertise was lost. As clinical academics are often on a set educational employment path for a discrete time period this created disruption to long-term career plans.

<u>Theme 3: Personal Characteristics and Social Identity</u>

A key theme revealed was the impact of COVID-19 on participants' personal characteristics and social identity. This theme specifically considers how individuals perceived themselves as being clinical academics and the issues associated with the label 'clinical academic'.

Ambivalence and identity of a clinical academic

Some participants were conflicted about their identity as clinical academics and often experienced 'imposter syndrome'. Imposter syndrome, as described by the participants, refers to a feeling that they were not adequately qualified to

be called clinical academics. Others expressed pride in being classed as clinical academics, however, they expressed that their identity as clinical academics was misunderstood by clinical and academic colleagues. Participants described problems experienced with negotiating both clinical and academic identities during the pandemic.

> "*Trying to tussle where you fit between university and clinical world at the moment is, is also hard*." [Male Medic– Audio Diary]

Participants highlighted that in their working life they do not sit neatly in either category, which makes it difficult to identify as either an academic and/or a clinician. This feeling has been intensified by the pandemic. Most professionals have an educational background where they have developed peer support and networks allowing for guidance, but for clinical academics, their varied pathways means they often lack this important support network.

Protected characteristics

Protected characteristics of clinical academics, particularly the BAME community who have been more widely affected by COVID-19, was a key subtheme identified. Increased anxiety was highlighted and participants who were from BAME backgrounds expressed fear about returning to clinical work. Muslim clinical academics also expressed the difficulty they faced due to Ramadan taking place during the already difficult time. The focus was largely on physical wellbeing due to heavy workload alongside being unable to eat or drink.

Gendered differences were evident throughout this study, with female participants specifically expressing the struggles of maternal identity, highlighting problems faced with childcare responsibilities as well as having to share these with their partners alongside work commitments.

> *"I'm not formally planning to carry on with academia and that's a decision that's kind of come about because of, you know, childcare responsibilities and I just felt that I was going to be stretched too thin if I was trying to be a mum and be a GP and be an academic as well.*" [Female Medic– Audio Diary]

Female participants felt that due to gender stereotypes from partners and work colleagues they were having to take a leading responsibility in childcare during this time.

> "*I think that COVID has been a disaster for feminism...the disagreements that I've had with [husband] have been over feeling like a 1950s housewife suddenly then to suddenly home schooling children and trying to stay sane, stay safe, keep everybody okay, whereas he still is doing exactly what he does, work...*" [Female Medic– Audio Diary]

Whilst participants noted intersectionality, their narratives were mostly differentiating their experiences due to gender. Women explicitly stated that they became less tolerant of the gender issues raised as time in lockdown progressed.

For peer review only

Women reported the emotional turmoil associated with being primary caregivers.

> *"Yet another day of working from home while helping with young kids who are upset at the disruption to their lives. The most difficult thing is trying to calm my children's' worry over COVID."* [Female Dentist– Audio Diary]

Increased perceived importance of clinical academics

Despite the tensions that have been caused by the pandemic, some noted that the work and identity of clinical academics have actually been strengthened. COVID has required rapid research to be undertaken and clinical academics are perfectly suited to this opportunity. The fact that qualified clinical academics have a foot in both research and clinical practice has facilitated a renewed appreciation of their identity during this pandemic.

Theme 4: Future fears and uncertainty

This theme related to numerous unanswered questions of participants including their fears and anxieties. Returning to the clinical environment was one major cause of anxiety, as well as the future of their research and funding.

Returning to the frontline

Discourse regarding a full return to clinical practice utilised many analogies to warfare. One source of internal conflict for participants was their inability to respond positively to the 'call to arms', particularly where they felt they had deskilled in some areas due to their focus on academia in recent years. Participants described requests to abandon research and assume clinical roles. Added to this anxiety was an inability to manage workload and family life.

> "*Well all of the academic trainees have been pulled on to the full-time clinical work. As clinical academics we are scavenging time in between the clinical parts to crack on and do our academic work and certainly emotionally I'm finding it enormous strain, mainly because the clinical work itself has not so much increased in the intensity but the massive decrease in staff numbers, partly because of illness but a lot because if isolation and shielding requirements that have gone on, has hugely increased the amount of work that all of us that are at work are doing.*" [Male Medic– Audio Diary]

Additionally, the fear of contracting COVID-19 was expressed, with the majority of participants having to return to full clinical practice. CAs who expressed fear of contracting COVID-19 were those with predisposing factors and BAME backgrounds. Additionally, knowing someone who had died on the frontlines increased clinical academics' perception of their own risk.

Misconceptions and concerns about the future

From the onset of the pandemic, participants have reported concerns over their progression and future careers. Participants also aired concerns about the lockdown restrictions being lifted prematurely, stating that this could have further negative implications on their clinical and academic work. Uncertainty around COVID-19 has caused misconceptions and subsequently resulted in the absence of

For peer review only

information. Amongst participants, there is a general fear and uncertainty about the fiscal impact on research funding and the future clinical academia.

> "*Unfortunately my grant holders haven't sort of committed themselves to being able to extend my funding due to various reasons, so yeah, so there's uncertainty with that as well.*" [Female Medic– Audio Diary]

Issues such as pay cuts and a loss of staffing resources within academic departments caused further anxieties.

> *"The news on pay cuts is a bitter pill to swallow when working in a department where teaching commitments have increased, I have to say. Clinicians may decide to switch to clinical only in some cases."* [Male Medic– Audio Diary]

These anxieties have been further intensified following a lack of professional and social networking opportunities, especially as the pandemic intensified and lockdowns progressed.

Box 1: Case examples (CE) of the initial impact of COVID-19 on participants with intersecting identities (pseudonyms utilised).

Box 1: Case studies of the impact of COVID-19 (pseudonyms utilised).

Case study 1: Asian, Mother, Muslim, Medical Registrar/Doctoral Fellow
Zainab, a Medical Registrar in Neurology and Doctoral Fellow is a married, Asian, Muslim, mother of three young children. She is 'proudly intersectional'. Zainab works full time and spends about 90% of her time on her academic work. She has recently put her details down to be called to the frontlines and is currently on the standby list. As an Asian doctor, living and working in a city, she feels particularly vulnerable and has fears about her risks of contracting COVID. Her anxieties regarding the virus increased over time as research linked BAME ethnicity to outcomes. She knows of a doctor who died on the front line, they were a relative of one of her closest friends. Although she realizes that susceptibility to the disease is multifactorial, she can see that people from minority ethnic groups are disproportionately affected. According to her, "*it's not known why [there's a disproportionately higher number] I think it is multifactorial whether there's underlying kind of health vulnerabilities that are more prevalent in certain ethnic groups that make them more vulnerable or whether there's more complex issues to that.*" Despite the negative implications that the COVID pandemic and lockdown have brought, she finds a great deal of positives from the supportive academic institution.

Case study 2: White, Mother, Clinical Lecturer
Rose, a Medical Registrar and Academic Clinical Lecturer, has been working at home where she is married to a clinical professor with two children. Rose works part-time. She is struggling with the balance of family and work commitments, stating that lockdown has reinforced sexist gender roles. Rose feels that her research has taken a backseat while her husband's work has been prioritised, "*I genuinely think he thinks his work is more important, I genuinely think that's what he believes.*" She reports a decline in mental health due to the pressures of lockdown and family dynamics, "*I think that COVID has been a disaster for feminism...the disagreements that I've had with [husband] have been over feeling like a 1950s housewife suddenly, then to suddenly have to provide home schooling children and trying to stay sane, stay safe, keep everybody okay, whereas he still is doing exactly what he does [work]...so I think that, that, that's my big worry is that actually COVID's been a step back.*" Rose's anxiety about her research profile increased the longer lockdown restrictions were in place.

Discussion

This study presents a unique, qualitative data-driven reflection of the national clinical academic landscape during nine months of the COVID-19 pandemic. The in-depth interview data are supported by real time audio diaries. This multi-faceted approach has facilitated access to “periods of change and flux” particularly during the pandemic, and allowed the research team to capture the impact of COVID-19 in real-time which facilitated accurate data capture [21, 22]. The audio diaries are also advantageous owing to their ease of completion, which typically results in lower levels of attrition. [22] Participants were also permitted to submit written entries, but this was not deemed to be a limitation, especially as discourse analysis was not planned. Permitting flexibility also prevents diary entries from becoming formulaic [22]. Whilst the authors recognize that there is often a self-selection bias associated with studies of this nature, participants were felt to be balanced in their presentation of barriers and enablers [23].

Clinical academics were impacted by COVID-19 in multiple ways leading to a wide range of barriers, challenges and opportunities underpinned by their personal and professional identities. The selflessness and dedication of the study participants to contribute to the clinical response to the COVID-19 pandemic is evident in the data. Clinical academics returning to the NHS during the pandemic, had added value with respect to their research experience[24].

Our findings identified the numerous perceived barriers to continuing academic activity within the family, academic and clinical contexts. What is clear is that pre-existing barriers to academic activity have become magnified during the COVID-19 pandemic. Although such barriers are not insurmountable, they have proved and continue to prove stressful for the participants. The impact on their future career plans is already evident. The restrictions on face-to-face contact, international travel, uncertainties over clinical and academic training and funding extensions, home working, and, in many cases, redeployment to frontline clinical duties have all impacted on academic activities. Tension was evident when academic trainees who switched to full time clinical work and sacrificed their academic time described how they felt disadvantaged in comparison to trainees who have been able to maintain research.

Female participants described barriers that related to their gender, as well as for many, their maternal status, with such biases having been previously well documented in the literature [18, 20, 25-27]. Our findings in the themes (2 and 3) and case studies, suggest that the pandemic and resultant lockdown has, in many cases, exacerbated the discrimination faced by many women in clinical medical and dental academia. A survey of dental clinical academics in 2018, found that overall female representation in the academic grades in Dentistry is increasing - mirroring gender representation in dentistry as a whole [28]. Given our data, it is possible that the COVID-19 pandemic could have an even more significant negative impact on female Dental Clinical Academics going forward.

Theme three also identified a number of females in our sample who described feeling and being compelled to assume, to a greater degree than before the pandemic, gender stereotypical roles in the home. Specifically, when faced with

competing responsibilities females appeared to feel under more pressure than their male partners to allocate more time to childcare and household tasks. Indeed, there was a recurrent motif of narratives relating to the '1950s housewife' stereotype within the family context [29]. In this regard, our findings are consistent with media accounts that the lockdown has placed a disproportionate burden on females, who frequently report taking on the bulk of the childcare, domestic duties and home-based education of school-aged children [30, 31]. It has been postulated that many females are currently doing 'second and third shifts' with regard to housework and welfare after their first shift of childcare or paid work [29]. Prior to COVID-19, there was evidence that parental working determined that, on average, females spent two to three times as much time on care and housework compared to men [32]. Our findings suggest that these effects have become more pronounced as a result of the pandemic and are impacting significantly on female clinical academics. Our data may go some way towards explaining reported declines in publications by female academics [33].

The findings raise an alarm that prolonged periods of lockdown could reverse general trends towards a more equitable distribution of household labour [32]. More concerningly, it may substantially put back efforts to achieve a more gender representative body of clinical academics, characterised by gross under representation of female, explored by a recent systematic review [9]. This has been shown to not merely be a matter of social equality; the male dominance of science in general has served women's health poorly [34].

There are a number of steps that could be taken to mitigate the impact of the COVID-19 pandemic on clinical academia as a whole and the risk of reduced long-term clinical research capacity, especially the loss of female academics. Funders and employers could intentionally target support at those with caring responsibilities during lockdown and the following recovery period. As the pressing clinical need recedes then prioritising the return of female academics to their research activities may also offset the bias more generally experienced by female scientists both before and during the pandemic. Whilst virtual networks of collaborators have organically sprung up in places during the lockdown, more of these could be strategically formed to counter the isolation felt by many academics. This support should also extend to academics who are primarily clinical educators, and have historically felt marginalised and undervalued [35]. Indeed, some of the participants in the present study voiced perceptions of a lack of support and appreciation from both their academic and clinical employers, and colleagues. Both existing and trainee clinical academics reported a perception that neither non-academic clinical colleagues or non-clinical academics fully appreciated the competencies and the time demands of their academic roles. This perception has been reinforced by the COVID-19 pandemic. Going forward, enhanced collaboration and empathy between University and Clinical staff and employers could be targeted to remedy this.

As the UK navigates its immediate response to COVID-19, it is important to consider the potential implications for those whose careers are most vulnerable and require additional nurturing. There needs to be increased flexibility in career pathways and expectations, along with a review of funding and support offered. Participants requested flexibility with existing funding to make changes to study protocols and costings to enable them to pursue opportunistic COVID related research. Leaders in

For peer review only

academic institutions and funding bodies must be cognisant of the varying degrees to which clinical academics will have been able to mitigate for COVID-19 in their assessments of applications for funding and positions and must revise their expectations for dissemination and impact. Causal explanations of the issues faced during COVID-19 will be multifaceted and sensitive to personal context. Teaching commitments must be valued and accounted for, as well as physical and mental health. It has been shown that clinical academics create national wealth as well as health [36]. We are heading into an inevitable global financial recession, if not depression, likely to also be characterised by an increase in mental and physical health needs which will undoubtedly impact on the clinical academic workforce and funding.

The infrastructure for medical and dental clinical academia is well established, yet our data suggest that better support is required to develop future clinical academic roles and enhance the provision of evidence-based patient care in the post-COVID-19 era. Participants report substantiated concerns over access to funding and positions once the status quo is resumed.

Whatever interventions and strategies are implemented, heed needs to be taken to the findings of Laver et al., who reported that interventions within clinical academia that require those they are designed to support, so called ‘bottom-up’ approaches, to bear the brunt of the workload are less likely to succeed [37]. Their review advocates the use of ‘top-down’ approaches, led by change in practice at a management level. Finally, their stance was that ‘something is better than nothing’.

Our findings offer further insight into the debate surrounding access to and maintenance of clinical academic careers [38]. We add weight to the evidence surrounding gender disparity. The well documented decline in the clinical academic workforce, the subject of contemporary research, [9, 39] could continue if strategies to support clinical academics are not implemented in the aftermath of the COVID-19 pandemic. Ongoing commitment to supporting and developing clinical academics is required, acknowledging the implicit biases facing females and parents in reaching their potential, and those with other competing demands on their time. Of paramount importance is the need for greater recognition of the value and contribution made by clinical academics to both the clinical and academic workforces to which they contribute. The reports from a recent national study by Finn and colleagues presented a number of proposed interventions to support clinical academics in the immediate wake of the pandemic, in addition to recommendations for ensuring the long-term sustainability of the clinical academic pipeline. Headlines from qualitative data included the need for mentorship, flexibility and transparent processes, as well as recognition of the differential experiences of marginalised groups[10].

Our findings also emphasise the need for guidance for aspiring clinical academics. As one remarked, “I just need guidance on next steps. It is just so challenging to think about who might help me.” Furthermore, without urgent action COVID-19 has the potential to increase the gender divide seen within clinical academia. It is hoped that the narratives in this study initiate a dialog between clinical academics, funders and institutions to successfully navigate the clinical academic pathways moving forward.

Declaration

The manuscript is an honest, accurate, and transparent account of the study being reported. There are no omissions or inaccuracies. Participants have engaged in member checking of results and are briefed of findings.

Funding

This publication presents independent research funded by Cancer Research UK (Award Number: C71037/A29824), The Academy of Medical Sciences, Health Education England, The Medical Research Council, The National Institute for Health Research (NIHR) and The Wellcome Trust. The views expressed are those of the author(s) and not necessarily those of The Academy of Medical Sciences, Cancer Research UK, Health Education England, The Medical Research Council, The National Institute for Health Research (NIHR), The Wellcome Trust or the Department of Health and Social Care.

Acknowledgements

The authors wish to thank all the clinical academics who participated in this study. The authors acknowledge the work of the wider research team involved in this large-scale research project and thank them for their contributions. The authors also appreciate the insight of the project steering group at the Clinical Academic Training Forum. Thanks go to 'Women in Academic Medicine', and the British Medical Association for assistance in recruiting participants.

Data sharing

Deidentified transcripts would be made securely available to other researchers if a written case for access is made to the research team and permission is granted by the funders of the study

Author contributions

GF, PC, PT, MK, JM and JB conceived the study. GF, PC, PT, JB and JM obtained funding. ET, GF, JB, PC, ABK and AK collected data and conducted the analyses. GF, AK, JB, PT and PC recruited participants and drafted the manuscript. ABK and AK curated the data. All authors reviewed the final manuscript.

References

1. Brenner, A.M., *Supervising the tearful trainee: opportunities for professional growth and learning.* Academic psychiatry : the journal of the American Association of Directors of Psychiatric Residency Training and the Association for Academic Psychiatry, 2014. **38**(4): p. 497-501.
2. Hourston, G.J., *The impact of despecialisation and redeployment on surgical training in the midst of the COVID-19 pandemic.* International Journal of Surgery (London, England), 2020. **78**: p. 1.
3. Clinical Academic Training Forum, *Clinical Academic Training Forum: Subgroup to support returning clinical academics from the COVID-19 front line*. 2020.

For peer review only

4. Guarino, C.M. and V.M. Borden, *Faculty service loads and gender: Are women taking care of the academic family?* Research in Higher Education, 2017. **58**(6): p. 672-694.
5. Ranieri, V., et al., *Factors that influence career progression among postdoctoral clinical academics: a scoping review of the literature.* BMJ Open, 2016. **6**(10): p. e013523.
6. Flaherty, C. *No Room of One's Own: Early journal submission data suggest COVID-19 is tanking women's research productivity*. 2020 [cited 2020 21st April 2020]; Available from: https://www.insidehighered.com/news/2020/04/21/early-journal-submission-data-suggest-covid-19-tanking-womens-research-productivity.
7. Pinho-Gomes, A.-C., et al., *Where are the women? Gender inequalities in COVID-19 research authorship.* BMJ Global Health, 2020. **5**(7): p. e002922.
8. McEwan, J., *Engaging women in academic medicine in the UK: report of a workshop at the Association of Physicians Annual Meeting, 2 April 2009.* QJM: An International Journal of Medicine, 2010. **103**(9): p. 635-639.
9. Brown, J.V., et al., *From the sticky floor to the glass ceiling and everything in between: protocol for a systematic review of barriers and facilitators to clinical academic careers and interventions to address these, with a focus on gender inequality.* Systematic reviews, 2020. **9**(1): p. 26.
10. Finn GM, B.J., Evans C, Raine G, Stewart L, Uphoff E, Kehoe A, Buchanan J, Balogun-Katung A, Tiffin P, Taylor E, Crampton P, Morgan J., *From the sticky floor to the glass ceiling and everything in between: A systematic review and qualitative study focusing on gender inequalities in Clinical Academic careers. Final report. .* 2020.
11. Finn GM, B.J., Evans C, Raine G, Stewart L, Uphoff E, Kehoe A, Buchanan J, Balogun-Katung A, Tiffin P, Taylor E, Crampton P, Morgan J., *Inequalities in UK clinical academic careers: a systematic review and qualitative study. Short report. .* 2020.
12. Gordon, L., et al., *Multiple and multidimensional transitions from trainee to trained doctor: a qualitative longitudinal study in the UK.* BMJ open, 2017. **7**(11).
13. Braun, U.K., et al., *The utility of reflective writing after a palliative care experience: Can we assess medical students' professionalism?* Journal of Palliative Medicine, 2013. **16**(11): p. 1342-1349.
14. Braun, V. and V. Clarke, *Using thematic analysis in psychology.* Qualitative research in psychology, 2006. **3**(2): p. 77-101.
15. Kiger, M.E. and L. Varpio, *Thematic analysis of qualitative data: AMEE Guide No. 131.* Medical Teacher, 2020: p. 1-9.
16. Roberts, K., A. Dowell, and J.-B. Nie, *Attempting rigour and replicability in thematic analysis of qualitative research data; a case study of codebook development.* BMC medical research methodology, 2019. **19**(1): p. 66.
17. Braun, V. and V. Clarke, *Successful qualitative research: A practical guide for beginners*. 2013: sage.
18. Williams, J.C. and N. Segal, *Beyond the maternal wall: Relief for family caregivers who are discriminated against on the job.* Harv. Women's LJ, 2003. **26**: p. 77.
19. Brown, M.E., et al., *'Too male, too pale, too stale': a qualitative exploration of student experiences of gender bias within medical education.* BMJ open, 2020. **10**(8): p. e039092.
20. Williams, J.C., *Hitting the maternal wall.* Academe, 2004. **90**(6): p. 16.

For peer review only

21. Monrouxe, L.V., *Negotiating professional identities: dominant and contesting narratives in medical students' longitudinal audio diaries.* Current Narratives, 2009. **1**(1): p. 41-59.
22. Crozier, S.E. and C.M. Cassell, *Methodological considerations in the use of audio diaries in work psychology: Adding to the qualitative toolkit.* Journal of occupational and organizational psychology, 2016. **89**(2): p. 396-419.
23. Collier, D. and J. Mahoney, *Insights and pitfalls: Selection bias in qualitative research.* World Politics, 1996. **49**(1): p. 56-91.
24. Rimmer, A., *Covid-19: How clinical academic trainees came back to the wards en masse.* bmj, 2021. **373**.
25. Crosby, F.J., J.C. Williams, and M. Biernat, *The maternal wall.* Journal of Social Issues, 2004. **60**(4): p. 675-682.
26. Williams, J.C., *The glass ceiling and the maternal wall in academia.* New Directions for Higher Education, 2005. **2005**(130): p. 91-105.
27. Halley, M.C., et al., *Physician mothers' experience of workplace discrimination: a qualitative analysis.* BMJ, 2018. **363**: p. k4926.
28. Council, D.S., *Professorial roles for dentists fall 8.3% since 2015.* British Dental Journal, 2018. **225**(5).
29. Chung, H. *Return of the 1950s housewife? How to stop coronavirus lockdown reinforcing sexist gender roles*. 2020; 21st April 2020]. Available from: https://theconversation.com/return-of-the-1950s-housewife-how-to-stop-coronavirus-lockdown-reinforcing-sexist-gender-roles-134851.
30. Ferguson, D., *'I feel like a 1950s housewife': how lockdown has exposed the gender divide*, in *The Guardian*. 2020.
31. Alon, T.M., et al., *The impact of COVID-19 on gender equality*. 2020, National Bureau of Economic Research.
32. Wishart, R., et al., *Changing patterns in parental time use in the UK*. 2019.
33. Viglione, G., *Are women publishing less during the pandemic? Here's waht the data say*, in *Nature*. 2020. p. 365-366.
34. Burford, B., et al., *Professionalism education should reflect reality: findings from three health professions.* Medical Education, 2014. **48**(4): p. 361-374.
35. Kumar, K., C. Roberts, and J. Thistlethwaite, *Entering and navigating academic medicine: academic clinician-educators' experiences.* Medical education, 2011. **45**(5): p. 497-503.
36. Innovation, B. and G. Team, *Bioscience 2015: Improving National Health.* Increasing National Wealth, 2003.
37. Laver, K.E., et al., *A systematic review of interventions to support the careers of women in academic medicine and other disciplines.* BMJ open, 2018. **8**(3).
38. Rothwell, P.M., *Medical academia is failing patients and clinicians*. 2006, British Medical Journal Publishing Group.
39. Fitzpatrick, S., *A survey of staffing levels of medical clinical academics in UK medical schools as at 31 July 2011.* London: Medical Schools Council, 2012.

For peer review only

Appendix 1: Interview and example audio dairy prompts for participants during COVID-19

Example interview stems for participants during COVID-19

Semi-structured approach thus will be guided by participants.

1. **Introductions**

2. **Please describe your role and career path to date -** *both clinical and academic*

3. **What are your experiences of clinical academia?**

4. **What factors impacted upon your decision to pursue (or not pursue) a career in clinical academia?**
- *What factors make clinical academia an attractive career?*

5. **What has your experience with awards/ funding been like? (if any)**
- *Application process? (facilitators/barriers)*
- *Support through process*
- *Why did/didn't continue? (e.g. after PhD why didn't do lectureship?)*
- *If not applied, why not?*
- *Did this experience influence your choice in career pathway?*

6. **What barriers and enablers have you faced within clinical academia?**
- *What made you continue/not continue?*
- What stops people returning to clinical academia?
- How do you negotiate the 2 career paths?
- What goes wrong in institutions that funders can help with?
- What worked well/ less well with other funders you have experienced?
- Impact of the COVID-19 pandemic

7. **What could be done to improve equal representation within clinical academia?**

8. **Describe any interventions to promote access to clinical academia or reduce discrimination that you have experienced.**
- *What worked?*
- *Given your context, what would you recommend?*

9. **What existing or new interventions could help to reduce attrition in clinical academic careers?**

10. **How can organisations support trainees and clinical academics in their career decisions and academic pathways?**

Example audio dairy prompts for participants during COVID-19

Participants were able to reflect on all prompts any situation pertaining to Clinical Academia that they wished. Prompts were broad and participants were informed of the interest in protected characteristics.

How has COVID-19 impacted upon your work as a clinical academic?	What have you heard about funding (opportunities, threats, remit etc) post COVID-19?	Has COVID-19 had a detrimental impact on or changed your career plans?
How has the expectation of the impact of COVID-19 differed from the reality?	How have your protected characteristics impacted upon your experience of clinical academia during COVID-19?	BAME participants: Has the emerging data on the enhanced risk to the BAME workforce from COVID-19 had any impact on you?
Has COVID-19 had a detrimental impact on or changed your research?	What barriers and enablers have you faced during COVID-19?	Parents/ carers: How have your responsibilities impacted upon your ability to work?

For peer review only

BMJ Open

The COVID-19 pandemic: A problem for clinical academia, a disaster for women.

Journal:	*BMJ Open*
Manuscript ID	bmjopen-2021-057655.R1
Article Type:	Original research
Date Submitted by the Author:	11-Feb-2022
Complete List of Authors:	Finn, Gabrielle; The University of Manchester, Division of Medical Education, School of Medical Sciences, Faculty of Biology, Medicine and Health<br>Crampton, Paul; Hull York Medical School, Health Professions Education Unit<br>Buchanan , John; Barts and The London School of Medicine and Dentistry, Centre for Education and Innovation<br>Balogun, Abisola Olatokunbo; Hull York Medical School, Health Professions Education Unit<br>Tiffin, Paul; University of York, Health Sciences<br>Morgan, Jessica; University of York, Centre for Reviews and Dissemination; Leeds Teaching Hospitals NHS Trust, Department of Paediatric Oncology<br>Taylor , Ellie; Hull York Medical School, Health Professions Education Unit<br>Soto, Carmen; British Medical Association<br>Kehoe, Amelia; Hull York Medical School, Health Professions Education Unit
**Primary Subject Heading**:	Qualitative research
Secondary Subject Heading:	Medical education and training, Qualitative research, Research methods, Public health
Keywords:	COVID-19, QUALITATIVE RESEARCH, MEDICAL EDUCATION & TRAINING

SCHOLARONE™
Manuscripts

I, the Submitting Author has the right to grant and does grant on behalf of all authors of the Work (as defined in the below author licence), an exclusive licence and/or a non-exclusive licence for contributions from authors who are: i) UK Crown employees; ii) where BMJ has agreed a CC-BY licence shall apply, and/or iii) in accordance with the terms applicable for US Federal Government officers or employees acting as part of their official duties; on a worldwide, perpetual, irrevocable, royalty-free basis to BMJ Publishing Group Ltd ("BMJ") its licensees and where the relevant Journal is co-owned by BMJ to the co-owners of the Journal, to publish the Work in this journal and any other BMJ products and to exploit all rights, as set out in our licence.

The Submitting Author accepts and understands that any supply made under these terms is made by BMJ to the Submitting Author unless you are acting as an employee on behalf of your employer or a postgraduate student of an affiliated institution which is paying any applicable article publishing charge ("APC") for Open Access articles. Where the Submitting Author wishes to make the Work available on an Open Access basis (and intends to pay the relevant APC), the terms of reuse of such Open Access shall be governed by a Creative Commons licence – details of these licences and which Creative Commons licence will apply to this Work are set out in our licence referred to above.

Other than as permitted in any relevant BMJ Author's Self Archiving Policies, I confirm this Work has not been accepted for publication elsewhere, is not being considered for publication elsewhere and does not duplicate material already published. I confirm all authors consent to publication of this Work and authorise the granting of this licence.

For peer review only

The COVID-19 pandemic: A problem for clinical academia, a disaster for women.

*Gabrielle M Finn[1], Paul E S Crampton[2], John Buchanan[3], Abisola Balogun-Katung[2], Paul A. Tiffin[4], Jessica E Morgan[5,6], Ellie Taylor[2], Carmen Soto[7], Amelia Kehoe[2]

Affiliations

1. Division of Medical Education, School of Medical Sciences, Faculty of Biology, Medicine and Health, The University of Manchester, M13 9NT
2. Health Professions Education Unit, Hull York Medical School, University of York, York, UK, YO10 5DD
3. Queen Mary University London, Barts Health NHS Trust
4. Mental Health and Addiction Research Group, Department of Health Sciences, University of York, York, UK, YO10 5DD
5. Centre for Reviews and Dissemination, University of York, York, UK, YO10 5DD
6. Department of Paediatric Oncology, Leeds Children's Hospital, Leeds, UK, LS1 3EX
7. British Medical Association, London, WC1H 9JP

Corresponding author:
Professor Gabrielle M. Finn
Division of Medical Education, School of Medical Sciences, Faculty of Biology, Medicine and Health, The University of Manchester, M13 9NT
gabrielle.finn@manchester.ac.uk]
Twitter: @gabs_finn

Word count: 5712

For peer review only

The COVID-19 pandemic: A disaster for clinical academia. Where are all the women?

Abstract

Objective: To investigate the impact of the COVID-19 pandemic on the research activity and working experience of clinical academics (CAs), with a focus on gender and ethnicity.

Design: Interviews and audio/written diary study.

Setting: United Kingdom (UK) study within clinical academia

Participants: Purposive sample of 82 clinical academics working in medicine and dentistry across all career stages ranging from academic clinical fellows, and doctoral candidates to professors.

Methods: Qualitative semi-structured interviews (n=68) and audio diary data (n=30) collected over an eight-month period (January-September 2020), thematically analysed.

Results: Four major themes were identified in relation to the initial impact of COVID-19 on Clinical Academics; opportunities, barriers, personal characteristics and social identity and fears & uncertainty. COVID-19 presented opportunities for new avenues of research. Barriers included access to resources to conduct research and the increasing teaching demands. One of the most prominent sub-themes within 'personal characteristics' was that of the perceived negative impact of the pandemic on the work of female CAs. This was attributed to inequalities experienced in relation to childcare provision and research capacity. Participants described differential experiences based upon their gender and ethnicity, noting intersectional identities. Participants included 60 females and 30 males, 80% identified as white and 20% as Black, Asian or another minority (BAME).

Conclusions: Whilst there have been some positives afforded to CAs, particularly for new avenues of research, COVID-19 has negatively impacted workload, future career intentions, and mental health. BAME CAs were particularly fearful due to the differential impact on health. Our study elucidates the direct and systemic discrimination that creates barriers to women's career trajectories in clinical academia. A flexible, strategic response that supports CAs in resuming their training and research is required. Interventions are needed to mitigate the potential lasting impact on CA capacity from the pandemic, and the potential for the loss of women from this valuable workforce.

Article Summary

- Our findings suggest that the ongoing COVID-19 pandemic is leading to a continued decline in the progression of female clinical academics due to the reinforcement of gender stereotypes, particularly within the home.
- Support from institutions and funders in items of flexible working, valued teaching portfolios, protected research time and extensions could help

For peer review only

mitigate the impact of COVID-19 on clinical academics who are uniquely positioned to tackle key research problems during the pandemic.

Strengths and Limitations

- This study used both audio and written diary entries as well as in-depth interviews, which provided a more comprehensive picture while capturing the complexity of the experiences of clinical academics.
- The use of video conferencing and telephone interviews provided flexibility which allowed for more data to be collected, particularly at the start of the pandemic when face-to-face data collection methods would not have been feasible.
- Our findings are based on data from a UK sample, which may be perceived as limited. We balanced this out by having a large sample size.
- The research team consisted of clinical academics from various backgrounds at various stages of their careers who were able to fact check data collected.
- The research explored sensitive issues around experiences of clinical academics. The use of audio and written diary entries and in-depth interviews require self-reporting and therefore may have biased the findings.

For peer

Introduction

The workforce presence of clinical academics is vital to healthcare to ensure that scientific knowledge underpins advances in medical education, translational research and patient outcomes. Their low number and lack of senior pipeline progression is alarming, particularly so for under-represented groups including women and ethnic minorities.[1] The COVID-19 pandemic has further exacerbated the situation through rapid redeployment, burnout, reinforcement of gender and racial stereotypes, and financial inequity.[2]

Over 1,500 academic trainees in England were estimated to be redeployed to clinical work during the COVID-19 pandemic[3]. This equates to more than 90% of those on the Integrated Academic Training (IAT) pathway.[3] Following the closure of high-risk aerosol-generating dental services, some junior hospital dentists and dental clinical academics were also redeployed to provide support care for hospital services.[4] The potential impact on trainees' clinical experience has been recognised.[5] In terms of formally monitoring and permitting advancement in postgraduate training, 'no fault' Annual Review of Competence Progression (ARCP) outcomes (10.1 and 10.2) have been introduced where acquisition of competencies has been delayed owing to COVID-19[6]. Meanwhile, there has been a research paradox whereby some research institutions have closed, while others have adapted and embraced COVID specific research [7]. Further issues highlight the consumption of academic resources that may prove ineffective or wasteful owing to haste, rapid redesign of online teaching materials, and inadequate infrastructure and design.[8] With the current prioritization of COVID-19 studies, new clinical trials have been paused [9] with detrimental impacts on clinical academics working on non-COVID research.

Prior to the pandemic, it was recognised that females in medicine tended to take on more ‘service work’ and had less protected time for research than their male counterparts.[4] In addition to low representation of minority groups, the lack of role models provides a further barrier. [5] Collectively, gender and racial stereotypes, childcare and home-working all put further pressure on an already stretched workforce, with women now publishing less.[6, 7] Losing further women and minority groups from this fragile workforce could run the risk of long-term reduced diversity in medical education, less female-relevant clinical research and ultimately have a negative impact on patient outcomes, in addition to having less women visible in senior positions [8, 9].

We sought to explore discrimination based upon protected characteristics in the clinical academic workforce, at various stages of the pipeline during the pandemic. We aimed to understand the barriers, enablers and interventions to facilitate clinical academic careers, thereby to develop key recommendations to support and reduce attrition of these exceptionally talented individuals. We were particularly interested in the experiences of women and clinical academics from different ethnic groups.

Methods

Ethics

Ethical approval for this study was obtained from the Hull York Medical School Ethics Committee (ref: 19 32).

Context

Prior to the COVID-19 pandemic, the authors were engaged in a multi-funder research project exploring barriers and facilitators to a career in UK clinical academia[10, 11]. As data were iteratively analysed, the impact of COVID-19 on clinical academia progressively shaped the experiences of the participants and relevant themes emerged. In order to assess the impact of COVID-19, the data collected after the first confirmed case within the UK (29th January 2020) until the end of September 2020 were isolated for analysis. This period included the first period of significant national restrictions (“lockdown”) from 26th March to a phased reopening in June.

This study was conducted within a social constructivism orientation, additionally informed by the multiple principles of feminist theory. Feminist theory holds a constructivist ontology.

Patient and Public Involvement

The study had a steering group which included Patient and Public representation, provided by Health Watch

Study design and recruitment

Qualitative interviews and longitudinal qualitative audio and written diary entries were utilised. Purposive sampling was undertaken, using social media, funder mailing lists and snowballing. Inclusion criteria specified participants were a doctor or dentist,

For peer review only - http://bmjopen.bmj.com/site/about/guidelines.xhtml

based within the UK and working as or towards becoming a clinical academic. For the purposes of the study, a 'clinical academic' was defined by individuals themselves and how they perceived their roles. Recruitment was through various avenues; a website, emails sent on behalf of the research team by stakeholder organisations; social media recruitment; and 'snowballing'.

Data collection

Data were collected using (1) audio and written diary entries, and (2) semi-structured interviews. Diaries enabled participants to discuss their views on a wide range of factors related to clinical academic careers, and latterly their experiences of working during COVID-19. The diary method enabled researchers to collect 'novel' and timely data during the transition to lockdown phase, a critical moment in healthcare. Many diaries were recorded immediately after events, such as discriminatory remarks being made in a workplace. Participants were invited to submit audio diary entries (MP3 files) via email or attached to an encrypted message. Submissions were sent to a professional account linked to the project following best practice.[12] In order to promote engagement, participants were also permitted to write their diary entry.[12] Participants were able to submit diaries as frequently as they wished, each acknowledged by the researchers to maintain rapport and engagement. Participants were issued with a guide to follow which was informed by a concurrent systematic review.[9] The guide suggested frequencies of entries, instructions for submission and indicative content (Appendix 1). Reminder emails were sent monthly. Interviews were conducted via video conferencing or telephone with the use of a topic guide. Audio diary files and interviews were recorded and transcribed verbatim. All participants were given an electronic information sheet and consent form prior to the recording. Interviews were conducted by GF, PC, AK, JAG and ABK.

Data analysis

Data were thematically analysed [13, 14]. Coding was undertaken by GF, PC, AK, JAG, ABK and ET. Thematic analysis was chosen because it is an appropriate method for seeking to understand experiences, thoughts, or behaviours across a dataset [15]. The authors read the data in its entirety and developed a codebook as a tool to assist in the analysis of the large dataset [16]. The six-step process of thematic analysis was followed: (1) data familiarisation, (2) generating initial codes, (3) searching for themes, (4) reviewing themes, (5) defining and naming themes, and (6) producing the report.[14, 17] Both inductive and deductive approaches were taken, with deductive analysis based on existing theory including maternal wall bias, feminist theory and intersectionality.[18-20] Authors engaged in a process of negotiation to refine codes and themes, before utilising member checking with a subset of participants. Authors were reflexive, recording reflexive journals and acknowledging their biases and presuppositions. The research team consisted of clinicians and non-clinicians, clinical academics at varying stages, expert qualitative researchers to novices, females and males, and a mix of ethnicities. Since the study data were confined to a limited collection period during the pandemic, it was felt that this would not be of sufficient duration for meaningful temporal analysis. Instead researchers interpreted the data as being snapshots and in the moment reflections relating to the impact of the pandemic. In order to bring participants' experiences over time, cross-sectional themes together and demonstrate intersectionality case studies from two participants are provided (Box 1).

For peer review only

Results

Demographics

Eighty-two individuals participated, 30 provided audio diary data, 68 were interviewed, while 16 provided both. Table 1 provides an overview of the descriptive statistics of the participant sample and the data collected. The research team received 134 diary entries, with a mean of 5 entries per participant.

TABLE 1: Demographic breakdown of participants

	Audio Diaries		Interviews	
**Profession**	**Total (n=30)**	**%**	**Total (n=68)**	**%**
Medicine	24	80.0	57	83.8
Dentistry	6	20.0	11	16.2
**Mean Age**	39		40	
**Age Range**	27 - 74		28 - 51	
**Gender**				
Male	10	33.3	28	41.2
Female	20	66.6	40	58.8
**Predominant Clinical Work Area**				
Primary	7	23.3	19	27.9
Secondary	11	36.7	30	44.1
Tertiary	12	40.0	17	25.0
Did not disclose	0	0	1	1.5
**Employment Status (overall)**				
Full Time	24	80.0	65	95.6
<Full Time	6	20.0	3	4.4
Did not disclose	0	0	0	0.0
**% of hours spent on academic work**				
100%	5	16.7	8	11.8
50%	19	63.3	3	4.4
<50%	6	20.0	57	83.8
Did not disclose	0	0	0	0.0
**Out of programme for research**				
No	19	63.3	55	80.9
Yes	5	16.7	3	4.4
Not applicable	6	20.0	10	14.7
**Ethnicity**				
Asian	2	6.7	11	16.2
Indian	2	6.7	3	4.4
Middle Eastern	1	3.3	2	2.9
Black	0	0	3	4.4

For peer review only

White Caucasian	24	80.0	47	69.1
Did not disclose	1	3.3	2	2.9
**Marital Status**				
Divorced	2	7.0	0	0.0
Long-term relationship (not married)	2	7.0	7	10.3
Married	23	77.0	56	82.4
Single	3	10.0	4	5.9
Did not disclose	0	0	1	1.5
**Sexuality**				
LGBTQIA+	1	3.3	4	5.9
Heterosexual	25	83.3	62	91.2
Did not disclose	4	13.3	2	2.9
**Disability**				
No	28	93.3	67	98.5
Yes	2	6.7	1	1.5
**Number of Children/Dependents**				
0	7	23.3	27	39.7
1	6	20.0	11	16.2
2	11	36.7	25	36.8
3	4	13.3	2	2.9
4	2	6.7	1	1.5
Did not disclose	0	0	2	2.9
**Pregnant**				
Yes	0	0	1	1.5
Did not disclose	1	3.3	7	10.3
No	29	96.7	60	88.2
**Current Clinical Academic Career Level**				
Doctoral Fellow/ PhD student	10	33.3	13	19.1
Academic Clinical Fellow	6	20.0	14	20.6
Academic Clinical Lecturer	6	20.0	14	20.6
Senior Clinical Lecturer and above (including	6	20.0	27	39.7

For peer review only

Deans and Programme Directors)				
Did not disclose	2	6.7	0	0.0
**Current grade within Clinical Role**				
Clinical Fellow	3	10.0	1	1.5
Registrar (Medical / Dental)	16	53.3	22	32.4
General Practitioner (Medical / Dental)	4	13.3	6	8.8
Medical / Dental consultant	6	20.0	30	44.1
Medical researcher	1	3.3	0	0.0
Did not disclose	0	0	1	1.5
**Location**				
East of England	1	3.3	2	2.9
Midlands	4	13.3	11	16.2
North East England & Yorkshire	11	36.7	26	38.2
North West of England	2	6.7	4	5.9
South East of England	6	20	16	23.5
South England	4	13.3	2	2.9
Wales	2	6.7	1	1.5
Scotland	0	0	5	7.4
Did not disclose	0	0	1	1.5
**Place primary health qualification awarded**				
UK	30	100	64	94.1
IMG	0	0	2	2.9
EEU	0	0	1	1.5
Did not disclose	0	0	1	1.5
**Total Number of Diary Entries**	134		N/A	

For peer review only

**Number of Written Entries**	26	N/A

Themes
The analysis revealed four major themes, each with subthemes (Table 2), relating to the initial impact of COVID-19 on clinical academic careers; (1) opportunities, (2) barriers, (3) personal characteristics and social identity and (4) fears and uncertainty. Intersectionality and the differential impact of ethnicity and gender on the experiences were noted across all themes.

Due to the extensive dataset, only selected themes are presented in continuous prose; these have been chosen due to their pertinence to the research.
The analysis found the COVID-19 experiences of medical and dental clinical academics relatively similar, differences were noted when protected characteristics such as gender and ethnicity were considered.

Table 2: Themes and subthemes from audio diary entries

For peer review only

<table>
<tr><th>Opportunities</th><th>Barriers</th><th>Personal characteristics and social identity</th><th>Fears and Uncertainty</th></tr>
<tr><td colspan="4">⇐ ⇐ ⇐ Intersectionality ⇒ ⇒ ⇒
The impact of gender, ethnicity and associated intersectional identities were pertinent across all themes</td></tr>
<tr><td>New emerging opportunities
• New research avenues
• New collaborations
• COVID offers a new lens through which to think about existing research
• Increased capacity & opportunity to write</td><td rowspan="2">Lockdown
• Working at home with children
• Work life balance
• Parenting responsibilities
• Positive discrimination
• Gender roles
• Impact on physical & mental health from working at home
• Difficulty maintaining momentum
• Decline in academic outputs
• Isolation
• International work difficult
• Physical barriers impacting on work (e.g PPE, remote consultations)
• Demands from teaching responsibilities
• Loss of resource</td><td>Being a clinical academic
• Increased perceived importance of clinical academics
• Sitting in neither camp</td><td>Returning to the frontline
• Responding to the call to arms
• Contracting COVID-19
• BAME ethnicities increased susceptibility
• Anxiety
• Appropriateness of skillset for return to clinical duty
• Inability in short-term to conduct research
• Impact on long-term research career</td></tr>
<tr><td>Support
• Funder support (extensions & network)
• Supportive networks (local, national, international)
• Provision of childcare for keyworkers
• Camaraderie</td><td>Protected characteristics
• BAME
• Maternal status
• Gender stereotypes
• Intersectional identities</td><td>Misconceptions and absence information
• Funding
• Extensions
• Lockdown restrictions being lifted
• Fiscal impact and predicted recession
• Portfolio development due to loss of research capacity
• Future impact of COVID
• Uncertain job market</td></tr>
</table>

For peer review only

**Maintenance of normality**<br>• Job security<br>• Maintenance of structure<br>• Workload maintained	**Demand from teaching responsibilities**<br>• Switch to online teaching<br>• Lack of support for teaching activity<br>• Teaching is devalued	
**Technological advances supporting remote working**<br>• Maintenance of networks<br>• Inclusive approach to working	**Loss of resource**<br>• Loss of outputs<br>• Fiscal barriers<br>• Loss of research team & associated HR issues<br>• Decrease in academic capacity due to clinical workload<br>• Lack of research supervision<br>• Practical barriers (e.g. data storage)<br>• Absence of pastoral support	

For peer review only

Theme 1: Opportunities and Enablers

The pandemic presented many opportunities and enablers. These spanned academic and clinical work as well as participants' personal lives. Subthemes broadly fell into three categories; 1) new opportunities for research, 2) support, 3) maintenance of normality and 4) technological advances supporting remote working.

New Emerging Opportunities

Participants recognised the immediate opportunity to explore new avenues of research related to the pandemic.

> "*So I've been asked to, and volunteered to, be part of the Research Ethics Committee Coronavirus Response which means that every week now I've had twenty-four hour turnaround for a coronavirus study of some sort or another … I have been involved with developing two national and one international coronavirus studies over this time…an enormously exciting but terrifying roll out of research…*" [Male Medic - diary]

> "*…there might be a positive if you were, you know, an Oxford University clinician looking at vaccine so you'd obviously you get a lot of funding and, you know, good opportunity to, to research Covid based things… I suppose if you've never done a clinical trial before and you were a clinical academic, getting involved in some of the trials that they're doing at the moment, like the Recovery Trial, that could be a positive for you if you're in that particular situation…" [*Female Medic – interview*]*

Associated with this was the need for rapid research dissemination, providing clinical academics with a chance to develop their portfolio. This resulted in the forging of new research collaborations supported by the quick turnaround of ethical approval in order to roll out impactful research. More senior clinical academics reported opportunities for career development such as being invited to serve on Research Ethics Committees. In addition to the new research territory, COVID-19 presented a new lens through which participants were able to think about their existing research.

For some, salient amongst the opportunities was the free time which was created during the pandemic, particularly due to the absence of social commitments and commuting. Some clinical academics found this spare time to be an opportunity to focus on academic research activities and the production of impactful research. Others felt this was as an opportunity to focus on clinical work, as well as family and household commitments.

> **"***I guess it will give me a chance to focus on, you know, having a bit more time at home with family and, and just doing clinical work, which I think, you know, is what I wanted to focus on for the moment*." [Female Medic – Audio Diary]

Support

Participants presented examples of the positive impact of support networks locally, nationally and internationally. The abrupt emergence of COVID and subsequent lockdown meant that many clinical academics halted their academic work and returned to clinical practice. This created an element of uncertainty, and in some cases anxiety about completing their academic work. However, a widely circulated statement

For peer review only

from the UK's main funding bodies reassuring clinical academics that they would support the extension of research post-COVID was well received.

> "*The [funder] have said that they will support extensions if they're needed so I do have that to kind of fall back on*." [Female Medic – Audio Diary]

An immense amount of support was provided by academic organizations at the beginning of the pandemic. Support from supervisors, departments and funding bodies was identified as enabling and empowering for participants. For example, the provision of childcare nurseries, enabling clinical academics to return to full-time clinical practice when required.

Maintenance of Normality

'Maintaining the status quo' during the pandemic was an enabler for many. From the beginning of the pandemic many professions have seen large proportions of redundancy. However, this has not been the case for many of the clinical academics that participated in this study. Clinical academics who have had the flexibility of undertaking either clinical or academic work during the pandemic, were grateful and felt generally lucky to have a job during COVID and one they could go back to post-COVID. Opportunities that strengthened the resolve of participants included the option to flex hours between roles. Specifically, it was also noted that going back to clinical practice full-time was perceived as advantageous due to more structure compared to academic work. Additionally, the retention of social interaction, structure and workload was seen as an opportunity.

> "*In these times of COVID-19 some people have kind of lost their structure and workload, so I feel grateful that hasn't happened to me*." [Male Medic – Audio Diary]

Technological advances supporting remote working

Participants advocated the use of video-conferencing platforms, citing them as enablers to maintain networks and collaborations during the pandemic as well as the inclusivity afforded by digital spaces.

> *"…my colleagues have become much more able to work online using Microsoft Teams and there have been more meetings recorded and more webinars which meant that as a part-time person I've been able to attend some of the things that previously I used to enquire and they used to say that this is not recorded and it is not being broadcast, so that has been excellent." [Female Medic – Interview]*

<u>Theme 2: Barriers</u>

There were multifaceted barriers related to the direct consequences of the immediate impact, reactive and mid-term barriers, and longer-term implications. Three subthemes were identified; (1) lockdown, (2) demand of teaching responsibilities and, (3) loss of resource.

Lockdown

For peer review only

Most of the barriers described by participants related to the negative consequences of lockdown. A barrier evidenced by particularly emotive data was that of working at home. This entailed the need to balance the multiple commitments related to childcare, housekeeping, and productivity. There were far reaching consequences of the ways in which working from home impacted on participants' roles. Childcare was not seen as compatible with a clinical academic role. Both clinical (e.g. telephone clinics and clinical administration) and academic work was often taking place at home, leading to feelings of isolation.

> "*I've spent a lot of time doing very intensive blocks of clinical work and very intensive blocks of research, research policy into practice work…I realised it's a Thursday today, I realised I had worked for three weeks solid and I'd not had a full day off in that time and most of those days were ten or twelve hour days…and I have now worked a further eleven days, with the last four days being fourteen hour days, without a break*". [Male Medic- Interview]

Mental and physical health outcomes were described as a consequence of COVID, particularly due to the increased workload.

> "*Generally, the COVID experience has been exhausting on many levels; emotionally from the worry about the number of people who will die, worry about being redeployed, worry for my children's wellbeing.*" [Male Medic-Interview]

> *"My anxiety is elevated due to being Asian, will I get sick and lose time for research?"* [Female Medic-Interview]

Concerns arose over how to effectively manage competing time demands. Some also spoke about a loss of 'downtime' and feeling 'stuck in limbo'. The shift in work-time meant that participants had less time for recreational activities which impacted on wellbeing.

Both males and females were impacted by the move to home working and balancing childcare with full-time work. The time pressures were apparent in many situations where participants struggled to navigate competing demands. There were also concerns over positive discrimination in how gender roles were recognised, with a particular focus on senior positions.

> "*… the Pro Vice Chancellor had sent an email saying how exciting it was that there's a female President at the Medical School, a female Dean, a female Pro Vice Chancellor of Medicine and a female Vice Chancellor at University, and that made me reflect what would happen if I sent a similar email delighting the fact that there's males in all of those roles. I imagine there'd be some disdain… they're powerful and take revenge…I don't think that's appropriate.*" [Male Medic – Audio Diary]

Demands from teaching responsibilities

The analysis identified the negative impact of having to rapidly produce teaching and assessment materials for online delivery, frequently coupled with a lack of support

and appreciation, as well as the time consumed. In many cases, this was in addition to clinical responsibilities.

> "*As clinical academics we are scavenging time in between the clinical parts to crack on and do our academic work*." [Male Medic – Audio Diary]

There was worry over a future lack of recognition within portfolios for teaching activities undertaken, and a frustration over negotiating the various new technologies required to deliver teaching.

Loss of resource
The impact of COVID has been significant for participants and extends from fiscal and time to human resource issues. There were concerns over the loss of time in relation to academic outputs and not fulfilling previously arranged activities. A lack of overall supervision was noted, including much needed pastoral support.

> "*There is an absence of pastoral support. Supervision is different, it's about skills and development not always about just how you feel. Sometimes you need a shoulder to cry on*". [Female Medic – Audio Diary]

Given the measures in the academic field where outputs are often used as a productivity marker, the participants were unsure how they could compensate loss of time and the ways in which their activities could be demonstrated and valued.

Tangible barriers were highlighted in the monetary implications for how loss of time could be compensated. Participants made calls for funders to extend time and funding for projects that otherwise may have been completed on time.

> "*It's bloody terrifying when it comes to applying for new stuff though because the deadlines haven't really moved much and there's no head space and there's no time to get the stuff really done properly and that's really scary but we've got to get the clinical work done first. It's the only way*." [Male Medic – Audio Diary]

Many universities initiated a recruitment freeze which meant in some cases that methodological expertise was lost. As clinical academics are often on a set educational employment path for a discrete time period this created disruption to long-term career plans.

Theme 3: Personal Characteristics and Social Identity

A key theme revealed was the impact of COVID-19 on participants' personal characteristics and social identity. This theme specifically considers how individuals perceived themselves as being clinical academics and the issues associated with the label 'clinical academic'.

Ambivalence and identity of a clinical academic
Some participants were conflicted about their identity as clinical academics and often experienced 'imposter syndrome'. Imposter syndrome, as described by the participants, refers to a feeling that they were not adequately qualified to

be called clinical academics. Others expressed pride in being classed as clinical academics, however, they expressed that their identity as clinical academics was misunderstood by clinical and academic colleagues. Participants described problems experienced with negotiating both clinical and academic identities during the pandemic.

> "*Trying to tussle where you fit between university and clinical world at the moment is, is also hard*." [Male Medic– Audio Diary]

Participants highlighted that in their working life they do not sit neatly in either category, which makes it difficult to identify as either an academic and/or a clinician. This feeling has been intensified by the pandemic. Most professionals have an educational background where they have developed peer support and networks allowing for guidance, but for clinical academics, their varied pathways means they often lack this important support network.

Protected characteristics

Protected characteristics of clinical academics, particularly the BAME community who have been more widely affected by COVID-19, was a key subtheme identified. Increased anxiety was highlighted and participants who were from BAME backgrounds expressed fear about returning to clinical work. Muslim clinical academics also expressed the difficulty they faced due to Ramadan taking place during the already difficult time. The focus was largely on physical wellbeing due to heavy workload alongside being unable to eat or drink.

Gendered differences were evident throughout this study, with female participants specifically expressing the struggles of maternal identity, highlighting problems faced with childcare responsibilities as well as having to share these with their partners alongside work commitments.

> *"I'm not formally planning to carry on with academia and that's a decision that's kind of come about because of, you know, childcare responsibilities and I just felt that I was going to be stretched too thin if I was trying to be a mum and be a GP and be an academic as well*." [Female Medic– Audio Diary]

Female participants felt that due to gender stereotypes from partners and work colleagues they were having to take a leading responsibility in childcare during this time.

> "*I think that COVID has been a disaster for feminism...the disagreements that I've had with [husband] have been over feeling like a 1950s housewife suddenly then to suddenly home schooling children and trying to stay sane, stay safe, keep everybody okay, whereas he still is doing exactly what he does, work...*" [Female Medic– Audio Diary]

Whilst participants noted intersectionality, their narratives were mostly differentiating their experiences due to gender. Women explicitly stated that they became less tolerant of the gender issues raised as time in lockdown progressed.

For peer review only

Women reported the emotional turmoil associated with being primary caregivers.

> *"Yet another day of working from home while helping with young kids who are upset at the disruption to their lives. The most difficult thing is trying to calm my children's' worry over COVID."* [Female Dentist– Audio Diary]

Increased perceived importance of clinical academics

Despite the tensions that have been caused by the pandemic, some noted that the work and identity of clinical academics have actually been strengthened. COVID has required rapid research to be undertaken and clinical academics are perfectly suited to this opportunity. The fact that qualified clinical academics have a foot in both research and clinical practice has facilitated a renewed appreciation of their identity during this pandemic.

Theme 4: Future fears and uncertainty

This theme related to numerous unanswered questions of participants including their fears and anxieties. Returning to the clinical environment was one major cause of anxiety, as well as the future of their research and funding.

Returning to the frontline

Discourse regarding a full return to clinical practice utilised many analogies to warfare. One source of internal conflict for participants was their inability to respond positively to the 'call to arms', particularly where they felt they had deskilled in some areas due to their focus on academia in recent years. Participants described requests to abandon research and assume clinical roles. Added to this anxiety was an inability to manage workload and family life.

> "*Well all of the academic trainees have been pulled on to the full-time clinical work. As clinical academics we are scavenging time in between the clinical parts to crack on and do our academic work and certainly emotionally I'm finding it enormous strain, mainly because the clinical work itself has not so much increased in the intensity but the massive decrease in staff numbers, partly because of illness but a lot because of isolation and shielding requirements that have gone on, has hugely increased the amount of work that all of us that are at work are doing*." [Male Medic– Audio Diary]

Additionally, the fear of contracting COVID-19 was expressed, with the majority of participants having to return to full clinical practice. CAs who expressed fear of contracting COVID-19 were those with predisposing factors and BAME backgrounds. Additionally, knowing someone who had died on the frontlines increased clinical academics' perception of their own risk.

Misconceptions and concerns about the future

From the onset of the pandemic, participants have reported concerns over their progression and future careers. Participants also aired concerns about the lockdown restrictions being lifted prematurely, stating that this could have further negative implications on their clinical and academic work. Uncertainty around COVID-19 has caused misconceptions, concerns and scaremongering, ranging from the prospect of more lockdowns to the potential for a return to fulltime clinical work only.

For peer review only

There has also been an absence of concrete information for clinical academics. Amongst participants, there is a general fear and uncertainty about the fiscal impact on research funding and the future clinical academia.

> "*Unfortunately my grant holders haven't sort of committed themselves to being able to extend my funding due to various reasons, so yeah, so there's uncertainty with that as well.*" [Female Medic– Audio Diary]

Issues such as pay cuts and a loss of staffing resources within academic departments caused further anxieties.

> *"The news on pay cuts is a bitter pill to swallow when working in a department where teaching commitments have increased, I have to say. Clinicians may decide to switch to clinical only in some cases.*" [Male Medic– Audio Diary]

These anxieties have been further intensified following a lack of professional and social networking opportunities, especially as the pandemic intensified and lockdowns progressed.

Box 1: Case examples (CE) of the initial impact of COVID-19 on participants with intersecting identities (pseudonyms utilised).

Box 1: Case studies of the impact of COVID-19 (pseudonyms utilised).

Case study 1: Asian, Mother, Muslim, Medical Registrar/Doctoral Fellow
Zainab, a Medical Registrar in Neurology and Doctoral Fellow is a married, Asian, Muslim, mother of three young children. She is 'proudly intersectional'. Zainab works full time and spends about 90% of her time on her academic work. She has recently put her details down to be called to the frontlines and is currently on the standby list. As an Asian doctor, living and working in a city, she feels particularly vulnerable and has fears about her risks of contracting COVID. Her anxieties regarding the virus increased over time as research linked BAME ethnicity to outcomes. She knows of a doctor who died on the front line, they were a relative of one of her closest friends. Although she realizes that susceptibility to the disease is multifactorial, she can see that people from minority ethnic groups are disproportionately affected. According to her, "*it's not known why [there's a disproportionately higher number] I think it is multifactorial whether there's underlying kind of health vulnerabilities that are more prevalent in certain ethnic groups that make them more vulnerable or whether there's more complex issues to that*." Despite the negative implications that the COVID pandemic and lockdown have brought, she finds a great deal of positives from the supportive academic institution.

Case study 2: White, Mother, Clinical Lecturer
Rose, a Medical Registrar and Academic Clinical Lecturer, has been working at home where she is married to a clinical professor with two children. Rose works part-time. She is struggling with the balance of family and work commitments, stating that lockdown has reinforced sexist gender roles. Rose feels that her research has taken a backseat while her husband's work has been prioritised, "*I genuinely think he thinks his work is more important, I genuinely think that's what he believes*." She reports a decline in mental health due to the pressures of lockdown and family dynamics, "*I think that COVID has been a disaster for feminism...the disagreements that I've had with [husband] have been over feeling like a 1950s housewife suddenly, then to suddenly have to provide home schooling children and trying to stay sane, stay safe, keep everybody okay, whereas he still is doing exactly what he does [work]...so I think that, that, that's my big worry is that actually COVID's been a step back*." Rose's anxiety about her research profile increased the longer lockdown restrictions were in place.

Discussion

This study presents a unique, qualitative data-driven reflection of the national clinical academic landscape during nine months of the COVID-19 pandemic. The in-depth interview data are supported by real time audio diaries. This multi-faceted approach has facilitated access to “periods of change and flux” particularly during the pandemic, and allowed the research team to capture the impact of COVID-19 in real-time which facilitated accurate data capture [21, 22]. The audio diaries are also advantageous owing to their ease of completion, which typically results in lower levels of attrition. [22] Participants were also permitted to submit written entries, but this was not deemed to be a limitation, especially as discourse analysis was not planned. Permitting flexibility also prevents diary entries from becoming formulaic [22]. Whilst the authors recognize that there is often a self-selection bias associated with studies of this nature, participants were felt to be balanced in their presentation of barriers and enablers [23]. Audio diaries varied in length and thus depth, however, there was no variability in the richness of data.

Clinical academics were impacted by COVID-19 in multiple ways leading to a wide range of barriers, challenges and opportunities underpinned by their personal and professional identities. The selflessness and dedication of the study participants to contribute to the clinical response to the COVID-19 pandemic is evident in the data. Clinical academics returning to the NHS during the pandemic, had added value with respect to their research experience[24].

Our findings identified the numerous perceived barriers to continuing academic activity within the family, academic and clinical contexts. What is clear is that pre-existing barriers to academic activity have become magnified during the COVID-19 pandemic. Although such barriers are not insurmountable, they have proved and continue to prove stressful for the participants. The impact on their future career plans is already evident. The restrictions on face-to-face contact, international travel, uncertainties over clinical and academic training and funding extensions, home working, and, in many cases, redeployment to frontline clinical duties have all impacted on academic activities. Tension was evident when academic trainees who switched to full time clinical work and sacrificed their academic time described how they felt disadvantaged in comparison to trainees who have been able to maintain research.

Female participants described barriers that related to their gender, as well as for many, their maternal status, with such biases having been previously well documented in the literature [18, 20, 25-27]. Our findings in the themes (2 and 3) and case studies, suggest that the pandemic and resultant lockdown has, in many cases, exacerbated the discrimination faced by many women in clinical medical and dental academia. A survey of dental clinical academics in 2018, found that overall female representation in the academic grades in Dentistry is increasing - mirroring gender representation in dentistry as a whole [28]. Given our data, it is possible that the COVID-19 pandemic could have an even more significant negative impact on female Dental Clinical Academics going forward.

Theme three also identified a number of females in our sample who described feeling and being compelled to assume, to a greater degree than before the pandemic, gender stereotypical roles in the home. Specifically, when faced with competing responsibilities females appeared to feel under more pressure than their male partners to allocate more time to childcare and household tasks. Indeed, there was a recurrent motif of narratives relating to the '1950s housewife' stereotype within the family context [29]. In this regard, our findings are consistent with media accounts that the lockdown has placed a disproportionate burden on females, who frequently report taking on the bulk of the childcare, domestic duties and home-based education of school-aged children [30, 31]. It has been postulated that many females are currently doing 'second and third shifts' with regard to housework and welfare after their first shift of childcare or paid work [29]. Prior to COVID-19, there was evidence that parental working determined that, on average, females spent two to three times as much time on care and housework compared to men [32]. Our findings suggest that these effects have become more pronounced as a result of the pandemic and are impacting significantly on female clinical academics. Our data may go some way towards explaining reported declines in publications by female academics [33].

The findings raise an alarm that prolonged periods of lockdown could reverse general trends towards a more equitable distribution of household labour [32]. More concerningly, it may substantially put back efforts to achieve a more gender representative body of clinical academics, characterised by gross under representation of female, explored by a recent systematic review [9]. This has been shown to not merely be a matter of social equality; the male dominance of science in general has served women's health poorly [34].

Of note within our data was the indication from male participants that they were experiencing positive discrimination. We would not wish to overinterpret, however, our analysis of the participants' narratives alluded to male fragility. In women's continued fight for equality within the workplace can often lead to discomfort from those who have typically held most privilege within the discussion. Although the discomfort articulated from males in our sample was a minority, it was expressed with conviction.

There are a number of steps that could be taken to mitigate the impact of the COVID-19 pandemic on clinical academia as a whole and the risk of reduced long-term clinical research capacity, especially the loss of female academics. Funders and employers could intentionally target support at those with caring responsibilities during lockdown and the following recovery period. As the pressing clinical need recedes then prioritising the return of female academics to their research activities may also offset the bias more generally experienced by female scientists both before and during the pandemic. Whilst virtual networks of collaborators have organically sprung up in places during the lockdown, more of these could be strategically formed to counter the isolation felt by many academics. This support should also extend to academics who are primarily clinical educators, and have historically felt marginalised and undervalued [35]. Indeed, some of the participants in the present study voiced perceptions of a lack of support and appreciation from both their academic and clinical employers, and colleagues. Both existing and trainee clinical

For peer review only

academics reported a perception that neither non-academic clinical colleagues or non-clinical academics fully appreciated the competencies and the time demands of their academic roles. This perception has been reinforced by the COVID-19 pandemic. Going forward, enhanced collaboration and empathy between University and Clinical staff and employers could be targeted to remedy this.

As the UK navigates its immediate response to COVID-19, it is important to consider the potential implications for those whose careers are most vulnerable and require additional nurturing. There needs to be increased flexibility in career pathways and expectations, along with a review of funding and support offered. Participants requested flexibility with existing funding to make changes to study protocols and costings to enable them to pursue opportunistic COVID related research. Leaders in academic institutions and funding bodies must be cognisant of the varying degrees to which clinical academics will have been able to mitigate for COVID-19 in their assessments of applications for funding and positions and must revise their expectations for dissemination and impact. Causal explanations of the issues faced during COVID-19 will be multifaceted and sensitive to personal context. Teaching commitments must be valued and accounted for, as well as physical and mental health. It has been shown that clinical academics create national wealth as well as health [36]. We are heading into an inevitable global financial recession, if not depression, likely to also be characterised by an increase in mental and physical health needs which will undoubtedly impact on the clinical academic workforce and funding.

The infrastructure for medical and dental clinical academia is well established, yet our data suggest that better support is required to develop future clinical academic roles and enhance the provision of evidence-based patient care in the post-COVID-19 era. Participants report substantiated concerns over access to funding and positions once the status quo is resumed.

Whatever interventions and strategies are implemented, heed needs to be taken to the findings of Laver et al., who reported that interventions within clinical academia that require those they are designed to support, so called ‘bottom-up’ approaches, to bear the brunt of the workload are less likely to succeed [37]. Their review advocates the use of ‘top-down’ approaches, led by change in practice at a management level. Finally, their stance was that ‘something is better than nothing’.

Our findings offer further insight into the debate surrounding access to, and maintenance of, clinical academic careers [38]. We add weight to the evidence surrounding gender disparity. The well documented decline in the clinical academic workforce, the subject of contemporary research, [9, 39] could continue if strategies to support clinical academics are not implemented in the aftermath of the COVID-19 pandemic. Ongoing commitment to supporting and developing clinical academics is required, acknowledging the implicit biases facing females and parents in reaching their potential, and those with other competing demands on their time. Of paramount importance is the need for greater recognition of the value and contribution made by clinical academics to both the clinical and academic workforces to which they contribute. The reports from a recent national study by Finn and colleagues presented a number of proposed interventions to support clinical academics in the immediate wake of the pandemic, in addition to recommendations for ensuring the

long-term sustainability of the clinical academic pipeline. Headlines from qualitative data included the need for mentorship, flexibility and transparent processes, as well as recognition of the differential experiences of marginalised groups[10].

Our findings also emphasise the need for guidance for aspiring clinical academics. As one remarked, “I just need guidance on next steps. It is just so challenging to think about who might help me.” Furthermore, without urgent action COVID-19 has the potential to increase the gender divide seen within clinical academia, and as demonstrated within recent studies [40-42]. It is hoped that the narratives in this study initiate a dialog between clinical academics, funders and institutions to successfully navigate the clinical academic pathways moving forward. Support and mentoring may be key to ensuring the clinical academic pipeline.

Declaration

The manuscript is an honest, accurate, and transparent account of the study being reported. There are no omissions or inaccuracies. Participants have engaged in member checking of results and are briefed of findings.

Funding

This publication presents independent research funded by Cancer Research UK (Award Number: C71037/A29824), The Academy of Medical Sciences, Health Education England, The Medical Research Council, The National Institute for Health Research (NIHR) and The Wellcome Trust. The views expressed are those of the author(s) and not necessarily those of The Academy of Medical Sciences, Cancer Research UK, Health Education England, The Medical Research Council, The National Institute for Health Research (NIHR), The Wellcome Trust or the Department of Health and Social Care.

Acknowledgements

The authors wish to thank all the clinical academics who participated in this study. The authors acknowledge the work of the wider research team involved in this large-scale research project and thank them for their contributions. The authors also appreciate the insight of the project steering group at the Clinical Academic Training Forum. Thanks go to ‘Women in Academic Medicine’, and the British Medical Association for assistance in recruiting participants.

Data sharing

Deidentified transcripts would be made securely available to other researchers if a written case for access is made to the research team and permission is granted by the funders of the study

Author contributions

GF, PC, PT, MK, JM and JB conceived the study. GF, PC, PT, JB and JM obtained funding. ET, GF, JB, PC, ABK and AK collected data and conducted the analyses. GF, AK, JB, PT and PC recruited participants and drafted the manuscript. ABK and AK curated the data. All authors reviewed the final manuscript.

References

For peer review only

1. Brenner, A.M., *Supervising the tearful trainee: opportunities for professional growth and learning.* Academic psychiatry : the journal of the American Association of Directors of Psychiatric Residency Training and the Association for Academic Psychiatry, 2014. **38**(4): p. 497-501.
2. Hourston, G.J., *The impact of despecialisation and redeployment on surgical training in the midst of the COVID-19 pandemic.* International Journal of Surgery (London, England), 2020. **78**: p. 1.
3. Clinical Academic Training Forum, *Clinical Academic Training Forum: Subgroup to support returning clinical academics from the COVID-19 front line*. 2020.
4. Guarino, C.M. and V.M. Borden, *Faculty service loads and gender: Are women taking care of the academic family?* Research in Higher Education, 2017. **58**(6): p. 672-694.
5. Ranieri, V., et al., *Factors that influence career progression among postdoctoral clinical academics: a scoping review of the literature.* BMJ Open, 2016. **6**(10): p. e013523.
6. Flaherty, C. *No Room of One's Own: Early journal submission data suggest COVID-19 is tanking women's research productivity*. 2020 [cited 2020 21st April 2020]; Available from: https://www.insidehighered.com/news/2020/04/21/early-journal-submission-data-suggest-covid-19-tanking-womens-research-productivity.
7. Pinho-Gomes, A.-C., et al., *Where are the women? Gender inequalities in COVID-19 research authorship.* BMJ Global Health, 2020. **5**(7): p. e002922.
8. McEwan, J., *Engaging women in academic medicine in the UK: report of a workshop at the Association of Physicians Annual Meeting, 2 April 2009.* QJM: An International Journal of Medicine, 2010. **103**(9): p. 635-639.
9. Brown, J.V., et al., *From the sticky floor to the glass ceiling and everything in between: protocol for a systematic review of barriers and facilitators to clinical academic careers and interventions to address these, with a focus on gender inequality.* Systematic reviews, 2020. **9**(1): p. 26.
10. Finn GM, B.J., Evans C, Raine G, Stewart L, Uphoff E, Kehoe A, Buchanan J, Balogun-Katung A, Tiffin P, Taylor E, Crampton P, Morgan J., *From the sticky floor to the glass ceiling and everything in between: A systematic review and qualitative study focusing on gender inequalities in Clinical Academic careers. Final report.* . 2020.
11. Finn GM, B.J., Evans C, Raine G, Stewart L, Uphoff E, Kehoe A, Buchanan J, Balogun-Katung A, Tiffin P, Taylor E, Crampton P, Morgan J., *Inequalities in UK clinical academic careers: a systematic review and qualitative study. Short report.* . 2020.
12. Gordon, L., et al., *Multiple and multidimensional transitions from trainee to trained doctor: a qualitative longitudinal study in the UK.* BMJ open, 2017. **7**(11).
13. Braun, U.K., et al., *The utility of reflective writing after a palliative care experience: Can we assess medical students' professionalism?* Journal of Palliative Medicine, 2013. **16**(11): p. 1342-1349.
14. Braun, V. and V. Clarke, *Using thematic analysis in psychology.* Qualitative research in psychology, 2006. **3**(2): p. 77-101.
15. Kiger, M.E. and L. Varpio, *Thematic analysis of qualitative data: AMEE Guide No. 131.* Medical Teacher, 2020: p. 1-9.

For peer review only

16. Roberts, K., A. Dowell, and J.-B. Nie, *Attempting rigour and replicability in thematic analysis of qualitative research data; a case study of codebook development.* BMC medical research methodology, 2019. **19**(1): p. 66.
17. Braun, V. and V. Clarke, *Successful qualitative research: A practical guide for beginners*. 2013: sage.
18. Williams, J.C. and N. Segal, *Beyond the maternal wall: Relief for family caregivers who are discriminated against on the job.* Harv. Women's LJ, 2003. **26**: p. 77.
19. Brown, M.E., et al., *'Too male, too pale, too stale': a qualitative exploration of student experiences of gender bias within medical education.* BMJ open, 2020. **10**(8): p. e039092.
20. Williams, J.C., *Hitting the maternal wall.* Academe, 2004. **90**(6): p. 16.
21. Monrouxe, L.V., *Negotiating professional identities: dominant and contesting narratives in medical students' longitudinal audio diaries.* Current Narratives, 2009. **1**(1): p. 41-59.
22. Crozier, S.E. and C.M. Cassell, *Methodological considerations in the use of audio diaries in work psychology: Adding to the qualitative toolkit.* Journal of occupational and organizational psychology, 2016. **89**(2): p. 396-419.
23. Collier, D. and J. Mahoney, *Insights and pitfalls: Selection bias in qualitative research.* World Politics, 1996. **49**(1): p. 56-91.
24. Rimmer, A., *Covid-19: How clinical academic trainees came back to the wards en masse.* bmj, 2021. **373**.
25. Crosby, F.J., J.C. Williams, and M. Biernat, *The maternal wall.* Journal of Social Issues, 2004. **60**(4): p. 675-682.
26. Williams, J.C., *The glass ceiling and the maternal wall in academia.* New Directions for Higher Education, 2005. **2005**(130): p. 91-105.
27. Halley, M.C., et al., *Physician mothers' experience of workplace discrimination: a qualitative analysis.* BMJ, 2018. **363**: p. k4926.
28. Council, D.S., *Professorial roles for dentists fall 8.3% since 2015.* British Dental Journal, 2018. **225**(5).
29. Chung, H. *Return of the 1950s housewife? How to stop coronavirus lockdown reinforcing sexist gender roles*. 2020; 21st April 2020]. Available from: https://theconversation.com/return-of-the-1950s-housewife-how-to-stop-coronavirus-lockdown-reinforcing-sexist-gender-roles-134851.
30. Ferguson, D., *'I feel like a 1950s housewife': how lockdown has exposed the gender divide*, in *The Guardian*. 2020.
31. Alon, T.M., et al., *The impact of COVID-19 on gender equality*. 2020, National Bureau of Economic Research.
32. Wishart, R., et al., *Changing patterns in parental time use in the UK*. 2019.
33. Viglione, G., *Are women publishing less during the pandemic? Here's waht the data say*, in *Nature*. 2020. p. 365-366.
34. Burford, B., et al., *Professionalism education should reflect reality: findings from three health professions.* Medical Education, 2014. **48**(4): p. 361-374.
35. Kumar, K., C. Roberts, and J. Thistlethwaite, *Entering and navigating academic medicine: academic clinician-educators' experiences.* Medical education, 2011. **45**(5): p. 497-503.
36. Innovation, B. and G. Team, *Bioscience 2015: Improving National Health.* Increasing National Wealth, 2003.
37. Laver, K.E., et al., *A systematic review of interventions to support the careers of women in academic medicine and other disciplines.* BMJ open, 2018. **8**(3).

38. Rothwell, P.M., *Medical academia is failing patients and clinicians*. 2006, British Medical Journal Publishing Group.
39. Fitzpatrick, S., *A survey of staffing levels of medical clinical academics in UK medical schools as at 31 July 2011.* London: Medical Schools Council, 2012.
40. Meho, L. I. (2021). The gender gap in highly prestigious international research awards, 2001–2020. Quantitative Science Studies (MIT Press), 2(3), 976–989. https://doi.org/10.1162/qss_a_00148
41. Ribarovska, A. K., Hutchinson, M. R., Pittman, Q. J., Pariante, C., & Spencer, S. J. (2021). Gender inequality in publishing during the COVID-19 pandemic. Brain, behavior, and immunity, 91, 1.
42. Kibbe, M. R. (2020). Consequences of the COVID-19 pandemic on manuscript submissions by women. JAMA surgery, 155(9), 803-804.

For peer review only

Appendix 1: Interview and example audio dairy prompts for participants during COVID-19

Example interview stems for participants during COVID-19
Semi-structured approach thus will be guided by participants.<br>1. **Introductions**<br><br>2. **Please describe your role and career path to date -** *both clinical and academic*<br><br>3. **What are your experiences of clinical academia?**<br><br>4. **What factors impacted upon your decision to pursue (or not pursue) a career in clinical academia?**<br>• *What factors make clinical academia an attractive career?*<br><br>5. **What has your experience with awards/ funding been like? (if any)**<br>• *Application process? (facilitators/barriers)*<br>• *Support through process*<br>• *Why did/didn't continue? (e.g. after PhD why didn't do lectureship?)*<br>• *If not applied, why not?*<br>• *Did this experience influence your choice in career pathway?*<br><br>6. **What barriers and enablers have you faced within clinical academia?**<br>• *What made you continue/not continue?*<br>• What stops people returning to clinical academia?<br>• How do you negotiate the 2 career paths?<br>• What goes wrong in institutions that funders can help with?<br>• What worked well/ less well with other funders you have experienced?<br>• Impact of the COVID-19 pandemic<br><br>7. **What could be done to improve equal representation within clinical academia?**<br><br>8. **Describe any interventions to promote access to clinical academia or reduce discrimination that you have experienced.**<br>• *What worked?*<br>• *Given your context, what would you recommend?*<br><br>9. **What existing or new interventions could help to reduce attrition in clinical academic careers?**<br><br>10. **How can organisations support trainees and clinical academics in their career decisions and academic pathways?**
**Example audio dairy prompts for participants during COVID-19**
Participants were able to reflect on all prompts any situation pertaining to Clinical Academia that they wished. Prompts were broad and participants were informed of the interest in protected characteristics.

How has COVID-19 impacted upon your work as a clinical academic?	What have you heard about funding (opportunities, threats, remit etc) post COVID-19?	Has COVID-19 had a detrimental impact on or changed your career plans?
How has the expectation of the impact of COVID-19 differed from the reality?	How have your protected characteristics impacted upon your experience of clinical academia during COVID-19?	BAME participants: Has the emerging data on the enhanced risk to the BAME workforce from COVID-19 had any impact on you?
Has COVID-19 had a detrimental impact on or changed your research?	What barriers and enablers have you faced during COVID-19?	Parents/ carers: How have your responsibilities impacted upon your ability to work?

For peer review only

Reference:

Tong A, Sainsbury P, Craig J. Consolidated criteria for reporting qualitative research (COREQ): a 32-item checklist for interviews and focus groups. International Journal for Quality in Health Care. 2007. Volume 19, Number 6: pp. 349 – 357

Consolidated criteria for reporting qualitative studies (COREQ): 32-item checklist

No	Item	Guide questions/description
		**Domain 1: Research team and reflexivity**
Personal Characteristics		
1.	Interviewer/facilitator	Which author/s conducted the interview or focus group? GF, MK, PC, JAG, AB
2.	Credentials	What were the researcher's credentials? *E.g. PhD, MD* The researchers had a range of qualifications including PhD, MD, MBChB, MMEd, PGDipMEd, MRCpsych, FRCP, FAcadMEd, FRCPsych, and MSc.
3.	Occupation	What was their occupation at the time of the study? GF was Professor of Medical Education, MK and AB were Research Fellows, ET was a medical student. PAT, JM, CS and JAG were clinical academics.
4.	Gender	Was the researcher male or female? Three were male, 6 were female
5.	Experience and training	What experience or training did the researcher have? All trained in research methods and well published in the field.
Relationship with participants		
6.	Relationship established	Was a relationship established prior to study commencement?

For peer review only

No	Item	Guide questions/description
		No
7.	Participant knowledge of the interviewer	What did the participants know about the researcher? e.g. *personal goals, reasons for doing the research*<br><br>Participants were provided with an information sheet outlining the reasons for doing the research. They had the opportunity to ask questions and were able to access a study website and twitter account for information.
8.	Interviewer characteristics	What characteristics were reported about the interviewer/facilitator? e.g. *Bias, assumptions, reasons and interests in the research topic*<br><br>A reflexivity exercise was conducted to demonstrate the researchers' relationship to the study findings. This is provided on the project website.
		**Domain 2: study design**
Theoretical framework		
9.	Methodological orientation and Theory	What methodological orientation was stated to underpin the study? *e.g. grounded theory, discourse analysis, ethnography, phenomenology, content analysis*<br><br>Reflexive thematic anaysis
Participant selection		
10.	Sampling	How were participants selected? *e.g. purposive, convenience, consecutive, snowball*<br>Purposive with snowballing
11.	Method of approach	How were participants approached? e.g. *face-to-face, telephone, mail, email*

For peer review only - http://bmjopen.bmj.com/site/about/guidelines.xhtml

No	Item	Guide questions/description
		Social media, email listserv, referrals
12.	Sample size	How many participants were in the study?<br>104 in main dataset
13.	Non-participation	How many people refused to participate or dropped out? Reasons?<br>0
Setting		
14.	Setting of data collection	Where was the data collected? e.*g. home, clinic, workplace*<br>Telephone interviews and audio diaries
15.	Presence of non-participants	Was anyone else present besides the participants and researchers?<br>No
16.	Description of sample	What are the important characteristics of the sample? *e.g. demographic data, date*<br>Clinical academics in the UK
Data collection		
17.	Interview guide	Were questions, prompts, guides provided by the authors? Was it pilot tested?<br>Guidance was provided for audio diaries, interviews used piloted stems
18.	Repeat interviews	Were repeat interviews carried out? If yes, how many?<br>No
19.	Audio/visual recording	Did the research use audio or visual recording to collect the data?<br>Audio

For peer review only - http://bmjopen.bmj.com/site/about/guidelines.xhtml

No	Item	Guide questions/description
20.	Field notes	Were field notes made during and/or after the interview or focus group?<br><br>Yes – field notes made
21.	Duration	What was the duration of the interviews or focus group?<br>45-60 mins
22.	Data saturation	Was data saturation discussed?<br>Yes
23.	Transcripts returned	Were transcripts returned to participants for comment and/or correction?<br>Upon request
		**Domain 3: analysis and findings**
Data analysis		
24.	Number of data coders	How many data coders coded the data?<br>5
25.	Description of the coding tree	Did authors provide a description of the coding tree?<br>Listed on our website
26.	Derivation of themes	Were themes identified in advance or derived from the data?<br><br>Mixture – some deductive and some inductive
27.	Software	What software, if applicable, was used to manage the data?<br>NVivo
28.	Participant checking	Did participants provide feedback on the findings?<br>Yes
Reporting		

For peer review only - http://bmjopen.bmj.com/site/about/guidelines.xhtml

No	Item	Guide questions/description
29.	Quotations presented	Were participant quotations presented to illustrate the themes / findings? Was each quotation identified? e.*g. participant number*<br><br>Yes
30.	Data and findings consistent	Was there consistency between the data presented and the findings?<br><br>Yes
31.	Clarity of major themes	Were major themes clearly presented in the findings?<br><br>Yes
32.	Clarity of minor themes	Is there a description of diverse cases or discussion of minor themes?<br><br>Yes

For peer review only

BMJ Open

A qualitative exploration on the impact of the COVID-19 pandemic on clinical academics through the lens of protected characteristics

Journal:	*BMJ Open*
Manuscript ID	bmjopen-2021-057655.R2
Article Type:	Original research
Date Submitted by the Author:	11-Mar-2022
Complete List of Authors:	Finn, Gabrielle; The University of Manchester, Division of Medical Education, School of Medical Sciences, Faculty of Biology, Medicine and Health<br>Crampton, Paul; Hull York Medical School, Health Professions Education Unit<br>Buchanan , John; Barts and The London School of Medicine and Dentistry, Centre for Education and Innovation<br>Balogun, Abisola Olatokunbo; Hull York Medical School, Health Professions Education Unit<br>Tiffin, Paul; University of York, Health Sciences<br>Morgan, Jessica; University of York, Centre for Reviews and Dissemination; Leeds Teaching Hospitals NHS Trust, Department of Paediatric Oncology<br>Taylor , Ellie; Hull York Medical School, Health Professions Education Unit<br>Soto, Carmen; British Medical Association<br>Kehoe, Amelia; Hull York Medical School, Health Professions Education Unit
**Primary Subject Heading**:	Qualitative research
Secondary Subject Heading:	Medical education and training, Qualitative research, Research methods, Public health
Keywords:	COVID-19, QUALITATIVE RESEARCH, MEDICAL EDUCATION & TRAINING

SCHOLARONE™
Manuscripts

I, the Submitting Author has the right to grant and does grant on behalf of all authors of the Work (as defined in the below author licence), an exclusive licence and/or a non-exclusive licence for contributions from authors who are: i) UK Crown employees; ii) where BMJ has agreed a CC-BY licence shall apply, and/or iii) in accordance with the terms applicable for US Federal Government officers or employees acting as part of their official duties; on a worldwide, perpetual, irrevocable, royalty-free basis to BMJ Publishing Group Ltd ("BMJ") its licensees and where the relevant Journal is co-owned by BMJ to the co-owners of the Journal, to publish the Work in this journal and any other BMJ products and to exploit all rights, as set out in our licence.

The Submitting Author accepts and understands that any supply made under these terms is made by BMJ to the Submitting Author unless you are acting as an employee on behalf of your employer or a postgraduate student of an affiliated institution which is paying any applicable article publishing charge ("APC") for Open Access articles. Where the Submitting Author wishes to make the Work available on an Open Access basis (and intends to pay the relevant APC), the terms of reuse of such Open Access shall be governed by a Creative Commons licence – details of these licences and which Creative Commons licence will apply to this Work are set out in our licence referred to above.

Other than as permitted in any relevant BMJ Author's Self Archiving Policies, I confirm this Work has not been accepted for publication elsewhere, is not being considered for publication elsewhere and does not duplicate material already published. I confirm all authors consent to publication of this Work and authorise the granting of this licence.

For peer review only

A qualitative exploration on the impact of the COVID-19 pandemic on clinical academics through the lens of protected characteristics

*Gabrielle M Finn[1], Paul E S Crampton[2], John Buchanan[3], Abisola Balogun-Katung[2], Paul A. Tiffin[4], Jessica E Morgan[5,6], Ellie Taylor[2], Carmen Soto[7], Amelia Kehoe[2]

Affiliations

1. Division of Medical Education, School of Medical Sciences, Faculty of Biology, Medicine and Health, The University of Manchester, M13 9NT
2. Health Professions Education Unit, Hull York Medical School, University of York, York, UK, YO10 5DD
3. Queen Mary University London, Barts Health NHS Trust
4. Mental Health and Addiction Research Group, Department of Health Sciences, University of York, York, UK, YO10 5DD
5. Centre for Reviews and Dissemination, University of York, York, UK, YO10 5DD
6. Department of Paediatric Oncology, Leeds Children's Hospital, Leeds, UK, LS1 3EX
7. British Medical Association, London, WC1H 9JP

Corresponding author:
Professor Gabrielle M. Finn
Division of Medical Education, School of Medical Sciences, Faculty of Biology, Medicine and Health, The University of Manchester, M13 9NT
gabrielle.finn@manchester.ac.uk
Twitter: @gabs_finn

Word count: 5712

For peer review only

A qualitative exploration on the impact of the COVID-19 pandemic on clinical academics through the lens of protected characteristics

Abstract

Objective: To investigate the impact of the COVID-19 pandemic on the research activity and working experience of clinical academics (CAs), with a focus on gender and ethnicity.

Design: Interviews and audio/written diary study.

Setting: United Kingdom (UK) study within clinical academia

Participants: Purposive sample of 82 clinical academics working in medicine and dentistry across all career stages ranging from academic clinical fellows, and doctoral candidates to professors.

Methods: Qualitative semi-structured interviews (n=68) and audio diary data (n=30) collected over an eight-month period (January-September 2020), thematically analysed.

Results: Four major themes were identified in relation to the initial impact of COVID-19 on Clinical Academics; opportunities, barriers, personal characteristics and social identity and fears & uncertainty. COVID-19 presented opportunities for new avenues of research. Barriers included access to resources to conduct research and the increasing teaching demands. One of the most prominent sub-themes within 'personal characteristics' was that of the perceived negative impact of the pandemic on the work of female CAs. This was attributed to inequalities experienced in relation to childcare provision and research capacity. Participants described differential experiences based upon their gender and ethnicity, noting intersectional identities. Participants included 60 females and 30 males, 80% identified as white and 20% as Black, Asian or another minority (BAME).

Conclusions: Whilst there have been some positives afforded to CAs, particularly for new avenues of research, COVID-19 has negatively impacted workload, future career intentions, and mental health. BAME CAs were particularly fearful due to the differential impact on health. Our study elucidates the direct and systemic discrimination that creates barriers to women's career trajectories in clinical academia. A flexible, strategic response that supports CAs in resuming their training and research is required. Interventions are needed to mitigate the potential lasting impact on CA capacity from the pandemic, and the potential for the loss of women from this valuable workforce.

Strengths and Limitations of this study

- Study utilised both audio and written diary entries as well as in-depth interviews.

For peer review only

- Telephone and online interviews negated data collection issues with the pandemic.
- The large sample size balances the UK only nature of data.
- The research team was diverse in terms of gender, ethnicity and experience.
- Audio diaries enabled the exploration of sensitive issues.

Introduction

The workforce presence of clinical academics is vital to healthcare to ensure that scientific knowledge underpins advances in medical education, translational research and patient outcomes. Their low number and lack of senior pipeline progression is alarming, particularly so for under-represented groups including women and ethnic minorities.[1] The COVID-19 pandemic has further exacerbated the situation through rapid redeployment, burnout, reinforcement of gender and racial stereotypes, and financial inequity.[2]

Over 1,500 academic trainees in England were estimated to be redeployed to clinical work during the COVID-19 pandemic[3]. This equates to more than 90% of those on the Integrated Academic Training (IAT) pathway.[3] Following the closure of high-risk aerosol-generating dental services, some junior hospital dentists and dental clinical academics were also redeployed to provide support care for hospital services.[4] The potential impact on trainees' clinical experience has been recognised.[5] In terms of formally monitoring and permitting advancement in postgraduate training, 'no fault' Annual Review of Competence Progression (ARCP) outcomes (10.1 and 10.2) have been introduced where acquisition of competencies has been delayed owing to COVID-19[6]. Meanwhile, there has been a research paradox whereby some research institutions have closed, while others have adapted and embraced COVID specific research [7]. Further issues highlight the consumption of academic resources that may prove ineffective or wasteful owing to haste, rapid redesign of online teaching materials, and inadequate infrastructure and design.[8] With the current prioritization of COVID-19 studies, new clinical trials have been paused [9] with detrimental impacts on clinical academics working on non-COVID research.

Prior to the pandemic, it was recognised that females in medicine tended to take on more 'service work' and had less protected time for research than their male counterparts.[4] In addition to low representation of minority groups, the lack of role models provides a further barrier. [5] Collectively, gender and racial stereotypes, childcare and home-working all put further pressure on an already stretched workforce, with women now publishing less.[6, 7] Losing further women and minority groups from this fragile workforce could run the risk of long-term reduced diversity in medical education, less female-relevant clinical research and ultimately have a negative impact on patient outcomes, in addition to having less women visible in senior positions [8, 9].

We sought to explore discrimination based upon protected characteristics in the clinical academic workforce, at various stages of the pipeline during the pandemic. We aimed to understand the barriers, enablers and interventions to facilitate clinical academic careers, thereby to develop key recommendations to support and reduce

attrition of these exceptionally talented individuals. We were particularly interested in the experiences of women and clinical academics from different ethnic groups.

Methods

Ethics
Ethical approval for this study was obtained from the Hull York Medical School Ethics Committee (ref: 19 32).

Context
Prior to the COVID-19 pandemic, the authors were engaged in a multi-funder research project exploring barriers and facilitators to a career in UK clinical academia[10, 11]. As data were iteratively analysed, the impact of COVID-19 on clinical academia progressively shaped the experiences of the participants and relevant themes emerged. In order to assess the impact of COVID-19, the data collected after the first confirmed case within the UK (29th January 2020) until the end of September 2020 were isolated for analysis. This period included the first period of significant national restrictions ("lockdown") from 26th March to a phased reopening in June.

This study was conducted within a social constructivism orientation, additionally informed by the multiple principles of feminist theory. Feminist theory holds a constructivist ontology.

Patient and Public Involvement
The study had a steering group which included Patient and Public representation, provided by Health Watch

Study design and recruitment
Qualitative interviews and longitudinal qualitative audio and written diary entries were utilised. Purposive sampling was undertaken, using social media, funder mailing lists and snowballing. Inclusion criteria specified participants were a doctor or dentist, based within the UK and working as or towards becoming a clinical academic. For the purposes of the study, a 'clinical academic' was defined by individuals themselves and how they perceived their roles. Recruitment was through various avenues; a website, emails sent on behalf of the research team by stakeholder organisations; social media recruitment; and 'snowballing'. Authors had no relationship with participants prior to the study.

Data collection
Data were collected using (1) audio and written diary entries, and (2) semi-structured interviews. Diaries enabled participants to discuss their views on a wide range of factors related to clinical academic careers, and latterly their experiences of working during COVID-19. The diary method enabled researchers to collect 'novel' and timely data during the transition to lockdown phase, a critical moment in healthcare. Many diaries were recorded immediately after events, such as discriminatory remarks

For peer review only

being made in a workplace. Participants were invited to submit audio diary entries (MP3 files) via email or attached to an encrypted message. Submissions were sent to a professional account linked to the project following best practice.[12] In order to promote engagement, participants were also permitted to write their diary entry.[12] Participants were able to submit diaries as frequently as they wished, each acknowledged by the researchers to maintain rapport and engagement. Participants were issued with a guide to follow which was informed by a concurrent systematic review.[9] The guide suggested frequencies of entries, instructions for submission and indicative content (Appendix 1). Reminder emails were sent monthly. Interviews were conducted via video conferencing or telephone with the use of a topic guide. Audio diary files and interviews were recorded and transcribed verbatim. All participants were given an electronic information sheet and consent form prior to the recording. Participants returned written consent via the electronic consent form; this was also confirmed verbally at the start of each interview. Interviews were conducted by GF, PC, AK, JAG and ABK. Authors are educationalists (GF, PC, AK, JAG, PAT), clinicians (ET,JM, PT, JAG) and qualitative researchers (all). The researchers hold a range of qualifications including PhD, MD, MBChB, MMEd, PGDipMEd, MRCpsych, FRCP, FAcadMEd, FRCPsych, and MSc. The research team consisted of clinicians and non-clinicians, clinical academics at varying stages, expert qualitative researchers to novices, females and males, and a mix of ethnicities.

Data analysis

Data were thematically analysed [13, 14] using reflexive thematic analysis within NVivo (Version 10. QSR International; 2012). Coding was undertaken by GF, PC, AK, JAG, ABK and ET. Thematic analysis was chosen because it is an appropriate method for seeking to understand experiences, thoughts, or behaviours across a dataset [15]. The authors read the data in its entirety and developed a codebook as a tool to assist in the analysis of the large dataset [16]. The six-step process of thematic analysis was followed: (1) data familiarisation, (2) generating initial codes, (3) searching for themes, (4) reviewing themes, (5) defining and naming themes, and (6) producing the report.[14, 17] Both inductive and deductive approaches were taken, with deductive analysis based on existing theory including maternal wall bias, feminist theory and intersectionality.[18-20] A full coding tree can be accessed in a funder report[10]. Authors engaged in a process of negotiation to refine codes and themes, before utilising member checking with a subset of participants. Authors were reflexive, recording reflexive journals and acknowledging their biases and presuppositions. Team meetings were regularly held to discuss reflexivity. Since the study data were confined to a limited collection period during the pandemic, it was felt that this would not be of sufficient duration for meaningful temporal analysis. Instead researchers interpreted the data as being snapshots and in the moment reflections relating to the impact of the pandemic. In order to bring participants' experiences over time, cross-sectional themes together and demonstrate intersectionality case studies from two participants are provided (Box 1). Authors collected data until such time that they felt meaningful analysis and interpretation had been completed – in line with contemporary literature[21-23], we do not subscribe to the notion of saturation but rather took a pragmatic approach in collecting data to answer the question, within the constraint of time and resource. Further, we noted that information power[23].

For peer review only

Results

Demographics

Eighty-two individuals participated, 30 provided audio diary data, 68 were interviewed, while 16 provided both. Table 1 provides an overview of the descriptive statistics of the participant sample and the data collected. The research team received 134 diary entries, with a mean of 5 entries per participant. No participants withdrew or refused to participate.

TABLE 1: Demographic breakdown of participants

	Audio Diaries		Interviews	
**Profession**	**Total (n=30)**	**%**	**Total (n=68)**	**%**
Medicine	24	80.0	57	83.8
Dentistry	6	20.0	11	16.2
**Mean Age**	39		40	
**Age Range**	27 - 74		28 - 51	
**Gender**				
Male	10	33.3	28	41.2
Female	20	66.6	40	58.8
**Predominant Clinical Work Area**				
Primary	7	23.3	19	27.9
Secondary	11	36.7	30	44.1
Tertiary	12	40.0	17	25.0
Did not disclose	0	0	1	1.5
**Employment Status (overall)**				
Full Time	24	80.0	65	95.6
<Full Time	6	20.0	3	4.4
Did not disclose	0	0	0	0.0
**% of hours spent on academic work**				
100%	5	16.7	8	11.8
50%	19	63.3	3	4.4
<50%	6	20.0	57	83.8
Did not disclose	0	0	0	0.0
**Out of programme for research**				
No	19	63.3	55	80.9
Yes	5	16.7	3	4.4
Not applicable	6	20.0	10	14.7
**Ethnicity**				
Asian	2	6.7	11	16.2
Indian	2	6.7	3	4.4

For peer review only

Middle Eastern	1	3.3	2	2.9
Black	0	0	3	4.4
White Caucasian	24	80.0	47	69.1
Did not disclose	1	3.3	2	2.9
**Marital Status**				
Divorced	2	7.0	0	0.0
Long-term relationship (not married)	2	7.0	7	10.3
Married	23	77.0	56	82.4
Single	3	10.0	4	5.9
Did not disclose	0	0	1	1.5
**Sexuality**				
LGBTQIA+	1	3.3	4	5.9
Heterosexual	25	83.3	62	91.2
Did not disclose	4	13.3	2	2.9
**Disability**				
No	28	93.3	67	98.5
Yes	2	6.7	1	1.5
**Number of Children/Dependents**				
0	7	23.3	27	39.7
1	6	20.0	11	16.2
2	11	36.7	25	36.8
3	4	13.3	2	2.9
4	2	6.7	1	1.5
Did not disclose	0	0	2	2.9
**Pregnant**				
Yes	0	0	1	1.5
Did not disclose	1	3.3	7	10.3
No	29	96.7	60	88.2
**Current Clinical Academic Career Level**				
Doctoral Fellow/ PhD student	10	33.3	13	19.1
Academic Clinical Fellow	6	20.0	14	20.6
Academic Clinical Lecturer	6	20.0	14	20.6
Senior Clinical	6	20.0	27	39.7

For peer review only

Lecturer and above (including Deans and Programme Directors)				
Did not disclose	2	6.7	0	0.0
**Current grade within Clinical Role**				
Clinical Fellow	3	10.0	1	1.5
Registrar (Medical / Dental)	16	53.3	22	32.4
General Practitioner (Medical / Dental)	4	13.3	6	8.8
Medical / Dental consultant	6	20.0	30	44.1
Medical researcher	1	3.3	0	0.0
Did not disclose	0	0	1	1.5
**Location**				
East of England	1	3.3	2	2.9
Midlands	4	13.3	11	16.2
North East England & Yorkshire	11	36.7	26	38.2
North West of England	2	6.7	4	5.9
South East of England	6	20	16	23.5
South England	4	13.3	2	2.9
Wales	2	6.7	1	1.5
Scotland	0	0	5	7.4
Did not disclose	0	0	1	1.5
**Place primary health qualification awarded**				
UK	30	100	64	94.1
IMG	0	0	2	2.9
EEU	0	0	1	1.5
Did not disclose	0	0	1	1.5

For peer review only

**Total Number of Diary Entries**	134	N/A
**Number of Written Entries**	26	N/A

Themes

The analysis revealed four major themes, each with subthemes (Table 2), relating to the initial impact of COVID-19 on clinical academic careers; (1) opportunities, (2) barriers, (3) personal characteristics and social identity and (4) fears and uncertainty. Intersectionality and the differential impact of ethnicity and gender on the experiences were noted across all themes.

Due to the extensive dataset, only selected themes are presented in continuous prose; these have been chosen due to their pertinence to the research.
The analysis found the COVID-19 experiences of medical and dental clinical academics relatively similar, differences were noted when protected characteristics such as gender and ethnicity were considered.

Table 2: Themes and subthemes from audio diary entries

For peer review only

<table>
<tr><th>Opportunities</th><th>Barriers</th><th>Personal characteristics and social identity</th><th>Fears and Uncertainty</th></tr>
<tr><td colspan="4">⇐ ⇐ ⇐ Intersectionality ⇒ ⇒ ⇒
The impact of gender, ethnicity and associated intersectional identities were pertinent across all themes</td></tr>
<tr><td>New emerging opportunities
• New research avenues
• New collaborations
• COVID offers a new lens through which to think about existing research
• Increased capacity & opportunity to write</td><td rowspan="2">Lockdown
• Working at home with children
• Work life balance
• Parenting responsibilities
• Positive discrimination
• Gender roles
• Impact on physical & mental health from working at home
• Difficulty maintaining momentum
• Decline in academic outputs
• Isolation
• International work difficult
• Physical barriers impacting on work (e.g PPE, remote consultations)
• Demands from teaching responsibilities
• Loss of resource</td><td>Being a clinical academic
• Increased perceived importance of clinical academics
• Sitting in neither camp</td><td>Returning to the frontline
• Responding to the call to arms
• Contracting COVID-19
• BAME ethnicities increased susceptibility
• Anxiety
• Appropriateness of skillset for return to clinical duty
• Inability in short-term to conduct research
• Impact on long-term research career</td></tr>
<tr><td>Support
• Funder support (extensions & network)
• Supportive networks (local, national, international)
• Provision of childcare for keyworkers
• Camaraderie</td><td>Protected characteristics
• BAME
• Maternal status
• Gender stereotypes
• Intersectional identities</td><td>Misconceptions and absence information
• Funding
• Extensions
• Lockdown restrictions being lifted
• Fiscal impact and predicted recession
• Portfolio development due to loss of research capacity
• Future impact of COVID
• Uncertain job market</td></tr>
</table>

For peer review only

**Maintenance of normality**<br>• Job security<br>• Maintenance of structure<br>• Workload maintained	**Demand from teaching responsibilities**<br>• Switch to online teaching<br>• Lack of support for teaching activity<br>• Teaching is devalued	
**Technological advances supporting remote working**<br>• Maintenance of networks<br>• Inclusive approach to working	**Loss of resource**<br>• Loss of outputs<br>• Fiscal barriers<br>• Loss of research team & associated HR issues<br>• Decrease in academic capacity due to clinical workload<br>• Lack of research supervision<br>• Practical barriers (e.g. data storage)<br>• Absence of pastoral support	

For peer review only

Theme 1: Opportunities and Enablers

The pandemic presented many opportunities and enablers. These spanned academic and clinical work as well as participants' personal lives. Subthemes broadly fell into three categories; 1) new opportunities for research, 2) support, 3) maintenance of normality and 4) technological advances supporting remote working.

New Emerging Opportunities

Participants recognised the immediate opportunity to explore new avenues of research related to the pandemic.

> "*So I've been asked to, and volunteered to, be part of the Research Ethics Committee Coronavirus Response which means that every week now I've had twenty-four hour turnaround for a coronavirus study of some sort or another … I have been involved with developing two national and one international coronavirus studies over this time…an enormously exciting but terrifying roll out of research…*" [Male Medic - diary]

> "*…there might be a positive if you were, you know, an Oxford University clinician looking at vaccine so you'd obviously you get a lot of funding and, you know, good opportunity to, to research Covid based things… I suppose if you've never done a clinical trial before and you were a clinical academic, getting involved in some of the trials that they're doing at the moment, like the Recovery Trial, that could be a positive for you if you're in that particular situation…" [*Female Medic – interview*]*

Associated with this was the need for rapid research dissemination, providing clinical academics with a chance to develop their portfolio. This resulted in the forging of new research collaborations supported by the quick turnaround of ethical approval in order to roll out impactful research. More senior clinical academics reported opportunities for career development such as being invited to serve on Research Ethics Committees. In addition to the new research territory, COVID-19 presented a new lens through which participants were able to think about their existing research.

For some, salient amongst the opportunities was the free time which was created during the pandemic, particularly due to the absence of social commitments and commuting. Some clinical academics found this spare time to be an opportunity to focus on academic research activities and the production of impactful research. Others felt this was as an opportunity to focus on clinical work, as well as family and household commitments.

> **"***I guess it will give me a chance to focus on, you know, having a bit more time at home with family and, and just doing clinical work, which I think, you know, is what I wanted to focus on for the moment.*" [Female Medic – Audio Diary]

Support

Participants presented examples of the positive impact of support networks locally, nationally and internationally. The abrupt emergence of COVID and subsequent lockdown meant that many clinical academics halted their academic work and returned to clinical practice. This created an element of uncertainty, and in some cases anxiety about completing their academic work. However, a widely circulated statement

For peer review only

from the UK's main funding bodies reassuring clinical academics that they would support the extension of research post-COVID was well received.

> "*The [funder] have said that they will support extensions if they're needed so I do have that to kind of fall back on*." [Female Medic – Audio Diary]

An immense amount of support was provided by academic organizations at the beginning of the pandemic. Support from supervisors, departments and funding bodies was identified as enabling and empowering for participants. For example, the provision of childcare nurseries, enabling clinical academics to return to full-time clinical practice when required.

Maintenance of Normality

'Maintaining the status quo' during the pandemic was an enabler for many. From the beginning of the pandemic many professions have seen large proportions of redundancy. However, this has not been the case for many of the clinical academics that participated in this study. Clinical academics who have had the flexibility of undertaking either clinical or academic work during the pandemic, were grateful and felt generally lucky to have a job during COVID and one they could go back to post-COVID. Opportunities that strengthened the resolve of participants included the option to flex hours between roles. Specifically, it was also noted that going back to clinical practice full-time was perceived as advantageous due to more structure compared to academic work. Additionally, the retention of social interaction, structure and workload was seen as an opportunity.

> "*In these times of COVID-19 some people have kind of lost their structure and workload, so I feel grateful that hasn't happened to me*." [Male Medic – Audio Diary]

Technological advances supporting remote working

Participants advocated the use of video-conferencing platforms, citing them as enablers to maintain networks and collaborations during the pandemic as well as the inclusivity afforded by digital spaces.

> *"…my colleagues have become much more able to work online using Microsoft Teams and there have been more meetings recorded and more webinars which meant that as a part-time person I've been able to attend some of the things that previously I used to enquire and they used to say that this is not recorded and it is not being broadcast, so that has been excellent." [Female Medic – Interview]*

<u>Theme 2: Barriers</u>

There were multifaceted barriers related to the direct consequences of the immediate impact, reactive and mid-term barriers, and longer-term implications. Three subthemes were identified; (1) lockdown, (2) demand of teaching responsibilities and, (3) loss of resource.

Lockdown

For peer review only

Most of the barriers described by participants related to the negative consequences of lockdown. A barrier evidenced by particularly emotive data was that of working at home. This entailed the need to balance the multiple commitments related to childcare, housekeeping, and productivity. There were far reaching consequences of the ways in which working from home impacted on participants' roles. Childcare was not seen as compatible with a clinical academic role. Both clinical (e.g. telephone clinics and clinical administration) and academic work was often taking place at home, leading to feelings of isolation.

> "*I've spent a lot of time doing very intensive blocks of clinical work and very intensive blocks of research, research policy into practice work…I realised it's a Thursday today, I realised I had worked for three weeks solid and I'd not had a full day off in that time and most of those days were ten or twelve hour days…and I have now worked a further eleven days, with the last four days being fourteen hour days, without a break*". [Male Medic- Interview]

Mental and physical health outcomes were described as a consequence of COVID, particularly due to the increased workload.

> "*Generally, the COVID experience has been exhausting on many levels; emotionally from the worry about the number of people who will die, worry about being redeployed, worry for my children's wellbeing.*" [Male Medic- Interview]

> *"My anxiety is elevated due to being Asian, will I get sick and lose time for research?"* [Female Medic-Interview]

Concerns arose over how to effectively manage competing time demands. Some also spoke about a loss of 'downtime' and feeling 'stuck in limbo'. The shift in work-time meant that participants had less time for recreational activities which impacted on wellbeing.

Both males and females were impacted by the move to home working and balancing childcare with full-time work. The time pressures were apparent in many situations where participants struggled to navigate competing demands.

> "*… the Pro Vice Chancellor had sent an email saying how exciting it was that there's a female President at the Medical School, a female Dean, a female Pro Vice Chancellor of Medicine and a female Vice Chancellor at University, and that made me reflect what would happen if I sent a similar email delighting the fact that there's males in all of those roles. I imagine there'd be some disdain… they're powerful and take revenge…I don't think that's appropriate.*" [Male Medic – Audio Diary]

There were also concerns over positive discrimination in how gender roles were recognised, with a particular focus on senior positions. Such comments alluded to notions of male fragility and toxic masculinity, although the intent cannot be stated in absolute terms.

> *"…I do sometimes think that even, males might be at a, particularly at the end of, top end of their career, might be at more, slight disadvantage because, particularly with Athena SWAN there's obviously an emphasis, and maybe rightly so, in supporting women, you know, particularly take senior posts like chairs and therefore I do think sometimes it's almost got the point where it's the first discrimination where men might be overlooked, you know, because there's a keenness to promote women, and I, in a sense I understand the rationale for that, and it might get worse with the pandemic."* [Male medic-interview].

> *"it's kind of bordering on positive discrimination around research professorships, I think men are needed…"* [Male medic- interview].

Demands from teaching responsibilities

The analysis identified the negative impact of having to rapidly produce teaching and assessment materials for online delivery, frequently coupled with a lack of support and appreciation, as well as the time consumed. In many cases, this was in addition to clinical responsibilities.

> "*As clinical academics we are scavenging time in between the clinical parts to crack on and do our academic work*." [Male Medic – Audio Diary]

There was worry over a future lack of recognition within portfolios for teaching activities undertaken, and a frustration over negotiating the various new technologies required to deliver teaching.

Loss of resource

The impact of COVID has been significant for participants and extends from fiscal and time to human resource issues. There were concerns over the loss of time in relation to academic outputs and not fulfilling previously arranged activities. A lack of overall supervision was noted, including much needed pastoral support.

> "*There is an absence of pastoral support. Supervision is different, it's about skills and development not always about just how you feel. Sometimes you need a shoulder to cry on*". [Female Medic – Audio Diary]

Given the measures in the academic field where outputs are often used as a productivity marker, the participants were unsure how they could compensate loss of time and the ways in which their activities could be demonstrated and valued.

Tangible barriers were highlighted in the monetary implications for how loss of time could be compensated. Participants made calls for funders to extend time and funding for projects that otherwise may have been completed on time.

> "*It's bloody terrifying when it comes to applying for new stuff though because the deadlines haven't really moved much and there's no head space and there's no time to get the stuff really done properly and that's really scary but we've got to get the clinical work done first. It's the only way*." [Male Medic – Audio Diary]

Many universities initiated a recruitment freeze which meant in some cases that methodological expertise was lost. As clinical academics are often on a set educational employment path for a discrete time period this created disruption to long-term career plans.

Theme 3: Personal Characteristics and Social Identity

A key theme revealed was the impact of COVID-19 on participants' personal characteristics and social identity. This theme specifically considers how individuals perceived themselves as being clinical academics and the issues associated with the label 'clinical academic'.

Ambivalence and identity of a clinical academic

Some participants were conflicted about their identity as clinical academics and often experienced 'imposter syndrome'. Imposter syndrome, as described by the participants, refers to a feeling that they were not adequately qualified to be called clinical academics. Others expressed pride in being classed as clinical academics, however, they expressed that their identity as clinical academics was misunderstood by clinical and academic colleagues. Participants described problems experienced with negotiating both clinical and academic identities during the pandemic.

> "*Trying to tussle where you fit between university and clinical world at the moment is, is also hard*." [Male Medic– Audio Diary]

Participants highlighted that in their working life they do not sit neatly in either category, which makes it difficult to identify as either an academic and/or a clinician. This feeling has been intensified by the pandemic. Most professionals have an educational background where they have developed peer support and networks allowing for guidance, but for clinical academics, their varied pathways means they often lack this important support network.

Protected characteristics

Protected characteristics of clinical academics, particularly the BAME community who have been more widely affected by COVID-19, was a key subtheme identified. Increased anxiety was highlighted and participants who were from BAME backgrounds expressed fear about returning to clinical work. Muslim clinical academics also expressed the difficulty they faced due to Ramadan taking place during the already difficult time. The focus was largely on physical wellbeing due to heavy workload alongside being unable to eat or drink.

Gendered differences were evident throughout this study, with female participants specifically expressing the struggles of maternal identity, highlighting problems faced with childcare responsibilities as well as having to share these with their partners alongside work commitments.

> *"I'm not formally planning to carry on with academia and that's a decision that's kind of come about because of, you know, childcare responsibilities and I just*

For peer review only

felt that I was going to be stretched too thin if I was trying to be a mum and be a GP and be an academic as well." [Female Medic– Audio Diary]

Female participants felt that due to gender stereotypes from partners and work colleagues they were having to take a leading responsibility in childcare during this time.

"*I think that COVID has been a disaster for feminism...the disagreements that I've had with [husband] have been over feeling like a 1950s housewife suddenly then to suddenly home schooling children and trying to stay sane, stay safe, keep everybody okay, whereas he still is doing exactly what he does, work...*" [Female Medic– Audio Diary]

Whilst participants noted intersectionality, their narratives were mostly differentiating their experiences due to gender. Women explicitly stated that they became less tolerant of the gender issues raised as time in lockdown progressed.

Women reported the emotional turmoil associated with being primary caregivers.

"Yet another day of working from home while helping with young kids who are upset at the disruption to their lives. The most difficult thing is trying to calm my children's' worry over COVID." [Female Dentist– Audio Diary]

Increased perceived importance of clinical academics

Despite the tensions that have been caused by the pandemic, some noted that the work and identity of clinical academics have actually been strengthened. COVID has required rapid research to be undertaken and clinical academics are perfectly suited to this opportunity. The fact that qualified clinical academics have a foot in both research and clinical practice has facilitated a renewed appreciation of their identity during this pandemic.

Theme 4: Future fears and uncertainty

This theme related to numerous unanswered questions of participants including their fears and anxieties. Returning to the clinical environment was one major cause of anxiety, as well as the future of their research and funding.

Returning to the frontline

Discourse regarding a full return to clinical practice utilised many analogies to warfare. One source of internal conflict for participants was their inability to respond positively to the 'call to arms', particularly where they felt they had deskilled in some areas due to their focus on academia in recent years. Participants described requests to abandon research and assume clinical roles. Added to this anxiety was an inability to manage workload and family life.

"*Well all of the academic trainees have been pulled on to the full-time clinical work. As clinical academics we are scavenging time in between the clinical parts to crack on and do our academic work and certainly emotionally I'm finding it enormous strain, mainly because the clinical work itself has not so much increased in the intensity but the massive decrease in staff numbers, partly because of illness but a lot because of isolation and shielding*

For peer review only

requirements that have gone on, has hugely increased the amount of work that all of us that are at work are doing." [Male Medic– Audio Diary]

Additionally, the fear of contracting COVID-19 was expressed, with the majority of participants having to return to full clinical practice. CAs who expressed fear of contracting COVID-19 were those with predisposing factors and BAME backgrounds. Additionally, knowing someone who had died on the frontlines increased clinical academics' perception of their own risk.

Misconceptions and concerns about the future

From the onset of the pandemic, participants have reported concerns over their progression and future careers. Participants also aired concerns about the lockdown restrictions being lifted prematurely, stating that this could have further negative implications on their clinical and academic work. Uncertainty around COVID-19 has caused misconceptions, concerns and scaremongering, ranging from the prospect of more lockdowns to the potential for a return to fulltime clinical work only. There has also been an absence of concrete information for clinical academics. Amongst participants, there is a general fear and uncertainty about the fiscal impact on research funding and the future clinical academia.

"*Unfortunately my grant holders haven't sort of committed themselves to being able to extend my funding due to various reasons, so yeah, so there's uncertainty with that as well*." [Female Medic– Audio Diary]

Issues such as pay cuts and a loss of staffing resources within academic departments caused further anxieties.

"The news on pay cuts is a bitter pill to swallow when working in a department where teaching commitments have increased, I have to say. Clinicians may decide to switch to clinical only in some cases." [Male Medic– Audio Diary]

These anxieties have been further intensified following a lack of professional and social networking opportunities, especially as the pandemic intensified and lockdowns progressed.

Box 1: Case examples (CE) of the initial impact of COVID-19 on participants with intersecting identities (pseudonyms utilised).

Box 1: Case studies of the impact of COVID-19 (pseudonyms utilised).

Case study 1: Asian, Mother, Muslim, Medical Registrar/Doctoral Fellow

Zainab, a Medical Registrar in Neurology and Doctoral Fellow is a married, Asian, Muslim, mother of three young children. She is 'proudly intersectional'. Zainab works full time and spends about 90% of her time on her academic work. She has recently put her details down to be called to the frontlines and is currently on the standby list. As an Asian doctor, living and working in a city, she feels particularly vulnerable and has fears about her risks of contracting COVID. Her anxieties regarding the virus increased over time as

For peer review only

research linked BAME ethnicity to outcomes. She knows of a doctor who died on the front line, they were a relative of one of her closest friends. Although she realizes that susceptibility to the disease is multifactorial, she can see that people from minority ethnic groups are disproportionately affected. According to her, "*it's not known why [there's a disproportionately higher number] I think it is multifactorial whether there's underlying kind of health vulnerabilities that are more prevalent in certain ethnic groups that make them more vulnerable or whether there's more complex issues to that*." Despite the negative implications that the COVID pandemic and lockdown have brought, she finds a great deal of positives from the supportive academic institution.

Case study 2: White, Mother, Clinical Lecturer

Rose, a Medical Registrar and Academic Clinical Lecturer, has been working at home where she is married to a clinical professor with two children. Rose works part-time. She is struggling with the balance of family and work commitments, stating that lockdown has reinforced sexist gender roles. Rose feels that her research has taken a backseat while her husband's work has been prioritised, "*I genuinely think he thinks his work is more important, I genuinely think that's what he believes*." She reports a decline in mental health due to the pressures of lockdown and family dynamics, "*I think that COVID has been a disaster for feminism...the disagreements that I've had with [husband] have been over feeling like a 1950s housewife suddenly, then to suddenly have to provide home schooling children and trying to stay sane, stay safe, keep everybody okay, whereas he still is doing exactly what he does [work]...so I think that, that, that's my big worry is that actually COVID's been a step back*." Rose's anxiety about her research profile increased the longer lockdown restrictions were in place.

For peer review only

Discussion

This study presents a unique, qualitative data-driven reflection of the national clinical academic landscape during nine months of the COVID-19 pandemic. The in-depth interview data are supported by real time audio diaries. This multi-faceted approach has facilitated access to “periods of change and flux” particularly during the pandemic, and allowed the research team to capture the impact of COVID-19 in real-time which facilitated accurate data capture [24, 25]. The audio diaries are also advantageous owing to their ease of completion, which typically results in lower levels of attrition. [25] Participants were also permitted to submit written entries, but this was not deemed to be a limitation, especially as discourse analysis was not planned. Permitting flexibility also prevents diary entries from becoming formulaic [25]. Whilst the authors recognize that there is often a self-selection bias associated with studies of this nature, participants were felt to be balanced in their presentation of barriers and enablers [26]. Audio diaries varied in length and thus depth, however, there was no variability in the richness of data.

Clinical academics were impacted by COVID-19 in multiple ways leading to a wide range of barriers, challenges and opportunities underpinned by their personal and professional identities. The selflessness and dedication of the study participants to contribute to the clinical response to the COVID-19 pandemic is evident in the data. Clinical academics returning to the NHS during the pandemic, had added value with respect to their research experience[27].

Our findings identified the numerous perceived barriers to continuing academic activity within the family, academic and clinical contexts. What is clear is that pre-existing barriers to academic activity have become magnified during the COVID-19 pandemic. Although such barriers are not insurmountable, they have proved and continue to prove stressful for the participants. The impact on their future career plans is already evident. The restrictions on face-to-face contact, international travel, uncertainties over clinical and academic training and funding extensions, home working, and, in many cases, redeployment to frontline clinical duties have all impacted on academic activities. Tension was evident when academic trainees who switched to full time clinical work and sacrificed their academic time described how they felt disadvantaged in comparison to trainees who have been able to maintain research.

Female participants described barriers that related to their gender, as well as for many, their maternal status, with such biases having been previously well documented in the literature [18, 20, 28-30]. Our findings in the themes (2 and 3) and case studies, suggest that the pandemic and resultant lockdown has, in many cases, exacerbated the discrimination faced by many women in clinical medical and dental academia. A survey of dental clinical academics in 2018, found that overall female representation in the academic grades in Dentistry is increasing - mirroring gender representation in dentistry as a whole [31]. Given our data, it is possible that the COVID-19 pandemic could have an even more significant negative impact on female Dental Clinical Academics going forward.

Theme three also identified a number of females in our sample who described feeling and being compelled to assume, to a greater degree than before the pandemic, gender stereotypical roles in the home. Specifically, when faced with competing responsibilities females appeared to feel under more pressure than their male partners to allocate more time to childcare and household tasks. Indeed, there was a recurrent motif of narratives relating to the '1950s housewife' stereotype within the family context [32]. In this regard, our findings are consistent with media accounts that the lockdown has placed a disproportionate burden on females, who frequently report taking on the bulk of the childcare, domestic duties and home-based education of school-aged children [33, 34]. It has been postulated that many females are currently doing 'second and third shifts' with regard to housework and welfare after their first shift of childcare or paid work [32]. Prior to COVID-19, there was evidence that parental working determined that, on average, females spent two to three times as much time on care and housework compared to men [35]. Our findings suggest that these effects have become more pronounced as a result of the pandemic and are impacting significantly on female clinical academics. Our data may go some way towards explaining reported declines in publications by female academics [36].

The findings raise an alarm that prolonged periods of lockdown could reverse general trends towards a more equitable distribution of household labour [35]. More concerningly, it may substantially put back efforts to achieve a more gender representative body of clinical academics, characterised by gross under representation of female, explored by a recent systematic review [9]. This has been shown to not merely be a matter of social equality; the male dominance of science in general has served women's health poorly [37].

Of note within our data was the indication from male participants that they were experiencing positive discrimination. We would not wish to overinterpret, however, our analysis of the participants' narratives alluded to some potential degree of toxic masculinity [38] and fragility. In women's continued fight for equality within the workplace can often lead to discomfort from those who have typically held most privilege within the discussion. Although the discomfort articulated from males in our sample was a minority, it was expressed with conviction, especially in terms of positive discrimination.

There are a number of steps that could be taken to mitigate the impact of the COVID-19 pandemic on clinical academia as a whole and the risk of reduced long-term clinical research capacity, especially the loss of female academics. Funders and employers could intentionally target support at those with caring responsibilities during lockdown and the following recovery period. As the pressing clinical need recedes then prioritising the return of female academics to their research activities may also offset the bias more generally experienced by female scientists both before and during the pandemic. Whilst virtual networks of collaborators have organically sprung up in places during the lockdown, more of these could be strategically formed to counter the isolation felt by many academics. This support should also extend to academics who are primarily clinical educators, and have historically felt marginalised and undervalued [39]. Indeed, some of the participants in the present study voiced perceptions of a lack of support and appreciation from both their academic and clinical employers, and colleagues. Both existing and trainee clinical

For peer review only

academics reported a perception that neither non-academic clinical colleagues or non-clinical academics fully appreciated the competencies and the time demands of their academic roles. This perception has been reinforced by the COVID-19 pandemic. Going forward, enhanced collaboration and empathy between University and Clinical staff and employers could be targeted to remedy this.

As the UK navigates its immediate response to COVID-19, it is important to consider the potential implications for those whose careers are most vulnerable and require additional nurturing. There needs to be increased flexibility in career pathways and expectations, along with a review of funding and support offered. Participants requested flexibility with existing funding to make changes to study protocols and costings to enable them to pursue opportunistic COVID related research. Leaders in academic institutions and funding bodies must be cognisant of the varying degrees to which clinical academics will have been able to mitigate for COVID-19 in their assessments of applications for funding and positions and must revise their expectations for dissemination and impact. Causal explanations of the issues faced during COVID-19 will be multifaceted and sensitive to personal context. Teaching commitments must be valued and accounted for, as well as physical and mental health. It has been shown that clinical academics create national wealth as well as health [40]. We are heading into an inevitable global financial recession, if not depression, likely to also be characterised by an increase in mental and physical health needs which will undoubtedly impact on the clinical academic workforce and funding.

The infrastructure for medical and dental clinical academia is well established, yet our data suggest that better support is required to develop future clinical academic roles and enhance the provision of evidence-based patient care in the post-COVID-19 era. Participants report substantiated concerns over access to funding and positions once the status quo is resumed.

Whatever interventions and strategies are implemented, heed needs to be taken to the findings of Laver et al., who reported that interventions within clinical academia that require those they are designed to support, so called ‘bottom-up’ approaches, to bear the brunt of the workload are less likely to succeed [41]. Their review advocates the use of ‘top-down’ approaches, led by change in practice at a management level. Finally, their stance was that ‘something is better than nothing’.

Our findings offer further insight into the debate surrounding access to, and maintenance of, clinical academic careers [42]. We add weight to the evidence surrounding gender disparity. The well documented decline in the clinical academic workforce, the subject of contemporary research, [9, 43] could continue if strategies to support clinical academics are not implemented in the aftermath of the COVID-19 pandemic. Ongoing commitment to supporting and developing clinical academics is required, acknowledging the implicit biases facing females and parents in reaching their potential, and those with other competing demands on their time. Of paramount importance is the need for greater recognition of the value and contribution made by clinical academics to both the clinical and academic workforces to which they contribute. The reports from a recent national study by Finn and colleagues presented a number of proposed interventions to support clinical academics in the immediate wake of the pandemic, in addition to recommendations for ensuring the

long-term sustainability of the clinical academic pipeline. Headlines from qualitative data included the need for mentorship, flexibility and transparent processes, as well as recognition of the differential experiences of marginalised groups[10].

Our findings also emphasise the need for guidance for aspiring clinical academics. As one remarked, "I just need guidance on next steps. It is just so challenging to think about who might help me." Furthermore, without urgent action COVID-19 has the potential to increase the gender divide seen within clinical academia, and as demonstrated within recent studies [40-42]. It is hoped that the narratives in this study initiate a dialog between clinical academics, funders and institutions to successfully navigate the clinical academic pathways moving forward. Support and mentoring may be key to ensuring the clinical academic pipeline.

Future research

Future research could include collecting quantitative data to track the career progression, publication rates and grant successes for CAs to look for patterns, trends and potential biases based upon their protected characteristics and availability of support or mentoring.

Declaration

The manuscript is an honest, accurate, and transparent account of the study being reported. There are no omissions or inaccuracies. Participants have engaged in member checking of results and are briefed of findings.

Funding

This publication presents independent research funded by Cancer Research UK (Award Number: C71037/A29824), The Academy of Medical Sciences, Health Education England, The Medical Research Council, The National Institute for Health Research (NIHR) and The Wellcome Trust. The views expressed are those of the author(s) and not necessarily those of The Academy of Medical Sciences, Cancer Research UK, Health Education England, The Medical Research Council, The National Institute for Health Research (NIHR), The Wellcome Trust or the Department of Health and Social Care.

Acknowledgements

The authors wish to thank all the clinical academics who participated in this study. The authors acknowledge the work of the wider research team involved in this large-scale research project and thank them for their contributions. The authors also appreciate the insight of the project steering group at the Clinical Academic Training Forum. Thanks go to 'Women in Academic Medicine', and the British Medical Association for assistance in recruiting participants.

Data availability statement

Deidentified transcripts would be made securely available to other researchers if a written case for access is made to the research team and permission is granted by the funders of the study. This should be sent directly to the corresponding author GF.

Author contributions

For peer review only

GF, PC, PT, MK, JM and JB conceived the study. GF, PC, PT, JB and JM obtained funding. ET, GF, JB, PC, ABK and AK collected data and conducted the analyses. GF, AK, JB, PT and PC recruited participants and drafted the manuscript. ABK and AK curated the data. All authors reviewed the final manuscript.

Competing interests

The authors declare no competing interests.

References

1. Brenner, A.M., *Supervising the tearful trainee: opportunities for professional growth and learning.* Academic psychiatry : the journal of the American Association of Directors of Psychiatric Residency Training and the Association for Academic Psychiatry, 2014. **38**(4): p. 497-501.
2. Hourston, G.J., *The impact of despecialisation and redeployment on surgical training in the midst of the COVID-19 pandemic.* International Journal of Surgery (London, England), 2020. **78**: p. 1.
3. Clinical Academic Training Forum, *Clinical Academic Training Forum: Subgroup to support returning clinical academics from the COVID-19 front line*. 2020.
4. Guarino, C.M. and V.M. Borden, *Faculty service loads and gender: Are women taking care of the academic family?* Research in Higher Education, 2017. **58**(6): p. 672-694.
5. Ranieri, V., et al., *Factors that influence career progression among postdoctoral clinical academics: a scoping review of the literature.* BMJ Open, 2016. **6**(10): p. e013523.
6. Flaherty, C. *No Room of One's Own: Early journal submission data suggest COVID-19 is tanking women's research productivity*. 2020 [cited 2020 21st April 2020]; Available from: https://www.insidehighered.com/news/2020/04/21/early-journal-submission-data-suggest-covid-19-tanking-womens-research-productivity.
7. Pinho-Gomes, A.-C., et al., *Where are the women? Gender inequalities in COVID-19 research authorship.* BMJ Global Health, 2020. **5**(7): p. e002922.
8. McEwan, J., *Engaging women in academic medicine in the UK: report of a workshop at the Association of Physicians Annual Meeting, 2 April 2009.* QJM: An International Journal of Medicine, 2010. **103**(9): p. 635-639.
9. Brown, J.V., et al., *From the sticky floor to the glass ceiling and everything in between: protocol for a systematic review of barriers and facilitators to clinical academic careers and interventions to address these, with a focus on gender inequality.* Systematic reviews, 2020. **9**(1): p. 26.
10. Finn GM, B.J., Evans C, Raine G, Stewart L, Uphoff E, Kehoe A, Buchanan J, Balogun-Katung A, Tiffin P, Taylor E, Crampton P, Morgan J., *From the sticky floor to the glass ceiling and everything in between: A systematic review and qualitative study focusing on gender inequalities in Clinical Academic careers. Final report. .* 2020.
11. Finn GM, B.J., Evans C, Raine G, Stewart L, Uphoff E, Kehoe A, Buchanan J, Balogun-Katung A, Tiffin P, Taylor E, Crampton P, Morgan J., *Inequalities in UK*

For peer review only

clinical academic careers: a systematic review and qualitative study. Short report. . 2020.
12. Gordon, L., et al., *Multiple and multidimensional transitions from trainee to trained doctor: a qualitative longitudinal study in the UK.* BMJ open, 2017. **7**(11).
13. Braun, U.K., et al., *The utility of reflective writing after a palliative care experience: Can we assess medical students' professionalism?* Journal of Palliative Medicine, 2013. **16**(11): p. 1342-1349.
14. Braun, V. and V. Clarke, *Using thematic analysis in psychology.* Qualitative research in psychology, 2006. **3**(2): p. 77-101.
15. Kiger, M.E. and L. Varpio, *Thematic analysis of qualitative data: AMEE Guide No. 131.* Medical Teacher, 2020: p. 1-9.
16. Roberts, K., A. Dowell, and J.-B. Nie, *Attempting rigour and replicability in thematic analysis of qualitative research data; a case study of codebook development.* BMC medical research methodology, 2019. **19**(1): p. 66.
17. Braun, V. and V. Clarke, *Successful qualitative research: A practical guide for beginners*. 2013: sage.
18. Williams, J.C. and N. Segal, *Beyond the maternal wall: Relief for family caregivers who are discriminated against on the job.* Harv. Women's LJ, 2003. **26**: p. 77.
19. Brown, M.E., et al., *'Too male, too pale, too stale': a qualitative exploration of student experiences of gender bias within medical education.* BMJ open, 2020. **10**(8): p. e039092.
20. Williams, J.C., *Hitting the maternal wall.* Academe, 2004. **90**(6): p. 16.
21. Braun, V. and V. Clarke, *To saturate or not to saturate? Questioning data saturation as a useful concept for thematic analysis and sample-size rationales.* Qualitative research in sport, exercise and health, 2021. **13**(2): p. 201-216.
22. Varpio, L., et al., *Shedding the cobra effect: problematising thematic emergence, triangulation, saturation and member checking.* Medical education, 2017. **51**(1): p. 40-50.
23. Malterud, K., V.D. Siersma, and A.D. Guassora, *Sample size in qualitative interview studies: guided by information power.* Qualitative health research, 2016. **26**(13): p. 1753-1760.
24. Monrouxe, L.V., *Negotiating professional identities: dominant and contesting narratives in medical students' longitudinal audio diaries.* Current Narratives, 2009. **1**(1): p. 41-59.
25. Crozier, S.E. and C.M. Cassell, *Methodological considerations in the use of audio diaries in work psychology: Adding to the qualitative toolkit.* Journal of occupational and organizational psychology, 2016. **89**(2): p. 396-419.
26. Collier, D. and J. Mahoney, *Insights and pitfalls: Selection bias in qualitative research.* World Politics, 1996. **49**(1): p. 56-91.
27. Rimmer, A., *Covid-19: How clinical academic trainees came back to the wards en masse.* bmj, 2021. **373**.
28. Crosby, F.J., J.C. Williams, and M. Biernat, *The maternal wall.* Journal of Social Issues, 2004. **60**(4): p. 675-682.
29. Williams, J.C., *The glass ceiling and the maternal wall in academia.* New Directions for Higher Education, 2005. **2005**(130): p. 91-105.
30. Halley, M.C., et al., *Physician mothers' experience of workplace discrimination: a qualitative analysis.* BMJ, 2018. **363**: p. k4926.
31. Council, D.S., *Professorial roles for dentists fall 8.3% since 2015.* British Dental Journal, 2018. **225**(5).

For peer review only

32. Chung, H. *Return of the 1950s housewife? How to stop coronavirus lockdown reinforcing sexist gender roles*. 2020; 21st April 2020]. Available from: https://theconversation.com/return-of-the-1950s-housewife-how-to-stop-coronavirus-lockdown-reinforcing-sexist-gender-roles-134851.
33. Ferguson, D., *'I feel like a 1950s housewife': how lockdown has exposed the gender divide*, in *The Guardian*. 2020.
34. Alon, T.M., et al., *The impact of COVID-19 on gender equality*. 2020, National Bureau of Economic Research.
35. Wishart, R., et al., *Changing patterns in parental time use in the UK*. 2019.
36. Viglione, G., *Are women publishing less during the pandemic? Here's waht the data say*, in *Nature*. 2020. p. 365-366.
37. Burford, B., et al., *Professionalism education should reflect reality: findings from three health professions.* Medical Education, 2014. **48**(4): p. 361-374.
38. Harrington, C., *What is "toxic masculinity" and why does it matter?* Men and Masculinities, 2021. **24**(2): p. 345-352.
39. Kumar, K., C. Roberts, and J. Thistlethwaite, *Entering and navigating academic medicine: academic clinician-educators' experiences.* Medical education, 2011. **45**(5): p. 497-503.
40. Innovation, B. and G. Team, *Bioscience 2015: Improving National Health.* Increasing National Wealth, 2003.
41. Laver, K.E., et al., *A systematic review of interventions to support the careers of women in academic medicine and other disciplines.* BMJ open, 2018. **8**(3).
42. Rothwell, P.M., *Medical academia is failing patients and clinicians*. 2006, British Medical Journal Publishing Group.
43. Fitzpatrick, S., *A survey of staffing levels of medical clinical academics in UK medical schools as at 31 July 2011.* London: Medical Schools Council, 2012.

For peer review only

Reference:

Tong A, Sainsbury P, Craig J. Consolidated criteria for reporting qualitative research (COREQ): a 32-item checklist for interviews and focus groups. International Journal for Quality in Health Care. 2007. Volume 19, Number 6: pp. 349 – 357

Consolidated criteria for reporting qualitative studies (COREQ): 32-item checklist

No	Item	Guide questions/description
		**Domain 1: Research team and reflexivity**
Personal Characteristics		
1.	Interviewer/facilitator	Which author/s conducted the interview or focus group?<br>GF, MK, PC, JAG, AB<br>Page 5
2.	Credentials	What were the researcher's credentials? *E.g. PhD, MD*<br>The researchers had a range of qualifications including PhD, MD, MBChB, MMEd, PGDipMEd, MRCpsych, FRCP, FAcadMEd, FRCPsych, and MSc.<br>Page 5
3.	Occupation	What was their occupation at the time of the study?<br>GF was Professor of Medical Education, PC was a lecturer, MK and AB were Research Fellows, ET was a medical student. PAT, JM, CS and JAG were clinical academics.
4.	Gender	Was the researcher male or female?<br>Three were male, 6 were female<br>Page 5
5.	Experience and training	What experience or training did the researcher have?<br>All trained in research methods and well published in the field.<br>Page 5
Relationship with participants		

For peer review only - http://bmjopen.bmj.com/site/about/guidelines.xhtml

No	Item	Guide questions/description
6.	Relationship established	Was a relationship established prior to study commencement?<br><br>No<br>Page 4
7.	Participant knowledge of the interviewer	What did the participants know about the researcher? e.g. *personal goals, reasons for doing the research*<br><br>Participants were provided with an information sheet outlining the reasons for doing the research. They had the opportunity to ask questions and were able to access a study website and twitter account for information.<br><br>Page 4/5
8.	Interviewer characteristics	What characteristics were reported about the interviewer/facilitator? e.g. *Bias, assumptions, reasons and interests in the research topic*<br><br>A reflexivity exercise was conducted to demonstrate the researchers' relationship to the study findings. This is provided on the project website.<br><br>Page 5
		**Domain 2: study design**
Theoretical framework		
9.	Methodological orientation and Theory	What methodological orientation was stated to underpin the study? *e.g. grounded theory, discourse analysis, ethnography, phenomenology, content analysis*<br><br>As stated on page 5of the methods section, the methodological orientation underpinning this study is reflexive thematic analysis
Participant selection		

For peer review only

No	Item	Guide questions/description
10.	Sampling	How were participants selected? *e.g. purposive, convenience, consecutive, snowball*<br>As stated on page 4, participants were selected using purposive sampling with snowballing<br>Page 4
11.	Method of approach	How were participants approached? e.*g. face-to-face, telephone, mail, email*<br>As stated on page 4, participants were recruited through social media, email listserv, referrals
12.	Sample size	How many participants were in the study?<br>As stated on page 6, there were 104 participants in the main dataset
13.	Non-participation	How many people refused to participate or dropped out? Reasons?<br><br>As stated on page 6 of the methods section, no (0) people refused to participate or dropped out from our study
Setting		
14.	Setting of data collection	Where was the data collected? e.*g. home, clinic, workplace*<br><br>As stated in the methods section on pages 4-5, data were collected using telephone interviews and audio diaries
15.	Presence of non-participants	Was anyone else present besides the participants and researchers?<br><br>No – see page 4
16.	Description of sample	What are the important characteristics of the sample? *e.g. demographic data, date*<br><br>As stated on page 4, the sample was made up of clinical academics in the UK
Data collection		
17.	Interview guide	Were questions, prompts, guides provided by the authors? Was it pilot tested?<br><br>As stated on page 4, and within the appendix, guidance was provided for audio diaries, interviews used piloted stems

For peer review only - http://bmjopen.bmj.com/site/about/guidelines.xhtml

No	Item	Guide questions/description
18.	Repeat interviews	Were repeat interviews carried out? If yes, how many?<br><br>As stated on page 4 no repeat interviews were carried out
19.	Audio/visual recording	Did the research use audio or visual recording to collect the data?<br><br>As stated on page 4 the research used audio recording to collect data
20.	Field notes	Were field notes made during and/or after the interview or focus group?<br><br>Yes, as stated on page, making of field notes was an ongoing process (during and after interviews), as part of reflexivity
21.	Duration	What was the duration of the interviews or focus group?<br>As stated on page…, the interviews lasted between 45 to 60 mins
22.	Data saturation	Was data saturation discussed?<br>Yes, the authors discussed data saturation on page 5.
23.	Transcripts returned	Were transcripts returned to participants for comment and/or correction?<br>As stated on page 5, transcripts were returned to participants for comment and/or correction upon request
		**Domain 3: analysis and findings**
Data analysis		
24.	Number of data coders	How many data coders coded the data?<br>As stated on page 4/5, 5 researchers coded the data
25.	Description of the coding tree	Did authors provide a description of the coding tree?<br>As stated on page 5, this description is listed on our website.
26.	Derivation of themes	Were themes identified in advance or derived from the data?

For peer review only - http://bmjopen.bmj.com/site/about/guidelines.xhtml

No	Item	Guide questions/description
		As detailed on page 5 themes were identified using a mixture of deductive and inductive methods
27.	Software	What software, if applicable, was used to manage the data?<br>As stated on page 5 NVivo was used manage the qualitative data
28.	Participant checking	Did participants provide feedback on the findings?<br>Yes, this is stated on page 5.
Reporting		
29.	Quotations presented	Were participant quotations presented to illustrate the themes / findings? Was each quotation identified? e.*g. participant number*<br><br>Yes participant quotations were presented throughout the RESULTS section to illustrated the associated themes
30.	Data and findings consistent	Was there consistency between the data presented and the findings?<br><br>Yes, this is stated on within the results and discussion sectons.
31.	Clarity of major themes	Were major themes clearly presented in the findings?<br><br>Yes, the themes are provided in the table 2.
32.	Clarity of minor themes	Is there a description of diverse cases or discussion of minor themes?<br><br>Yes, this description is provided in the discussion section (page 19 onwards)

For peer review only

For peer review only - http://bmjopen.bmj.com/site/about/guidelines.xhtml

BMJ Open

The impact of the COVID-19 pandemic on the research activity and working experience of clinical academics, with a focus on gender and ethnicity: a qualitative study in the UK

Journal:	*BMJ Open*
Manuscript ID	bmjopen-2021-057655.R3
Article Type:	Original research
Date Submitted by the Author:	23-May-2022
Complete List of Authors:	Finn, Gabrielle; The University of Manchester, Division of Medical Education, School of Medical Sciences, Faculty of Biology, Medicine and Health<br>Crampton, Paul; Hull York Medical School, Health Professions Education Unit<br>Buchanan , John; Barts and The London School of Medicine and Dentistry, Centre for Education and Innovation<br>Balogun, Abisola Olatokunbo; Hull York Medical School, Health Professions Education Unit<br>Tiffin, Paul; University of York, Health Sciences<br>Morgan, Jessica; University of York, Centre for Reviews and Dissemination; Leeds Teaching Hospitals NHS Trust, Department of Paediatric Oncology<br>Taylor , Ellie; Hull York Medical School, Health Professions Education Unit<br>Soto, Carmen; British Medical Association<br>Kehoe, Amelia; Hull York Medical School, Health Professions Education Unit
**Primary Subject Heading**:	Qualitative research
Secondary Subject Heading:	Medical education and training, Qualitative research, Research methods, Public health
Keywords:	COVID-19, QUALITATIVE RESEARCH, MEDICAL EDUCATION & TRAINING

SCHOLARONE™
Manuscripts

I, the Submitting Author has the right to grant and does grant on behalf of all authors of the Work (as defined in the below author licence), an exclusive licence and/or a non-exclusive licence for contributions from authors who are: i) UK Crown employees; ii) where BMJ has agreed a CC-BY licence shall apply, and/or iii) in accordance with the terms applicable for US Federal Government officers or employees acting as part of their official duties; on a worldwide, perpetual, irrevocable, royalty-free basis to BMJ Publishing Group Ltd ("BMJ") its licensees and where the relevant Journal is co-owned by BMJ to the co-owners of the Journal, to publish the Work in this journal and any other BMJ products and to exploit all rights, as set out in our licence.

The Submitting Author accepts and understands that any supply made under these terms is made by BMJ to the Submitting Author unless you are acting as an employee on behalf of your employer or a postgraduate student of an affiliated institution which is paying any applicable article publishing charge ("APC") for Open Access articles. Where the Submitting Author wishes to make the Work available on an Open Access basis (and intends to pay the relevant APC), the terms of reuse of such Open Access shall be governed by a Creative Commons licence – details of these licences and which Creative Commons licence will apply to this Work are set out in our licence referred to above.

Other than as permitted in any relevant BMJ Author's Self Archiving Policies, I confirm this Work has not been accepted for publication elsewhere, is not being considered for publication elsewhere and does not duplicate material already published. I confirm all authors consent to publication of this Work and authorise the granting of this licence.

For peer review only

The impact of the COVID-19 pandemic on the research activity and working experience of clinical academics, with a focus on gender and ethnicity: a qualitative study in the UK

*Gabrielle M Finn[1], Paul E S Crampton[2], John Buchanan[3], Abisola Balogun-Katung[2], Paul A. Tiffin[4], Jessica E Morgan[5,6], Ellie Taylor[2], Carmen Soto[7], Amelia Kehoe[2]

Affiliations

1. Division of Medical Education, School of Medical Sciences, Faculty of Biology, Medicine and Health, The University of Manchester, M13 9NT
2. Health Professions Education Unit, Hull York Medical School, University of York, York, UK, YO10 5DD
3. Queen Mary University London, Barts Health NHS Trust
4. Mental Health and Addiction Research Group, Department of Health Sciences, University of York, York, UK, YO10 5DD
5. Centre for Reviews and Dissemination, University of York, York, UK, YO10 5DD
6. Department of Paediatric Oncology, Leeds Children's Hospital, Leeds, UK, LS1 3EX
7. British Medical Association, London, WC1H 9JP

Correspondence to:
Professor Gabrielle M. Finn
Division of Medical Education, School of Medical Sciences, Faculty of Biology, Medicine and Health, The University of Manchester, M13 9NT
gabrielle.finn@manchester.ac.uk
Twitter: @gabs_finn

Word count: 5712

For peer review only

Abstract

Objective: To investigate the impact of the COVID-19 pandemic on the research activity and working experience of clinical academics, with a focus on gender and ethnicity.

Design: Qualitative study based on interviews and audio/written diary data.

Setting: UK study within clinical academia.

Participants: Purposive sample of 82 clinical academics working in medicine and dentistry across all career stages ranging from academic clinical fellows and doctoral candidates to professors.

Methods: Qualitative semi-structured interviews (n=68) and audio diary data (n=30; including 16 participants who were also interviewed) collected over an eight-month period (January-September 2020), thematically analysed.

Results: 20 of 30 (66.6%) audio diary contributors and 40 of 68 (58.8%) interview participants were female. Of the participants who disclosed ethnicity, 5 of 29 (17.2%) audio diary contributors and 19/66 (28.8%) interview participants identified as Black, Asian or another minority (BAME). Four major themes were identified in relation to the initial impact of COVID-19 on clinical academics: opportunities, barriers, personal characteristics & social identity and fears & uncertainty. COVID-19 presented opportunities for new avenues of research. Barriers included access to resources to conduct research and the increasing teaching demands. One of the most prominent sub-themes within 'personal characteristics' was that of the perceived negative impact of the pandemic on the work of female clinical academics. This was attributed to inequalities experienced in relation to childcare provision and research capacity. Participants described differential experiences based upon their gender and ethnicity, noting intersectional identities.

Conclusions: While there have been some positives afforded to clinical academics, particularly for new avenues of research, COVID-19 has negatively impacted workload, future career intentions, and mental health. BAME academics were particularly fearful due to the differential impact on health. Our study elucidates the direct and systemic discrimination that creates barriers to women's career trajectories in clinical academia. A flexible, strategic response that supports clinical academics in resuming their training and research is required. Interventions are needed to mitigate the potential lasting impact on capacity from the pandemic, and the potential for the loss of women from this valuable workforce.

Strengths and limitations of this study

- Study utilised both audio and written diary entries to enable exploration of sensitive issues.
- Telephone and online interviews enabled data collection to continue during the pandemic.

For peer review only

- The research team was diverse in terms of gender, ethnicity and experience.
- The study was limited to the UK.
- The clinical academics were limited to doctors and dentists.

Introduction

The workforce presence of clinical academics is vital to healthcare to ensure that scientific knowledge underpins advances in medical education, translational research and patient outcomes. Their low number and lack of senior pipeline progression is alarming, particularly so for under-represented groups including women and ethnic minorities.[1] The COVID-19 pandemic has further exacerbated the situation through rapid redeployment, burnout, reinforcement of gender and racial stereotypes, and financial inequity.[2]

Over 1,500 academic trainees in England were estimated to be redeployed to clinical work during the COVID-19 pandemic[3]. This equates to more than 90% of those on the Integrated Academic Training (IAT) pathway.[3] Following the closure of high-risk aerosol-generating dental services, some junior hospital dentists and dental clinical academics were also redeployed to provide support care for hospital services.[4] The potential impact on trainees' clinical experience has been recognised.[5] In terms of formally monitoring and permitting advancement in postgraduate training, 'no fault' Annual Review of Competence Progression (ARCP) outcomes (10.1 and 10.2) have been introduced where acquisition of competencies has been delayed owing to COVID-19[6]. Meanwhile, there has been a research paradox whereby some research institutions have closed, while others have adapted and embraced COVID specific research [7]. Further issues highlight the consumption of academic resources that may prove ineffective or wasteful owing to haste, rapid redesign of online teaching materials, and inadequate infrastructure and design.[8] With the current prioritization of COVID-19 studies, new clinical trials have been paused [9] with detrimental impacts on clinical academics working on non-COVID research.

Prior to the pandemic, it was recognised that females in medicine tended to take on more 'service work' and had less protected time for research than their male counterparts.[4] In addition to low representation of minority groups, the lack of role models provides a further barrier. [5] Collectively, gender and racial stereotypes, childcare and home-working all put further pressure on an already stretched workforce, with women now publishing less.[6, 7] Losing further women and minority groups from this fragile workforce could run the risk of long-term reduced diversity in medical education, less female-relevant clinical research and ultimately have a negative impact on patient outcomes, in addition to having less women visible in senior positions [8, 9].

We sought to explore discrimination based upon protected characteristics in the clinical academic workforce, at various stages of the pipeline during the pandemic. We aimed to understand the barriers, enablers and interventions to facilitate clinical academic careers, thereby to develop key recommendations to support and reduce attrition of these exceptionally talented individuals. We were particularly interested in the experiences of women and clinical academics from different ethnic groups.

Methods

Ethics

Ethical approval for this study was obtained from the Hull York Medical School Ethics Committee (ref: 19 32).

Context

Prior to the COVID-19 pandemic, the authors were engaged in a multi-funder research project exploring barriers and facilitators to a career in UK clinical academia[10, 11]. As data were iteratively analysed, the impact of COVID-19 on clinical academia progressively shaped the experiences of the participants and relevant themes emerged. In order to assess the impact of COVID-19, the data collected after the first confirmed case within the UK (29th January 2020) until the end of September 2020 were isolated for analysis. This period included the first period of significant national restrictions ("lockdown") from 26th March to a phased reopening in June.

This study was conducted within a social constructivism orientation, additionally informed by the multiple principles of feminist theory. Feminist theory holds a constructivist ontology.

Patient and public involvement

The study had a steering group which included Patient and Public representation, provided by Health Watch.

Study design and recruitment

Qualitative interviews and longitudinal qualitative audio and written diary entries were utilised. Purposive sampling was undertaken, using social media, funder mailing lists and snowballing. Inclusion criteria specified participants were a doctor or dentist, based within the UK and working as or towards becoming a clinical academic. For the purposes of the study, a 'clinical academic' was defined by individuals themselves and how they perceived their roles. Recruitment was through various avenues; a website, emails sent on behalf of the research team by stakeholder organisations; social media recruitment; and 'snowballing'. Authors had no relationship with participants prior to the study.

Data collection

Data were collected using (1) audio and written diary entries, and (2) semi-structured interviews. Diaries enabled participants to discuss their views on a wide range of factors related to clinical academic careers, and latterly their experiences of working during COVID-19. The diary method enabled researchers to collect 'novel' and timely data during the transition to lockdown phase, a critical moment in healthcare. Many diaries were recorded immediately after events, such as discriminatory remarks being made in a workplace. Participants were invited to submit audio diary entries (MP3 files) via email or attached to an encrypted message. Submissions were sent to a professional account linked to the project following best practice.[12] In order to

For peer review only

promote engagement, participants were also permitted to write their diary entry.[12] Participants were able to submit diaries as frequently as they wished, each acknowledged by the researchers to maintain rapport and engagement. Participants were issued with a guide to follow which was informed by a concurrent systematic review.[9] The guide suggested frequencies of entries, instructions for submission and indicative content (Appendix 1). Reminder emails were sent monthly. Interviews were conducted via video conferencing or telephone with the use of a topic guide. Audio diary files and interviews were recorded and transcribed verbatim. All participants were given an electronic information sheet and consent form prior to the recording. Participants returned written consent via the electronic consent form; this was also confirmed verbally at the start of each interview. Interviews were conducted by GF, PC, AK, JAG and ABK. Authors are educationalists (GF, PC, AK, JAG, PAT), clinicians (ET,JM, PT, JAG) and qualitative researchers (all). The researchers hold a range of qualifications including PhD, MD, MBChB, MMEd, PGDipMEd, MRCpsych, FRCP, FAcadMEd, FRCPsych, and MSc. The research team consisted of clinicians and non-clinicians, clinical academics at varying stages, expert qualitative researchers to novices, females and males, and a mix of ethnicities.

Data analysis

Data were thematically analysed [13, 14] using reflexive thematic analysis within NVivo (Version 10. QSR International; 2012). Coding was undertaken by GF, PC, AK, JAG, ABK and ET. Thematic analysis was chosen because it is an appropriate method for seeking to understand experiences, thoughts, or behaviours across a dataset [15]. The authors read the data in its entirety and developed a codebook as a tool to assist in the analysis of the large dataset [16]. The six-step process of thematic analysis was followed: (1) data familiarisation, (2) generating initial codes, (3) searching for themes, (4) reviewing themes, (5) defining and naming themes, and (6) producing the report.[14, 17] Both inductive and deductive approaches were taken, with deductive analysis based on existing theory including maternal wall bias, feminist theory and intersectionality.[18-20] A full coding tree can be accessed in a funder report[10]. Authors engaged in a process of negotiation to refine codes and themes, before utilising member checking with a subset of participants. Authors were reflexive, recording reflexive journals and acknowledging their biases and presuppositions. Team meetings were regularly held to discuss reflexivity. Since the study data were confined to a limited collection period during the pandemic, it was felt that this would not be of sufficient duration for meaningful temporal analysis. Instead, researchers interpreted the data as being snapshots and in-the-moment reflections relating to the impact of the pandemic. In order to bring participants' experiences over time, cross-sectional themes together and demonstrate intersectionality case studies from two participants are provided (Box 1). Authors collected data until such time that they felt meaningful analysis and interpretation had been completed – in line with contemporary literature[21-23], we do not subscribe to the notion of saturation but rather took a pragmatic approach in collecting data to answer the question, within the constraint of time and resource. Further, we noted that information power[23].

Results

Demographics

Of 82 individual participants, 14 provided audio diary data only, 52 were interviewed only, and 16 were both interviewed and provided audio diary data. Table 1 provides an overview of the descriptive statistics of the participant sample and the data collected. The research team received 134 diary entries, with a mean of 5 entries per participant. No participants withdrew or refused to participate.

Table 1: Demographic breakdown of participants

	Audio diaries		Interviews	
**Profession**	**Total (n=30)**	**%**	**Total (n=68)**	**%**
Medicine	24	80.0	57	83.8
Dentistry	6	20.0	11	16.2
**Mean age**	39		40	
**Age range**	27 – 74		28 - 51	
**Gender**				
Male	10	33.3	28	41.2
Female	20	66.6	40	58.8
**Predominant clinical work area**				
Primary	7	23.3	19	27.9
Secondary	11	36.7	30	44.1
Tertiary	12	40.0	17	25.0
Did not disclose	0	0	1	1.5
**Employment status (overall)**				
Full Time	24	80.0	65	95.6
<Full Time	6	20.0	3	4.4
Did not disclose	0	0	0	0.0
**% of hours spent on academic work**				
100%	5	16.7	8	11.8
50%	19	63.3	3	4.4
<50%	6	20.0	57	83.8
Did not disclose	0	0	0	0.0
**Out of programme for research**				
No	19	63.3	55	80.9
Yes	5	16.7	3	4.4
Not applicable	6	20.0	10	14.7
**Ethnicity**				
Asian	2	6.7	11	16.2
Indian	2	6.7	3	4.4
Middle Eastern	1	3.3	2	2.9
Black	0	0	3	4.4

For peer review only

White Caucasian	24	80.0	47	69.1
Did not disclose	1	3.3	2	2.9
**Marital status**				
Divorced	2	7.0	0	0.0
Long-term relationship (not married)	2	7.0	7	10.3
Married	23	77.0	56	82.4
Single	3	10.0	4	5.9
Did not disclose	0	0	1	1.5
**Sexuality**				
LGBTQIA+	1	3.3	4	5.9
Heterosexual	25	83.3	62	91.2
Did not disclose	4	13.3	2	2.9
**Disability**				
No	28	93.3	67	98.5
Yes	2	6.7	1	1.5
**Number of children/dependents**				
0	7	23.3	27	39.7
1	6	20.0	11	16.2
2	11	36.7	25	36.8
3	4	13.3	2	2.9
4	2	6.7	1	1.5
Did not disclose	0	0	2	2.9
**Pregnant**				
Yes	0	0	1	1.5
Did not disclose	1	3.3	7	10.3
No	29	96.7	60	88.2
**Current clinical academic career level**				
Doctoral Fellow/ PhD student	10	33.3	13	19.1
Academic Clinical Fellow	6	20.0	14	20.6
Academic Clinical Lecturer	6	20.0	14	20.6
Senior Clinical Lecturer and above (including	6	20.0	27	39.7

For peer review only

Deans and Programme Directors)				
Did not disclose	2	6.7	0	0.0
**Current grade within clinical role**				
Clinical Fellow	3	10.0	1	1.5
Registrar (Medical / Dental)	16	53.3	22	32.4
General Practitioner (Medical / Dental)	4	13.3	6	8.8
Medical / Dental consultant	6	20.0	30	44.1
Medical researcher	1	3.3	0	0.0
Did not disclose	0	0	1	1.5
**Location**				
East of England	1	3.3	2	2.9
Midlands	4	13.3	11	16.2
North East England & Yorkshire	11	36.7	26	38.2
North West of England	2	6.7	4	5.9
South East of England	6	20	16	23.5
South England	4	13.3	2	2.9
Wales	2	6.7	1	1.5
Scotland	0	0	5	7.4
Did not disclose	0	0	1	1.5
**Place primary health qualification awarded**				
UK	30	100	64	94.1
IMG	0	0	2	2.9
EEU	0	0	1	1.5
Did not disclose	0	0	1	1.5
**Total number of diary entries**	134		N/A	

For peer review only

**Number of written entries**	26	N/A

Themes

The analysis revealed four major themes, each with subthemes (Table 2), relating to the initial impact of COVID-19 on clinical academic careers; (1) opportunities, (2) barriers, (3) personal characteristics and social identity and (4) fears and uncertainty. Intersectionality and the differential impact of ethnicity and gender on the experiences were noted across all themes.

Due to the extensive dataset, only selected themes are presented in continuous prose; these have been chosen due to their pertinence to the research.
The analysis found the COVID-19 experiences of medical and dental clinical academics relatively similar, differences were noted when protected characteristics such as gender and ethnicity were considered.

Opportunities	Barriers	Personal characteristics and social identity	Fears and uncertainty
⇐⇐⇐ **Intersectionality** ⇒⇒⇒<br>The impact of gender, ethnicity and associated intersectional identities were pertinent across all themes			
**New emerging opportunities**<br>• New research avenues<br>• New collaborations<br>• COVID offers a new lens through which to think about existing research<br>• Increased capacity & opportunity to write	**Lockdown**<br>• Working at home with children<br>• Work life balance<br>• Parenting responsibilities<br>• Positive discrimination<br>• Gender roles<br>• Impact on physical & mental health from working at home<br>• Difficulty maintaining momentum	**Being a clinical academic**<br>• Increased perceived importance of clinical academics<br>• Sitting in neither camp	**Returning to the frontline**<br>• Responding to the call to arms<br>• Contracting COVID-19<br>• BAME ethnicities increased susceptibility<br>• Anxiety<br>• Appropriateness of skillset for return to clinical duty<br>• Inability in short-term to conduct research<br>• Impact on long-term research career

Table 2: Themes and subthemes from audio diary entries

**Support**<br>• Funder support (extensions & network)<br>• Supportive networks (local, national, international)<br>• Provision of childcare for keyworkers<br>• Camaraderie	• Decline in academic outputs<br>• Isolation<br>• International work difficult<br>• Physical barriers impacting on work (e.g PPE, remote consultations)<br>• Demands from teaching responsibilities<br>• Loss of resource	**Protected characteristics**<br>• BAME<br>• Maternal status<br>• Gender stereotypes<br>• Intersectional identities	**Misconceptions and absence information**<br>• Funding<br>• Extensions<br>• Lockdown restrictions being lifted<br>• Fiscal impact and predicted recession<br>• Portfolio development due to loss of research capacity<br>• Future impact of COVID<br>• Uncertain job market
**Maintenance of normality**<br>• Job security<br>• Maintenance of structure<br>• Workload maintained	**Demand from teaching responsibilities**<br>• Switch to online teaching<br>• Lack of support for teaching activity<br>• Teaching is devalued		
**Technological advances supporting remote working**<br>• Maintenance of networks<br>• Inclusive approach to working	**Loss of resource**<br>• Loss of outputs<br>• Fiscal barriers<br>• Loss of research team & associated HR issues<br>• Decrease in academic capacity due to clinical workload<br>• Lack of research supervision<br>• Practical barriers (e.g. data storage)<br>• Absence of pastoral		

For peer review only

	support	

For peer review only

Theme 1: Opportunities and enablers

The pandemic presented many opportunities and enablers. These spanned academic and clinical work as well as participants' personal lives. Subthemes broadly fell into three categories; 1) new opportunities for research, 2) support, 3) maintenance of normality and 4) technological advances supporting remote working.

New emerging opportunities

Participants recognised the immediate opportunity to explore new avenues of research related to the pandemic.

> "*So I've been asked to, and volunteered to, be part of the Research Ethics Committee Coronavirus Response which means that every week now I've had twenty-four hour turnaround for a coronavirus study of some sort or another … I have been involved with developing two national and one international coronavirus studies over this time…an enormously exciting but terrifying roll out of research…*" [Male Medic - diary]

> "*…there might be a positive if you were, you know, an Oxford University clinician looking at vaccine so you'd obviously you get a lot of funding and, you know, good opportunity to, to research Covid based things… I suppose if you've never done a clinical trial before and you were a clinical academic, getting involved in some of the trials that they're doing at the moment, like the Recovery Trial, that could be a positive for you if you're in that particular situation…" [*Female Medic – interview*]*

Associated with this was the need for rapid research dissemination, providing clinical academics with a chance to develop their portfolio. This resulted in the forging of new research collaborations supported by the quick turnaround of ethical approval in order to roll out impactful research. More senior clinical academics reported opportunities for career development such as being invited to serve on Research Ethics Committees. In addition to the new research territory, COVID-19 presented a new lens through which participants were able to think about their existing research.

For some, salient amongst the opportunities was the free time which was created during the pandemic, particularly due to the absence of social commitments and commuting. Some clinical academics found this spare time to be an opportunity to focus on academic research activities and the production of impactful research. Others felt this was as an opportunity to focus on clinical work, as well as family and household commitments.

> "*I guess it will give me a chance to focus on, you know, having a bit more time at home with family and, and just doing clinical work, which I think, you know, is what I wanted to focus on for the moment.*" [Female Medic – Audio Diary]

Support

Participants presented examples of the positive impact of support networks locally, nationally and internationally. The abrupt emergence of COVID and subsequent lockdown meant that many clinical academics halted their academic work and returned to clinical practice. This created an element of uncertainty, and in some cases anxiety about completing their academic work. However, a widely circulated statement

For peer review only

from the UK's main funding bodies reassuring clinical academics that they would support the extension of research post-COVID was well received.

> "*The [funder] have said that they will support extensions if they're needed so I do have that to kind of fall back on.*" [Female Medic – Audio Diary]

An immense amount of support was provided by academic organizations at the beginning of the pandemic. Support from supervisors, departments and funding bodies was identified as enabling and empowering for participants. For example, the provision of childcare nurseries, enabling clinical academics to return to full-time clinical practice when required.

Maintenance of normality

'Maintaining the status quo' during the pandemic was an enabler for many. From the beginning of the pandemic many professions have seen large proportions of redundancy. However, this has not been the case for many of the clinical academics that participated in this study. Clinical academics who have had the flexibility of undertaking either clinical or academic work during the pandemic, were grateful and felt generally lucky to have a job during COVID and one they could go back to post-COVID. Opportunities that strengthened the resolve of participants included the option to flex hours between roles. Specifically, it was also noted that going back to clinical practice full-time was perceived as advantageous due to more structure compared to academic work. Additionally, the retention of social interaction, structure and workload was seen as an opportunity.

> "*In these times of COVID-19 some people have kind of lost their structure and workload, so I feel grateful that hasn't happened to me.*" [Male Medic – Audio Diary]

Technological advances supporting remote working

Participants advocated the use of video-conferencing platforms, citing them as enablers to maintain networks and collaborations during the pandemic as well as the inclusivity afforded by digital spaces.

> *"…my colleagues have become much more able to work online using Microsoft Teams and there have been more meetings recorded and more webinars which meant that as a part-time person I've been able to attend some of the things that previously I used to enquire and they used to say that this is not recorded and it is not being broadcast, so that has been excellent." [Female Medic – Interview]*

<u>Theme 2: Barriers</u>

There were multifaceted barriers related to the direct consequences of the immediate impact, reactive and mid-term barriers, and longer-term implications. Three subthemes were identified; (1) lockdown, (2) demand of teaching responsibilities and, (3) loss of resource.

Lockdown

Most of the barriers described by participants related to the negative consequences of lockdown. A barrier evidenced by particularly emotive data was that of working at home. This entailed the need to balance the multiple commitments related to childcare, housekeeping, and productivity. There were far reaching consequences of the ways in which working from home impacted on participants' roles. Childcare was not seen as compatible with a clinical academic role. Both clinical (e.g. telephone clinics and clinical administration) and academic work was often taking place at home, leading to feelings of isolation.

> "*I've spent a lot of time doing very intensive blocks of clinical work and very intensive blocks of research, research policy into practice work…I realised it's a Thursday today, I realised I had worked for three weeks solid and I'd not had a full day off in that time and most of those days were ten or twelve hour days…and I have now worked a further eleven days, with the last four days being fourteen hour days, without a break*". [Male Medic- Interview]

Mental and physical health outcomes were described as a consequence of COVID, particularly due to the increased workload.

> "*Generally, the COVID experience has been exhausting on many levels; emotionally from the worry about the number of people who will die, worry about being redeployed, worry for my children's wellbeing.*" [Male Medic- Interview]

> *"My anxiety is elevated due to being Asian, will I get sick and lose time for research?"* [Female Medic-Interview]

Concerns arose over how to effectively manage competing time demands. Some also spoke about a loss of 'downtime' and feeling 'stuck in limbo'. The shift in work-time meant that participants had less time for recreational activities which impacted on wellbeing.

Both males and females were impacted by the move to home working and balancing childcare with full-time work. The time pressures were apparent in many situations where participants struggled to navigate competing demands.

> "*… the Pro Vice Chancellor had sent an email saying how exciting it was that there's a female President at the Medical School, a female Dean, a female Pro Vice Chancellor of Medicine and a female Vice Chancellor at University, and that made me reflect what would happen if I sent a similar email delighting the fact that there's males in all of those roles. I imagine there'd be some disdain… they're powerful and take revenge…I don't think that's appropriate.*" [Male Medic – Audio Diary]

There were also concerns over positive discrimination in how gender roles were recognised, with a particular focus on senior positions. Such comments alluded to notions of male fragility and toxic masculinity, although the intent cannot be stated in absolute terms.

> *"…I do sometimes think that even, males might be at a, particularly at the end of, top end of their career, might be at more, slight disadvantage because, particularly with Athena SWAN there's obviously an emphasis, and maybe rightly so, in supporting women, you know, particularly take senior posts like chairs and therefore I do think sometimes it's almost got the point where it's the first discrimination where men might be overlooked, you know, because there's a keenness to promote women, and I, in a sense I understand the rationale for that, and it might get worse with the pandemic."* [Male medic- interview].

> *"it's kind of bordering on positive discrimination around research professorships, I think men are needed…"* [Male medic- interview].

Demands from teaching responsibilities

The analysis identified the negative impact of having to rapidly produce teaching and assessment materials for online delivery, frequently coupled with a lack of support and appreciation, as well as the time consumed. In many cases, this was in addition to clinical responsibilities.

> "*As clinical academics we are scavenging time in between the clinical parts to crack on and do our academic work*." [Male Medic – Audio Diary]

There was worry over a future lack of recognition within portfolios for teaching activities undertaken, and a frustration over negotiating the various new technologies required to deliver teaching.

Loss of resource

The impact of COVID has been significant for participants and extends from fiscal and time to human resource issues. There were concerns over the loss of time in relation to academic outputs and not fulfilling previously arranged activities. A lack of overall supervision was noted, including much needed pastoral support.

> "*There is an absence of pastoral support. Supervision is different, it's about skills and development not always about just how you feel. Sometimes you need a shoulder to cry on*". [Female Medic – Audio Diary]

Given the measures in the academic field where outputs are often used as a productivity marker, the participants were unsure how they could compensate loss of time and the ways in which their activities could be demonstrated and valued.

Tangible barriers were highlighted in the monetary implications for how loss of time could be compensated. Participants made calls for funders to extend time and funding for projects that otherwise may have been completed on time.

> "*It's bloody terrifying when it comes to applying for new stuff though because the deadlines haven't really moved much and there's no head space and there's no time to get the stuff really done properly and that's really scary but we've got to get the clinical work done first. It's the only way*." [Male Medic – Audio Diary]

Many universities initiated a recruitment freeze which meant in some cases that methodological expertise was lost. As clinical academics are often on a set educational employment path for a discrete time period this created disruption to long-term career plans.

Theme 3: Personal characteristics and social identity

A key theme revealed was the impact of COVID-19 on participants' personal characteristics and social identity. This theme specifically considers how individuals perceived themselves as being clinical academics and the issues associated with the label 'clinical academic'.

Ambivalence and identity of a clinical academic

Some participants were conflicted about their identity as clinical academics and often experienced 'imposter syndrome'. Imposter syndrome, as described by the participants, refers to a feeling that they were not adequately qualified to be called clinical academics. Others expressed pride in being classed as clinical academics, however, they expressed that their identity as clinical academics was misunderstood by clinical and academic colleagues. Participants described problems experienced with negotiating both clinical and academic identities during the pandemic.

> "*Trying to tussle where you fit between university and clinical world at the moment is, is also hard*." [Male Medic– Audio Diary]

Participants highlighted that in their working life they do not sit neatly in either category, which makes it difficult to identify as either an academic and/or a clinician. This feeling has been intensified by the pandemic. Most professionals have an educational background where they have developed peer support and networks allowing for guidance, but for clinical academics, their varied pathways means they often lack this important support network.

Protected characteristics

Protected characteristics of clinical academics, particularly the BAME community who have been more widely affected by COVID-19, was a key subtheme identified. Increased anxiety was highlighted and participants who were from BAME backgrounds expressed fear about returning to clinical work. Muslim clinical academics also expressed the difficulty they faced due to Ramadan taking place during the already difficult time. The focus was largely on physical wellbeing due to heavy workload alongside being unable to eat or drink.

Gendered differences were evident throughout this study, with female participants specifically expressing the struggles of maternal identity, highlighting problems faced with childcare responsibilities as well as having to share these with their partners alongside work commitments.

> *"I'm not formally planning to carry on with academia and that's a decision that's kind of come about because of, you know, childcare responsibilities and I just*

For peer review only

> *felt that I was going to be stretched too thin if I was trying to be a mum and be a GP and be an academic as well.*" [Female Medic– Audio Diary]

Female participants felt that due to gender stereotypes from partners and work colleagues they were having to take a leading responsibility in childcare during this time.

> "*I think that COVID has been a disaster for feminism...the disagreements that I've had with [husband] have been over feeling like a 1950s housewife suddenly then to suddenly home schooling children and trying to stay sane, stay safe, keep everybody okay, whereas he still is doing exactly what he does, work...*" [Female Medic– Audio Diary]

Whilst participants noted intersectionality, their narratives were mostly differentiating their experiences due to gender. Women explicitly stated that they became less tolerant of the gender issues raised as time in lockdown progressed.

Women reported the emotional turmoil associated with being primary caregivers.

> *"Yet another day of working from home while helping with young kids who are upset at the disruption to their lives. The most difficult thing is trying to calm my children's' worry over COVID."* [Female Dentist– Audio Diary]

Increased perceived importance of clinical academics

Despite the tensions that have been caused by the pandemic, some noted that the work and identity of clinical academics have actually been strengthened. COVID has required rapid research to be undertaken and clinical academics are perfectly suited to this opportunity. The fact that qualified clinical academics have a foot in both research and clinical practice has facilitated a renewed appreciation of their identity during this pandemic.

<u>**Theme 4: Future fears and uncertainty**</u>

This theme related to numerous unanswered questions of participants including their fears and anxieties. Returning to the clinical environment was one major cause of anxiety, as well as the future of their research and funding.

Returning to the frontline

Discourse regarding a full return to clinical practice utilised many analogies to warfare. One source of internal conflict for participants was their inability to respond positively to the 'call to arms', particularly where they felt they had deskilled in some areas due to their focus on academia in recent years. Participants described requests to abandon research and assume clinical roles. Added to this anxiety was an inability to manage workload and family life.

> "*Well all of the academic trainees have been pulled on to the full-time clinical work. As clinical academics we are scavenging time in between the clinical parts to crack on and do our academic work and certainly emotionally I'm finding it enormous strain, mainly because the clinical work itself has not so much increased in the intensity but the massive decrease in staff numbers, partly because of illness but a lot because of isolation and shielding*

For peer review only

requirements that have gone on, has hugely increased the amount of work that all of us that are at work are doing." [Male Medic– Audio Diary]

Additionally, the fear of contracting COVID-19 was expressed, with the majority of participants having to return to full clinical practice. Clinical academics who expressed fear of contracting COVID-19 were those with predisposing factors and BAME backgrounds. Additionally, knowing someone who had died on the frontlines increased clinical academics' perception of their own risk.

Misconceptions and concerns about the future

From the onset of the pandemic, participants have reported concerns over their progression and future careers. Participants also aired concerns about the lockdown restrictions being lifted prematurely, stating that this could have further negative implications on their clinical and academic work. Uncertainty around COVID-19 has caused misconceptions, concerns and scaremongering, ranging from the prospect of more lockdowns to the potential for a return to fulltime clinical work only. There has also been an absence of concrete information for clinical academics. Amongst participants, there is a general fear and uncertainty about the fiscal impact on research funding and the future clinical academia.

"*Unfortunately my grant holders haven't sort of committed themselves to being able to extend my funding due to various reasons, so yeah, so there's uncertainty with that as well.*" [Female Medic– Audio Diary]

Issues such as pay cuts and a loss of staffing resources within academic departments caused further anxieties.

"The news on pay cuts is a bitter pill to swallow when working in a department where teaching commitments have increased, I have to say. Clinicians may decide to switch to clinical only in some cases." [Male Medic– Audio Diary]

These anxieties have been further intensified following a lack of professional and social networking opportunities, especially as the pandemic intensified and lockdowns progressed.

Box 1: Case examples (CE) of the initial impact of COVID-19 on participants with intersecting identities (pseudonyms utilised)

Case study 1: Asian, Mother, Muslim, Medical Registrar/Doctoral Fellow
Zainab, a Medical Registrar in Neurology and Doctoral Fellow is a married, Asian, Muslim, mother of three young children. She is 'proudly intersectional'. Zainab works full time and spends about 90% of her time on her academic work. She has recently put her details down to be called to the frontlines and is currently on the standby list. As an Asian doctor, living and working in a city, she feels particularly vulnerable and has fears about her risks of contracting COVID. Her anxieties regarding the virus increased over time as research linked BAME ethnicity to outcomes. She knows of a doctor who died on the front line, they were a relative of one of her closest friends. Although she realizes that susceptibility to the disease is multifactorial, she can see that people from minority ethnic

For peer review only

groups are disproportionately affected. According to her, "*it's not known why [there's a disproportionately higher number] I think it is multifactorial whether there's underlying kind of health vulnerabilities that are more prevalent in certain ethnic groups that make them more vulnerable or whether there's more complex issues to that*." Despite the negative implications that the COVID pandemic and lockdown have brought, she finds a great deal of positives from the supportive academic institution.

Case study 2: White, Mother, Clinical Lecturer

Rose, a Medical Registrar and Academic Clinical Lecturer, has been working at home where she is married to a clinical professor with two children. Rose works part-time. She is struggling with the balance of family and work commitments, stating that lockdown has reinforced sexist gender roles. Rose feels that her research has taken a backseat while her husband's work has been prioritised, "*I genuinely think he thinks his work is more important, I genuinely think that's what he believes*." She reports a decline in mental health due to the pressures of lockdown and family dynamics, "*I think that COVID has been a disaster for feminism...the disagreements that I've had with [husband] have been over feeling like a 1950s housewife suddenly, then to suddenly have to provide home schooling children and trying to stay sane, stay safe, keep everybody okay, whereas he still is doing exactly what he does [work]...so I think that, that, that's my big worry is that actually COVID's been a step back*." Rose's anxiety about her research profile increased the longer lockdown restrictions were in place.

For peer review only

Discussion

This study presents a unique, qualitative data-driven reflection of the national clinical academic landscape during nine months of the COVID-19 pandemic. The in-depth interview data are supported by real time audio diaries. This multi-faceted approach has facilitated access to “periods of change and flux” particularly during the pandemic, and allowed the research team to capture the impact of COVID-19 in real-time which facilitated accurate data capture [24, 25]. The audio diaries are also advantageous owing to their ease of completion, which typically results in lower levels of attrition. [25] Participants were also permitted to submit written entries, but this was not deemed to be a limitation, especially as discourse analysis was not planned. Permitting flexibility also prevents diary entries from becoming formulaic [25]. Whilst the authors recognize that there is often a self-selection bias associated with studies of this nature, participants were felt to be balanced in their presentation of barriers and enablers [26]. Audio diaries varied in length and thus depth, however, there was no variability in the richness of data. This study was funded and thus the qualitative nature stipulated – later we discuss ideas for future research utilising quantitative and survey approaches. Such work could add to our findings.

Clinical academics were impacted by COVID-19 in multiple ways leading to a wide range of barriers, challenges and opportunities underpinned by their personal and professional identities. The selflessness and dedication of the study participants to contribute to the clinical response to the COVID-19 pandemic is evident in the data. Clinical academics returning to the NHS during the pandemic, had added value with respect to their research experience[27].

Our findings identified the numerous perceived barriers to continuing academic activity within the family, academic and clinical contexts. What is clear is that pre-existing barriers to academic activity have become magnified during the COVID-19 pandemic. Although such barriers are not insurmountable, they have proved and continue to prove stressful for the participants. The impact on their future career plans is already evident. The restrictions on face-to-face contact, international travel, uncertainties over clinical and academic training and funding extensions, home working, and, in many cases, redeployment to frontline clinical duties have all impacted on academic activities. Tension was evident when academic trainees who switched to full time clinical work and sacrificed their academic time described how they felt disadvantaged in comparison to trainees who have been able to maintain research.

Female participants described barriers that related to their gender, as well as for many, their maternal status, with such biases having been previously well documented in the literature [18, 20, 28-30]. Our findings in the themes (2 and 3) and case studies, suggest that the pandemic and resultant lockdown has, in many cases, exacerbated the discrimination faced by many women in clinical medical and dental academia. A survey of dental clinical academics in 2018, found that overall female representation in the academic grades in Dentistry is increasing - mirroring gender representation in dentistry as a whole [31]. Given our data, it is possible that the COVID-19 pandemic could have an even more significant negative impact on female Dental Clinical Academics going forward.

Theme three also identified a number of females in our sample who described feeling and being compelled to assume, to a greater degree than before the pandemic, gender stereotypical roles in the home. Specifically, when faced with competing responsibilities females appeared to feel under more pressure than their male partners to allocate more time to childcare and household tasks. Indeed, there was a recurrent motif of narratives relating to the '1950s housewife' stereotype within the family context [32]. In this regard, our findings are consistent with media accounts that the lockdown has placed a disproportionate burden on females, who frequently report taking on the bulk of the childcare, domestic duties and home-based education of school-aged children [33, 34]. It has been postulated that many females are currently doing 'second and third shifts' with regard to housework and welfare after their first shift of childcare or paid work [32]. Prior to COVID-19, there was evidence that parental working determined that, on average, females spent two to three times as much time on care and housework compared to men [35]. Our findings suggest that these effects have become more pronounced as a result of the pandemic and are impacting significantly on female clinical academics. Our data may go some way towards explaining reported declines in publications by female academics [36].

The findings raise an alarm that prolonged periods of lockdown could reverse general trends towards a more equitable distribution of household labour [35]. More concerningly, it may substantially put back efforts to achieve a more gender representative body of clinical academics, characterised by gross under representation of female, explored by a recent systematic review [9]. This has been shown to not merely be a matter of social equality; the male dominance of science in general has served women's health poorly [37].

Of note within our data was the indication from male participants that they were experiencing positive discrimination. We would not wish to overinterpret; however, our analysis of the participants' narratives alluded to some potential degree of toxic masculinity [38] and fragility. Women's continued fight for equality within the workplace can often lead to discomfort from those who have typically held most privilege within the discussion. Although the discomfort articulated from males in our sample was a minority, it was expressed with conviction, especially in terms of positive discrimination.

There are a number of steps that could be taken to mitigate the impact of the COVID-19 pandemic on clinical academia as a whole and the risk of reduced long-term clinical research capacity, especially the loss of female academics. Funders and employers could intentionally target support at those with caring responsibilities during lockdown and the following recovery period. As the pressing clinical need recedes then prioritising the return of female academics to their research activities may also offset the bias more generally experienced by female scientists both before and during the pandemic. Whilst virtual networks of collaborators have organically sprung up in places during the lockdown, more of these could be strategically formed to counter the isolation felt by many academics. This support should also extend to academics who are primarily clinical educators, and have historically felt marginalised and undervalued [39]. Indeed, some of the participants in the present study voiced perceptions of a lack of support and appreciation from both their

For peer review only - http://bmjopen.bmj.com/site/about/guidelines.xhtml

academic and clinical employers, and colleagues. Both existing and trainee clinical academics reported a perception that neither non-academic clinical colleagues or non-clinical academics fully appreciated the competencies and the time demands of their academic roles. This perception has been reinforced by the COVID-19 pandemic. Going forward, enhanced collaboration and empathy between University and Clinical staff and employers could be targeted to remedy this.

As the UK navigates its immediate response to COVID-19, it is important to consider the potential implications for those whose careers are most vulnerable and require additional nurturing. There needs to be increased flexibility in career pathways and expectations, along with a review of funding and support offered. Participants requested flexibility with existing funding to make changes to study protocols and costings to enable them to pursue opportunistic COVID related research. Leaders in academic institutions and funding bodies must be cognisant of the varying degrees to which clinical academics will have been able to mitigate for COVID-19 in their assessments of applications for funding and positions and must revise their expectations for dissemination and impact. Causal explanations of the issues faced during COVID-19 will be multifaceted and sensitive to personal context. Teaching commitments must be valued and accounted for, as well as physical and mental health. It has been shown that clinical academics create national wealth as well as health [40]. We are heading into an inevitable global financial recession, if not depression, likely to also be characterised by an increase in mental and physical health needs which will undoubtedly impact on the clinical academic workforce and funding.

The infrastructure for medical and dental clinical academia is well established, yet our data suggest that better support is required to develop future clinical academic roles and enhance the provision of evidence-based patient care in the post-COVID-19 era. Participants report substantiated concerns over access to funding and positions once the status quo is resumed.

Whatever interventions and strategies are implemented, heed needs to be taken to the findings of Laver et al., who reported that interventions within clinical academia that require those they are designed to support, so called ‘bottom-up’ approaches, to bear the brunt of the workload are less likely to succeed [41]. Their review advocates the use of ‘top-down’ approaches, led by change in practice at a management level. Finally, their stance was that ‘something is better than nothing’.

Our findings offer further insight into the debate surrounding access to, and maintenance of, clinical academic careers [42]. We add weight to the evidence surrounding gender disparity. The well documented decline in the clinical academic workforce, the subject of contemporary research, [9, 43] could continue if strategies to support clinical academics are not implemented in the aftermath of the COVID-19 pandemic. Ongoing commitment to supporting and developing clinical academics is required, acknowledging the implicit biases facing females and parents in reaching their potential, and those with other competing demands on their time. Of paramount importance is the need for greater recognition of the value and contribution made by clinical academics to both the clinical and academic workforces to which they contribute. The reports from a recent national study by Finn and colleagues presented a number of proposed interventions to support clinical academics in the

immediate wake of the pandemic, in addition to recommendations for ensuring the long-term sustainability of the clinical academic pipeline. Headlines from qualitative data included the need for mentorship, flexibility and transparent processes, as well as recognition of the differential experiences of marginalised groups[10].

Our findings also emphasise the need for guidance for aspiring clinical academics. As one remarked, “I just need guidance on next steps. It is just so challenging to think about who might help me.” Furthermore, without urgent action COVID-19 has the potential to increase the gender divide seen within clinical academia, and as demonstrated within recent studies [40-42]. It is hoped that the narratives in this study initiate a dialog between clinical academics, funders and institutions to successfully navigate the clinical academic pathways moving forward. Support and mentoring may be key to ensuring the clinical academic pipeline.

Future research

Future research could include collecting quantitative data to track the career progression, publication rates and grant successes for clinical academics to look for patterns, trends and potential biases based upon their protected characteristics and availability of support or mentoring. Survey data could supplement the findings of this study.

** ** **

Contributors
GF, PC, PT, MK, JM and JB conceived the study. GF, PC, PT, JB and JM obtained funding. ET, GF, JB, PC, ABK and AK collected data and conducted the analyses. GF, AK, JB, PT, CS and PC recruited participants and drafted the manuscript. ABK and AK curated the data. All authors reviewed the final manuscript.

Competing interests

The authors declare no competing interests.

Declaration
The manuscript is an honest, accurate, and transparent account of the study being reported. There are no omissions or inaccuracies. Participants have engaged in member checking of results and are briefed of findings.

Funding
This publication presents independent research funded by Cancer Research UK (Award Number: C71037/A29824), The Academy of Medical Sciences, Health Education England, The Medical Research Council, The National Institute for Health Research (NIHR) and The Wellcome Trust. The views expressed are those of the author(s) and not necessarily those of The Academy of Medical Sciences, Cancer Research UK, Health Education England, The Medical Research Council, The

review only

National Institute for Health Research (NIHR), The Wellcome Trust or the Department of Health and Social Care.

Acknowledgments
The authors wish to thank all the clinical academics who participated in this study. The authors acknowledge the work of the wider research team involved in this large-scale research project and thank them for their contributions. The authors also appreciate the insight of the project steering group at the Clinical Academic Training Forum. Thanks go to 'Women in Academic Medicine', and the British Medical Association for assistance in recruiting participants.

Data availability statement
Deidentified transcripts would be made securely available to other researchers if a written case for access is made to the research team and permission is granted by the funders of the study. This should be sent directly to the corresponding author GF.

For peer review only

References

1. Brenner, A.M., *Supervising the tearful trainee: opportunities for professional growth and learning.* Academic psychiatry : the journal of the American Association of Directors of Psychiatric Residency Training and the Association for Academic Psychiatry, 2014. **38**(4): p. 497-501.
2. Hourston, G.J., *The impact of despecialisation and redeployment on surgical training in the midst of the COVID-19 pandemic.* International Journal of Surgery (London, England), 2020. **78**: p. 1.
3. Clinical Academic Training Forum, *Clinical Academic Training Forum: Subgroup to support returning clinical academics from the COVID-19 front line*. 2020.
4. Guarino, C.M. and V.M. Borden, *Faculty service loads and gender: Are women taking care of the academic family?* Research in Higher Education, 2017. **58**(6): p. 672-694.
5. Ranieri, V., et al., *Factors that influence career progression among postdoctoral clinical academics: a scoping review of the literature.* BMJ Open, 2016. **6**(10): p. e013523.
6. Flaherty, C. *No Room of One's Own: Early journal submission data suggest COVID-19 is tanking women's research productivity*. 2020 [cited 2020 21st April 2020]; Available from: https://www.insidehighered.com/news/2020/04/21/early-journal-submission-data-suggest-covid-19-tanking-womens-research-productivity.
7. Pinho-Gomes, A.-C., et al., *Where are the women? Gender inequalities in COVID-19 research authorship.* BMJ Global Health, 2020. **5**(7): p. e002922.
8. McEwan, J., *Engaging women in academic medicine in the UK: report of a workshop at the Association of Physicians Annual Meeting, 2 April 2009.* QJM: An International Journal of Medicine, 2010. **103**(9): p. 635-639.
9. Brown, J.V., et al., *From the sticky floor to the glass ceiling and everything in between: protocol for a systematic review of barriers and facilitators to clinical academic careers and interventions to address these, with a focus on gender inequality.* Systematic reviews, 2020. **9**(1): p. 26.
10. Finn GM, B.J., Evans C, Raine G, Stewart L, Uphoff E, Kehoe A, Buchanan J, Balogun-Katung A, Tiffin P, Taylor E, Crampton P, Morgan J., *From the sticky floor*

to the glass ceiling and everything in between: A systematic review and qualitative study focusing on gender inequalities in Clinical Academic careers. Final report. . 2020.

11. Finn GM, B.J., Evans C, Raine G, Stewart L, Uphoff E, Kehoe A, Buchanan J, Balogun-Katung A, Tiffin P, Taylor E, Crampton P, Morgan J., *Inequalities in UK clinical academic careers: a systematic review and qualitative study. Short report.* . 2020.
12. Gordon, L., et al., *Multiple and multidimensional transitions from trainee to trained doctor: a qualitative longitudinal study in the UK.* BMJ open, 2017. **7**(11).
13. Braun, U.K., et al., *The utility of reflective writing after a palliative care experience: Can we assess medical students' professionalism?* Journal of Palliative Medicine, 2013. **16**(11): p. 1342-1349.
14. Braun, V. and V. Clarke, *Using thematic analysis in psychology.* Qualitative research in psychology, 2006. **3**(2): p. 77-101.
15. Kiger, M.E. and L. Varpio, *Thematic analysis of qualitative data: AMEE Guide No. 131.* Medical Teacher, 2020: p. 1-9.
16. Roberts, K., A. Dowell, and J.-B. Nie, *Attempting rigour and replicability in thematic analysis of qualitative research data; a case study of codebook development.* BMC medical research methodology, 2019. **19**(1): p. 66.
17. Braun, V. and V. Clarke, *Successful qualitative research: A practical guide for beginners*. 2013: sage.
18. Williams, J.C. and N. Segal, *Beyond the maternal wall: Relief for family caregivers who are discriminated against on the job.* Harv. Women's LJ, 2003. **26**: p. 77.
19. Brown, M.E., et al., *'Too male, too pale, too stale': a qualitative exploration of student experiences of gender bias within medical education.* BMJ open, 2020. **10**(8): p. e039092.
20. Williams, J.C., *Hitting the maternal wall.* Academe, 2004. **90**(6): p. 16.
21. Braun, V. and V. Clarke, *To saturate or not to saturate? Questioning data saturation as a useful concept for thematic analysis and sample-size rationales.* Qualitative research in sport, exercise and health, 2021. **13**(2): p. 201-216.
22. Varpio, L., et al., *Shedding the cobra effect: problematising thematic emergence, triangulation, saturation and member checking.* Medical education, 2017. **51**(1): p. 40-50.
23. Malterud, K., V.D. Siersma, and A.D. Guassora, *Sample size in qualitative interview studies: guided by information power.* Qualitative health research, 2016. **26**(13): p. 1753-1760.
24. Monrouxe, L.V., *Negotiating professional identities: dominant and contesting narratives in medical students' longitudinal audio diaries.* Current Narratives, 2009. **1**(1): p. 41-59.
25. Crozier, S.E. and C.M. Cassell, *Methodological considerations in the use of audio diaries in work psychology: Adding to the qualitative toolkit.* Journal of occupational and organizational psychology, 2016. **89**(2): p. 396-419.
26. Collier, D. and J. Mahoney, *Insights and pitfalls: Selection bias in qualitative research.* World Politics, 1996. **49**(1): p. 56-91.
27. Rimmer, A., *Covid-19: How clinical academic trainees came back to the wards en masse.* bmj, 2021. **373**.
28. Crosby, F.J., J.C. Williams, and M. Biernat, *The maternal wall.* Journal of Social Issues, 2004. **60**(4): p. 675-682.
29. Williams, J.C., *The glass ceiling and the maternal wall in academia.* New Directions for Higher Education, 2005. **2005**(130): p. 91-105.

For peer review only

30. Halley, M.C., et al., *Physician mothers' experience of workplace discrimination: a qualitative analysis.* BMJ, 2018. **363**: p. k4926.
31. Council, D.S., *Professorial roles for dentists fall 8.3% since 2015.* British Dental Journal, 2018. **225**(5).
32. Chung, H. *Return of the 1950s housewife? How to stop coronavirus lockdown reinforcing sexist gender roles*. 2020; 21st April 2020]. Available from: https://theconversation.com/return-of-the-1950s-housewife-how-to-stop-coronavirus-lockdown-reinforcing-sexist-gender-roles-134851.
33. Ferguson, D., *'I feel like a 1950s housewife': how lockdown has exposed the gender divide*, in *The Guardian*. 2020.
34. Alon, T.M., et al., *The impact of COVID-19 on gender equality*. 2020, National Bureau of Economic Research.
35. Wishart, R., et al., *Changing patterns in parental time use in the UK*. 2019.
36. Viglione, G., *Are women publishing less during the pandemic? Here's waht the data say*, in *Nature*. 2020. p. 365-366.
37. Burford, B., et al., *Professionalism education should reflect reality: findings from three health professions.* Medical Education, 2014. **48**(4): p. 361-374.
38. Harrington, C., *What is "toxic masculinity" and why does it matter?* Men and Masculinities, 2021. **24**(2): p. 345-352.
39. Kumar, K., C. Roberts, and J. Thistlethwaite, *Entering and navigating academic medicine: academic clinician-educators' experiences.* Medical education, 2011. **45**(5): p. 497-503.
40. Innovation, B. and G. Team, *Bioscience 2015: Improving National Health.* Increasing National Wealth, 2003.
41. Laver, K.E., et al., *A systematic review of interventions to support the careers of women in academic medicine and other disciplines.* BMJ open, 2018. **8**(3).
42. Rothwell, P.M., *Medical academia is failing patients and clinicians*. 2006, British Medical Journal Publishing Group.
43. Fitzpatrick, S., *A survey of staffing levels of medical clinical academics in UK medical schools as at 31 July 2011.* London: Medical Schools Council, 2012.

For peer review only

Appendix 1: Interview and example audio diary prompts for participants during COVID-19

Example interview stems for participants during COVID-19
Semi-structured approach thus will be guided by participants.<br>1. **Introductions**<br><br>2. **Please describe your role and career path to date -** *both clinical and academic*<br><br>3. **What are your experiences of clinical academia?**<br><br>4. **What factors impacted upon your decision to pursue (or not pursue) a career in clinical academia?**<br>• *What factors make clinical academia an attractive career?*<br><br>5. **What has your experience with awards/ funding been like? (if any)**<br>• *Application process? (facilitators/barriers)*<br>• *Support through process*<br>• *Why did/didn't continue? (e.g. after PhD why didn't do lectureship?)*<br>• *If not applied, why not?*<br>• *Did this experience influence your choice in career pathway?*<br><br>6. **What barriers and enablers have you faced within clinical academia?**<br>• *What made you continue/not continue?*<br>• What stops people returning to clinical academia?<br>• How do you negotiate the 2 career paths?<br>• What goes wrong in institutions that funders can help with?<br>• What worked well/ less well with other funders you have experienced?<br>• Impact of the COVID-19 pandemic<br><br>7. **What could be done to improve equal representation within clinical academia?**<br><br>8. **Describe any interventions to promote access to clinical academia or reduce discrimination that you have experienced.**<br>• *What worked?*<br>• *Given your context, what would you recommend?*<br><br>9. **What existing or new interventions could help to reduce attrition in clinical academic careers?**<br><br>10. **How can organisations support trainees and clinical academics in their career decisions and academic pathways?**
**Example audio diary prompts for participants during COVID-19**
Participants were able to reflect on all prompts any situation pertaining to Clinical Academia that they wished. Prompts were broad and participants were informed of the interest in protected characteristics.

How has COVID-19 impacted upon your work as a clinical academic?	What have you heard about funding (opportunities, threats, remit etc) post COVID-19?	Has COVID-19 had a detrimental impact on or changed your career plans?
How has the expectation of the impact of COVID-19 differed from the reality?	How have your protected characteristics impacted upon your experience of clinical academia during COVID-19?	BAME participants: Has the emerging data on the enhanced risk to the BAME workforce from COVID-19 had any impact on you?
Has COVID-19 had a detrimental impact on or changed your research?	What barriers and enablers have you faced during COVID-19?	Parents/ carers: How have your responsibilities impacted upon your ability to work?

For peer review only

Reference:

Tong A, Sainsbury P, Craig J. Consolidated criteria for reporting qualitative research (COREQ): a 32-item checklist for interviews and focus groups. International Journal for Quality in Health Care. 2007. Volume 19, Number 6: pp. 349 – 357

Consolidated criteria for reporting qualitative studies (COREQ): 32-item checklist

No	Item	Guide questions/description
		**Domain 1: Research team and reflexivity**
Personal Characteristics		
1.	Interviewer/facilitator	Which author/s conducted the interview or focus group?<br>GF, MK, PC, JAG, AB<br>Page 5
2.	Credentials	What were the researcher's credentials? *E.g. PhD, MD*<br>The researchers had a range of qualifications including PhD, MD, MBChB, MMEd, PGDipMEd, MRCpsych, FRCP, FAcadMEd, FRCPsych, and MSc.<br>Page 5
3.	Occupation	What was their occupation at the time of the study?<br>GF was Professor of Medical Education, PC was a lecturer, MK and AB were Research Fellows, ET was a medical student. PAT, JM, CS and JAG were clinical academics.
4.	Gender	Was the researcher male or female?<br>Three were male, 6 were female<br>Page 5
5.	Experience and training	What experience or training did the researcher have?<br>All trained in research methods and well published in the field.<br>Page 5
Relationship with participants		

For peer review only - http://bmjopen.bmj.com/site/about/guidelines.xhtml

No	Item	Guide questions/description
6.	Relationship established	Was a relationship established prior to study commencement?<br><br>No<br>Page 4
7.	Participant knowledge of the interviewer	What did the participants know about the researcher? e.*g. personal goals, reasons for doing the research*<br><br>Participants were provided with an information sheet outlining the reasons for doing the research. They had the opportunity to ask questions and were able to access a study website and twitter account for information.<br><br>Page 4/5
8.	Interviewer characteristics	What characteristics were reported about the interviewer/facilitator? e.g. *Bias, assumptions, reasons and interests in the research topic*<br><br>A reflexivity exercise was conducted to demonstrate the researchers' relationship to the study findings. This is provided on the project website.<br><br>Page 5
		**Domain 2: study design**
Theoretical framework		
9.	Methodological orientation and Theory	What methodological orientation was stated to underpin the study? *e.g. grounded theory, discourse analysis, ethnography, phenomenology, content analysis*<br><br>As stated on page 5of the methods section, the methodological orientation underpinning this study is reflexive thematic analysis
Participant selection		

For peer review only - http://bmjopen.bmj.com/site/about/guidelines.xhtml

No	Item	Guide questions/description
10.	Sampling	How were participants selected? *e.g. purposive, convenience, consecutive, snowball*<br>As stated on page 4, participants were selected using purposive sampling with snowballing<br>Page 4
11.	Method of approach	How were participants approached? e.*g. face-to-face, telephone, mail, email*<br>As stated on page 4, participants were recruited through social media, email listserv, referrals
12.	Sample size	How many participants were in the study?<br>As stated on page 6, there were 104 participants in the main dataset
13.	Non-participation	How many people refused to participate or dropped out? Reasons?<br><br>As stated on page 6 of the methods section, no (0) people refused to participate or dropped out from our study
Setting		
14.	Setting of data collection	Where was the data collected? e.*g. home, clinic, workplace*<br><br>As stated in the methods section on pages 4-5, data were collected using telephone interviews and audio diaries
15.	Presence of non-participants	Was anyone else present besides the participants and researchers?<br><br>No – see page 4
16.	Description of sample	What are the important characteristics of the sample? *e.g. demographic data, date*<br><br>As stated on page 4, the sample was made up of clinical academics in the UK
Data collection		
17.	Interview guide	Were questions, prompts, guides provided by the authors? Was it pilot tested?<br><br>As stated on page 4, and within the appendix, guidance was provided for audio diaries, interviews used piloted stems

For peer review only - http://bmjopen.bmj.com/site/about/guidelines.xhtml

No	Item	Guide questions/description
18.	Repeat interviews	Were repeat interviews carried out? If yes, how many?<br><br>As stated on page 4 no repeat interviews were carried out
19.	Audio/visual recording	Did the research use audio or visual recording to collect the data?<br><br>As stated on page 4 the research used audio recording to collect data
20.	Field notes	Were field notes made during and/or after the interview or focus group?<br><br>Yes, as stated on page, making of field notes was an ongoing process (during and after interviews), as part of reflexivity
21.	Duration	What was the duration of the interviews or focus group?<br>As stated on page…, the interviews lasted between 45 to 60 mins
22.	Data saturation	Was data saturation discussed?<br>Yes, the authors discussed data saturation on page 5.
23.	Transcripts returned	Were transcripts returned to participants for comment and/or correction?<br>As stated on page 5, transcripts were returned to participants for comment and/or correction upon request
		**Domain 3: analysis and findings**
Data analysis		
24.	Number of data coders	How many data coders coded the data?<br>As stated on page 4/5, 5 researchers coded the data
25.	Description of the coding tree	Did authors provide a description of the coding tree?<br>As stated on page 5, this description is listed on our website.
26.	Derivation of themes	Were themes identified in advance or derived from the data?

For peer review only

No	Item	Guide questions/description
		As detailed on page 5 themes were identified using a mixture of deductive and inductive methods
27.	Software	What software, if applicable, was used to manage the data?<br>As stated on page 5 NVivo was used manage the qualitative data
28.	Participant checking	Did participants provide feedback on the findings?<br>Yes, this is stated on page 5.
Reporting		
29.	Quotations presented	Were participant quotations presented to illustrate the themes / findings? Was each quotation identified? e.*g. participant number*<br><br>Yes participant quotations were presented throughout the RESULTS section to illustrated the associated themes
30.	Data and findings consistent	Was there consistency between the data presented and the findings?<br><br>Yes, this is stated on within the results and discussion sectons.
31.	Clarity of major themes	Were major themes clearly presented in the findings?<br><br>Yes, the themes are provided in the table 2.
32.	Clarity of minor themes	Is there a description of diverse cases or discussion of minor themes?<br><br>Yes, this description is provided in the discussion section (page 19 onwards)

For peer review only - http://bmjopen.bmj.com/site/about/guidelines.xhtml
